# *Lotus japonicus* VIH2 is an inositol pyrophosphate synthase that regulates arbuscular mycorrhiza

**DOI:** 10.1126/sciadv.aec5607

**Published:** 2026-05-22

**Authors:** Kiran Raj, Verena Gaugler, Mengsi Lu, Maren Schädel, Philipp Gaugler, Charlotte M. M. Grothaus, Ulrike A. Jochimsen, Athanasios Makris, Simon M. Bartsch, Anna M. Frentzen, Guizhen Liu, Michael Harings, Dorothea Fiedler, Henning J. Jessen, Gabriel Schaaf, Martina K. Ried-Lasi

**Affiliations:** ^1^ Symbiosis Signalling Group, Department of Molecular Signal Processing, Leibniz Institute of Plant Biochemistry, 06120 Halle (Saale), Germany.; ^2^ Institute of Crop Science and Resource Conservation (INRES), University of Bonn, 53115 Bonn, Germany.; ^3^ Institute of Organic Chemistry and the Center for Integrative Biological Signalling Studies (CIBSS), Albert-Ludwigs-University of Freiburg, 79104 Freiburg, Germany.; ^4^ Department of Chemical Biology, Leibniz-Forschungsinstitut für Molekulare Pharmakologie, 13125 Berlin, Germany.; ^5^ Institute of Chemistry, Humboldt-Universität zu Berlin, 12489 Berlin, Germany.

## Abstract

Plant yield is often maximized by the extensive use of mineral fertilizers, which, however, has severe environmental consequences. Phosphate is particularly problematic as it represents a globally limited resource, and its runoff and soil erosion threaten open water bodies. Many crops engage in arbuscular mycorrhizal (AM) symbiosis with nutrient-acquiring fungi, aiding in the uptake of phosphate and other mineral nutrients. However, AM colonization is strongly reduced under high soil phosphate levels. A mechanistic understanding of phosphate sensing, phosphate starvation responses, and their connection to AM remains incomplete. Here, we show that, in *Lotus japonicus*, low-abundant, energy-rich inositol pyrophosphates act as important regulatory signals of AM, orchestrating the cross-talk between phosphate starvation responses, nutrient acquisition, and plant root endosymbiosis. These findings hold promise for breeding nutrient-efficient crops.

## Teaser

Inositol pyrophosphate messengers regulate arbuscular mycorrhizal symbiosis and phosphate uptake in *Lotus japonicus*.

## INTRODUCTION

The macronutrient phosphorus (P) is a key determinant of crop performance and yield. Inorganic phosphate (P_i_), the P-form taken up by plants, quickly becomes immobilized in most soils, making the application of P-fertilizers critical to maintain crop productivity (*1*). However, P-fertilization comes with environmental costs as global P-deposits are limited. Furthermore, soil erosion contributes to eutrophication of open water bodies with P, a major stressor on marine ecosystems (*2*).

Plants respond to P_i_ limitations through local and systemic phosphate starvation responses (PSRs) that involve morphological, transcriptional, and metabolic changes. Local responses primarily alter root system architecture, while systemic responses regulate P_i_ homeostasis (*1*). *Arabidopsis* phosphate starvation response regulator 1 [PHR1; (*3*)] and related transcription factors [PHR1-likes; (*4*–*6*)] are key regulators of PSRs, and orthologs have been identified in several plant species (*7*–*11*). While under P_i_-limiting conditions, PHR1 targets specific motifs in the promoters of P_i_ starvation–induced (PSI) genes, thus activating their expression (*3*), under P_i_ sufficiency, PHR1 interacts with SYG1/Pho81/XPR1 (SPX) domain–containing proteins, which prevents the binding to its promoter core sequences (*12*–*17*). SPX domains are selective high-affinity receptors for inositol pyrophosphates (PP-InsPs), which mediate the interaction between SPX and PHR in PSR regulation (*18*–*20*).

PP-InsPs (such as InsP_7_ and InsP_8_) derive from inositol hexakisphosphate (phytate, InsP_6_), which serves as an important P-storage molecule in seeds and likely also in the whole plant (*21*). They are energy-rich, low-abundant messengers found in all eukaryotes (*22*) and act as proxies for plant P_i_ status (*20*, *23*–*25*). In *Arabidopsis*, the two bifunctional adenosine 5′-triphosphate (ATP)–grasp kinase/phosphatase enzymes and VirE2-interacting protein 1 (Vip1) homologs Vip1-homolog 1 (VIH1) and VIH2 catalyze the phosphorylation of InsP_6_ to 1/3-InsP_7_ and 1/3,5-InsP_8_ (*23*, *24*, *26*, *27*). VIHs are not only implicated in the generation of PP-InsPs but also have been proposed to contribute to their removal under varying P_i_ concentrations. For instance, the insect-purified C-terminal phosphatase domain of VIH2 hydrolyzed 1-InsP_7_ and 5-InsP_7_ to InsP_6_. Additionally, based on the biochemical activity of yeast Vip1, it has been proposed that low adenylate energy charge [i.e., ATP/adenosine 5′-diphosphate (ADP) ratios] stimulates the activity of the phosphatase domain of these proteins (*23*). Moreover, fungal Vip1 and Asp1 have been shown to hydrolyze 1,5-InsP_8_ to 5-InsP_7_ (*28*–*30*). These observations put forward the idea that VIHs might shift their activities to fine-tune energy metabolism and consequently adapt to adverse conditions (*31*). Likewise, the InsP_6_ kinase ITPK1, which generates 5-InsP_7_ under high ATP levels, is also able to transfer the β-phosphate group from 5-InsP_7_ to ADP under conditions of low adenylate energy charge, thereby regenerating InsP_6_ and ATP and effectively catalyzing the reverse reaction of its kinase activity (*24*, *32*).

In addition to local and systemic PSRs, plants form symbiotic associations with microbes to alleviate P_i_ deficiency. Up to 80% of the land plant species, including major crops such as maize, rice, and wheat, engage in arbuscular mycorrhiza (AM), a mutualistic relationship with Glomeromycota fungi (*33*). AM, which originated ~450 million years ago, likely contributed to land colonization (*34*, *35*) and influenced climate transition by reducing atmospheric CO_2_ levels, thereby lowering global temperatures and increasing atmospheric oxygen (*36*). AM is a form of endomycorrhiza, where the fungus resides intracellularly within plant roots, establishing a connection between the plant’s root system and the extensive extraradical mycelium of the AM fungus (*34*). Upon symbiotic stimulation, a prepenetration apparatus forms, guiding the fungus through the root epidermis into deeper cell layers (*37*). Once the cortex is reached, fungal hyphae enter the apoplastic space, where they grow longitudinally and branch out to penetrate the inner cortex cells, forming tree-shaped structures known as arbuscules (*38*, *39*). These arbuscule-containing cells serve as the primary sites for carbon-to-nutrient exchange between the plant and the fungus (*33*). Recent evidence indicates that PSRs and AM symbiosis are interconnected through the SPX/PHR module (*10*, *11*, *40*–*43*). PHR regulates a network of genes controlling AM development and symbiotic P_i_ uptake (*11*, *41*–*45*) and specific SPX proteins fine-tune fungal colonization (*10*, *41*, *42*). Moreover, in *Medicago truncatula*, SPX and PHR are involved in arbuscule maintenance (*10*, *43*).

Plants supply their fungal partner with up to 20% of their photosynthetically fixed carbon in the form of sugars and fatty acids (*33*, *46*–*48*). In return, AM enhances plant acquisition of P_i_, nitrogen, sulfur, trace elements, and water through the specialized hyphal network of the fungus (*49*) while also increasing plant tolerance to various biotic and abiotic stresses (*50*). However, this potential is constrained because even moderate P_i_ levels can systematically inhibit AM colonization (*51*, *52*), likely as a strategy to conserve photosynthates. Moreover, the widespread use of mineral fertilizers in high-input agriculture provides sufficient nutrients to plants, reducing their reliance on AM fungi. A deeper understanding of the molecular mechanisms governing these complex mutualistic associations with phosphate-acquiring microbes and their integration with nutrient homeostasis could be instrumental in breeding crop cultivars with improved nutrient use efficiency. Given the central role of VIH-dependent PP-InsP signaling in phosphate sensing and PSRs, we hypothesized that VIH2 also contributes to the regulation of arbuscular mycorrhizal symbiosis. Here, we investigate the role of *Lotus japonicus* VIH2 in integrating nutrient homeostasis with symbiosis signaling.

## RESULTS

### *L. japonicus* VIH2 is a functional Vip1-type PP-InsP synthase

To study the potential role of PP-InsPs in the regulation of AM and to interrogate the interconnection of P_i_ homeostasis and AM by InsP signaling in *L. japonicus*, we carried out a phylogenetic analysis of VIH homologs in algae and land plants and identified two *L. japonicus* VIH homologs (fig. S1). In *Arabidopsis*, VIH2 is the predominant InsP_8_ synthase and is broadly expressed across tissues, whereas VIH1 shows largely floral expression (*26*). Similarly, one *L. japonicus* VIH homolog, which we named VIH2, is expressed in roots and leaves, while expression of the other one is largely confined to flowers [*Lotus* Base, Expression Atlas; (*53*)]. Given our interest in P_i_-dependent regulation of AM in roots and the systemic contribution from shoots, we focused our study on the putative *L. japonicus* homolog of *Arabidopsis* VIH2.

**Fig. 1.**
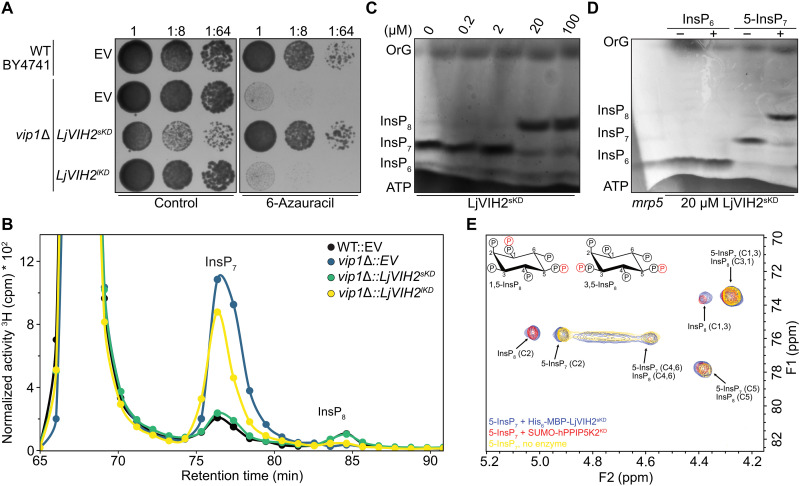
*L. japonicus* VIH2 is a functional Vip1-type PP-InsP synthase. The *S. cerevisiae* BY4741 wild type (WT) or a *vip1*Δ mutant strain was transformed with the empty vector (EV) or with plasmids encoding a short (LjVIH2^sKD^) or a long (LjVIH2^lKD^) splice variant of the isolated *L. japonicus* VIH2 kinase domain. (**A**) The indicated yeast strains were spotted in eightfold serial dilutions onto selective growth medium with or without 6-azauracil. (**B**) Normalized high-performance liquid chromatography (HPLC) profiles of InsPs of extracts from [^3^H]-*myo*-inositol–labeled yeast transformants either carrying the empty vector (EV) or ectopically expressing *LjVIH2^sKD^* or *LjVIH2^lKD^*. Extracts were resolved by strong anion exchange (SAX)–HPLC, and fractions were collected each minute for subsequent determination of radioactivity. (**C** and **D**) His_6_-MBP-LjVIH2^sKD^ was expressed in *E. coli* and purified via Ni–nitrilotriacetic acid (NTA) resin. (C) 5-InsP_7_ (0.33 mM) was incubated with increasing concentrations (0 to 100 μM) of His_6_-MBP-LjVIH2^sKD^ for 1 hour. (D) InsP_6_ or 5-InsP_7_ (0.33 mM) was incubated with (+) or without (−) 20 μM recombinant His_6_-MBP-LjVIH2^sKD^ at 22°C for 1 hour. TiO_2_-purified *Arabidopsis mrp5* seed extracts, which accumulate high amounts of InsP and InsP_8_, were used as a marker for InsP_6_, InsP_7_, and InsP_8_. [(C) and (D)] InsPs were mixed with a loading buffer containing OrG as a loading dye, separated by polyacrylamide gel electrophoresis (PAGE) and visualized by toluidine blue staining. (**E**) ^1^H-^13^C–heteronuclear multiple-quantum coherence (HMQC) spectra depicting the reaction products when 5-InsP_7_ was incubated with LjVIH2^sKD^ (blue traces, 7 hours) or SUMO-hPPIP5K2^KD^ (red traces, overnight). The 5-InsP_7_ reference spectrum (without enzyme) is shown in yellow. Carbon assignments (C1 to C6) refer to the respective carbon positions of the *myo*-inositol ring detected in the ^1^H-^13^C-HMQC spectra. Structural representations of 1,5-InsP_8_ and 3,5-InsP_8_ are included for reference. cpm, counts per minute; OrG, Orange G; ppm, parts per million.

To assess its enzymatic activity, we heterologously expressed a long and a six–amino acid shorter splice variant of its isolated kinase domain (fig. S2) as translational fusions with a C-terminal V5-tag in a *Saccharomyces cerevisiae vip1*Δ knockout mutant, which is unable to grow on 6-azauracil (*54*). Both splice variants were detected in *L. japonicus* cDNA preparations, motivating us to directly compare their enzymatic functionality. While both variants were expressed in yeast (fig. S3), only the short variant of the isolated *L. japonicus VIH2* kinase domain restored growth of the *S. cerevisiae* mutant on the selection medium (Fig. 1A). In yeast, Vip1 generates 1-InsP_7_ from InsP_6_ and 1,5-InsP_8_ from 5-InsP_7_ (*30*, *55*).

While the *S. cerevisiae vip1*Δ knockout strain no longer produces detectable amounts of InsP_8_ Fig. 1B (*56*), expression of the short splice variant of the isolated *L. japonicus* VIH2 kinase domain restored a clear InsP_8_ signal in normalized strong anion exchange high-performance liquid chromatography (SAX-HPLC) profiles of ^3^H-*myo*-inositol–labeled *vip1*Δ transformants, without markedly altering the remainder of the InsP profile (Fig. 1B and fig. S4). Last, the short splice variant of the isolated *L. japonicus* VIH2 kinase domain recombinantly expressed and purified from *Escherichia coli*–converted 5-InsP_7_ to InsP_8_ in vitro (Fig. 1, C and D). Consistent with previous work showing that PPIP5K-family VIH homologs from mammals, yeast, and *Arabidopsis* preferentially phosphorylate the 1/3-position of 5-InsP_7_ to generate 1/3,5-InsP_8_ (*23*, *30*, *57*), we qualitatively assessed the substrate specificity of LjVIH2^sKD^ by ^1^H-^13^C–heteronuclear multiple-quantum coherence (HMQC)–nuclear magnetic resonance (NMR) using ^13^C_6_-labeled 5-InsP_7_. Comparison with spectra of untreated 5-InsP_7_ and mammalian PPIP5K2-treated samples confirmed that LjVIH2^sKD^ synthesizes 1/3,5-InsP_8_ from 5-InsP_7_, similar to its plant homolog AtVIH2^KD^ (Fig. 1E). Our data show that VIH2 is a functional Vip1-type PP-InsP synthase and catalyzes the conversion of InsP_7_ to InsP_8_ in yeast and in vitro (Fig. 1).

### A hydroponic cultivation system to study PSRs and PP-InsP signaling in *L. japonicus*

**Fig. 2.**
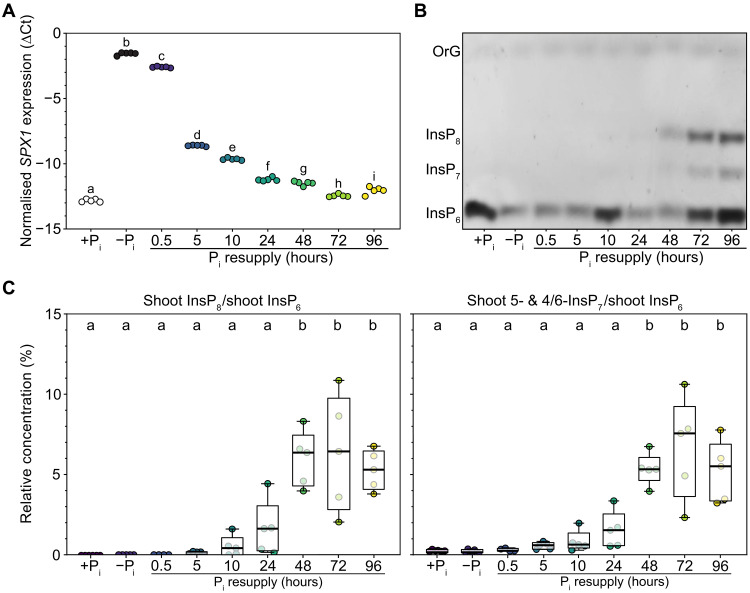
A hydroponic cultivation system to study PSRs and PP-InsP signaling in *L. japonicus*. *L. japonicus* wild-type seeds were germinated, and seedlings were grown hydroponically in +P_i_ liquid medium containing 1500 μM P_i_ for 4 weeks. Subsequently, plants were either transferred to −P_i_ liquid medium, starved for 8 days (−P_i_), followed by transfer back to +P_i_ liquid medium for P_i_ resupply for 0 to 96 hours (P_i_ resupply), or kept on +P_i_ liquid medium for the whole time (+P_i_). (**A**) The expression level of the PSI marker gene *SPX1* was analyzed in shoots via quantitative reverse transcription polymerase chain reaction (qRT-PCR). ∆Ct, delta cycle threshold; *n* = 5. (**B** and **C**) InsPs were enriched by TiO_2_ pull-down and (B) mixed with a loading buffer containing OrG as a loading dye, resolved by PAGE for qualitative illustration of InsP profiles and visualized by toluidine blue staining or (C) quantified via capillary electrophoresis–electrospray ionization mass spectrometry (CE-ESI-MS) analysis. Shoot PP-InsP levels are presented relative to shoot InsP_6_. *n* = 4 to 5. [(A) and (C)] For statistical analysis, an ordinary one-way analysis of variance (ANOVA) with Tukey’s multiple comparisons test was performed. Different letters indicate significant differences (*P* ≤ 0.05). OrG, Orange G.

To assess InsP levels, we established a hydroponic system that allows the cultivation of *L. japonicus* under different nutrient regimes (fig. S5). *L. japonicus* plants were grown either with sufficient P_i_ or subjected to P_i_ starvation for 8 days. Additionally, P_i_-starved plants were resupplied with P_i_ for 0.5 to 96 hours (Fig. 2) as P_i_ resupply experiments are effective for investigating defects in PP-InsP synthesis by minimizing compensatory effects that occur under steady-state conditions (*24*). The expression of the PSI marker gene *SPX1* was strongly up-regulated upon P_i_ starvation and decreased significantly within 0.5 hours of P_i_ resupply (Fig. 2A), confirming successful P_i_ starvation and resupply.

Qualitative visualization of InsP species using polyacrylamide gel electrophoresis (PAGE) followed by toluidine blue staining revealed detectable InsP_7_ and InsP_8_ bands when P_i_-starved plants were resupplied with P_i_ for 48 to 96 hours (Fig. 2B). The PAGE analysis provides a qualitative overview of bulk InsP profiles but does not resolve individual InsP isomers. Quantitative analysis of InsP levels was, therefore, performed using capillary electrophoresis–electrospray ionization mass spectrometry (CE-ESI-MS), which revealed dynamic changes in shoot and root InsP profiles upon Pi resupply (*58*–*61*) (Fig. 2C and figs. S6 and S7). In shoots, the relative amounts of InsP_8_ and 1- and 5-/4/6-InsP_7_ increased more than 25-fold, i.e., from 0 to 0.2% reaching 5 to 7% of InsP_6_ levels by 48 to 96 hours after P_i_ resupply (Fig. 2C and fig. S6A). Conversely, levels of InsP_3-2_ decreased markedly during this period (fig. S6A). In roots, relative amounts of InsP_8_ and 1- and 5-/4/6-InsP_7_ also rose significantly, albeit to a lower extent, i.e., from 0 to 0.2% to 0.5 to 1.0% of InsP_6_ by 72 to 96 hours (fig. S7A). Additional root InsPs, including InsP_5_ (4/6-OH), 2,3,4,5-InsP_4_, and InsP_3-1_ showed notable increases, while InsP_3-2_ levels declined consistently in roots over the 96-hour period (fig. S7A). Notably, InsP_6_ levels increased approximately threefold in shoots but remained largely unchanged in roots (figs. S6B and S7B). In our measurements, InsP_8_ levels in shoots and roots began to increase from 5 hours after P_i_ resupply onward, although these changes did not reach statistical significance due to variance and sample size limitations (Fig. 2C and fig. S7A). Nevertheless, the absence of detectable InsP_8_ at 0 and 0.5 hours after P_i_ resupply and the consistent increase thereafter suggest that accumulation likely starts from around 5 hours after P_i_ resupply. This temporal pattern broadly coincides with the early changes in PSR marker gene expression, although additional signaling molecules such as 5-InsP_7_ and/or 1/3-InsP_7_ or parallel pathways may contribute to the initial transcriptional response. These results are in line with previous observations in *Arabidopsis* (*24*) and highlight the suitability of the hydroponic system for studying InsP dynamics in *L. japonicus*.

Earlier studies in *Arabidopsis* showed that ATP levels correlate with the P_i_ status (*23*, *24*). To investigate this in *L. japonicus*, we analyzed shoot ATP levels in hydroponically grown wild-type plants cultivated with sufficient P_i_, subjected to P_i_ starvation for 8 days, or resupplied with P_i_ for 72 hours after P_i_ starvation (fig. S8). Similarly, as in *Arabidopsis*, we find that P_i_ deficiency caused a reduction in ATP levels, which are restored upon P_i_ resupply (fig. S8), suggesting a conserved mechanism across plants.

### *L. japonicus vih2* mutants have altered InsP/PP-InsP levels and show enhanced, constitutive PSRs

**Fig. 3.**
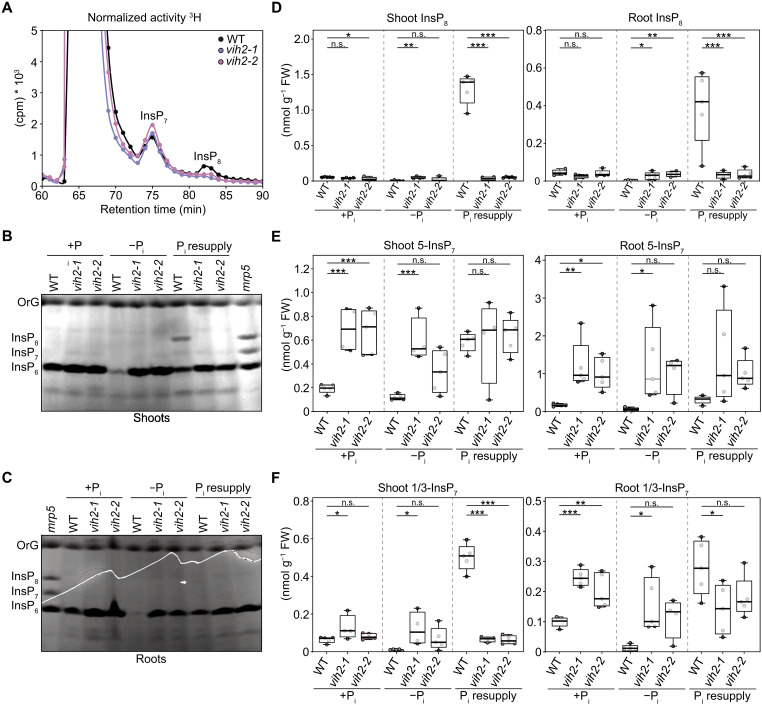
*L. japonicus vih2* mutants have altered InsP/PP-InsP levels and show enhanced, constitutive PSRs. (**A**) *L. japonicus* wild-type (WT) and *vih2* mutant seeds were germinated and subsequently grown for 14 days on water agar plates before transfer to liquid half-strength Murashige-Skoog medium supplemented with 1% sucrose and 45 μCi of [^3^H]-*myo*-inositol for 5 days. InsPs were extracted and separated by SAX-HPLC. cpm, counts per minute. (**B** to **F**) *L. japonicus* wild-type (WT) and *vih2* mutant seeds were germinated, and seedlings were grown in +P_i_ liquid medium containing 1500 μM P_i_ for 4 weeks. Subsequently, plants were either transferred to −P_i_ liquid medium, starved for 10 days (−P_i_), followed by transfer back to +P_i_ liquid medium for P_i_ resupply for 72 hours (P_i_ resupply), or kept on +P_i_ liquid medium for the whole time (+P_i_). Shoot and root InsPs were enriched by TiO_2_ pull-down and [(B) and (C)] mixed with a loading buffer containing OrG as a loading dye, resolved by PAGE for qualitative illustration of InsP profiles and visualized by toluidine blue staining or (D to F) quantified via CE-ESI-MS analysis. TiO_2_-purified *Arabidopsis mrp5* seed extracts, which accumulate high amounts of InsP_7_ and InsP_8_, were used as a marker for InsP_6_, InsP_7_, and InsP_8_. Arrow points to the band corresponding to InsP_8_ in wild-type shoots under P_i_ resupply. The white line (zig-zag boundary) marks a physical tear in the gel introduced during handling and does not affect data interpretation. *n* = 4 to 5. For statistical analysis, an ordinary one-way ANOVA with Dunnett’s multiple comparisons test were performed. n.s., not significant; *P ≤ 0.05; **P ≤ 0.01; ***P ≤ 0.001. OrG, Orange G; FW, fresh weight.

To study the potential role of PP-InsPs in the regulation of AM and to interrogate the interconnection of P_i_ homeostasis and AM by InsP signaling in *L. japonicus*, we used two independent homozygous LORE1 transposon insertion lines (*53*, *62*) in *VIH2* (fig. S9) and investigated potential changes in InsP/PP-InsP levels (Fig. 3 and figs. S10 to S12) and associated deregulation of PSRs (fig. S13). In *Arabidopsis*, InsP_8_ serves as a proxy for P_i_, and defects in its synthesis result in PSR-related phenotypes (*20*, *23*). While a clear signal corresponding to InsP_8_ was previously detected in the normalized SAX-HPLC profile of InsPs of extracts from [^3^H]-*myo*-inositol–labeled *L. japonicus* wild-type seedlings (*63*), we demonstrate that the *L. japonicus vih2*-1 and *vih2*-*2* mutants showed strongly reduced levels of InsP_8_ while, otherwise, retaining an InsP profile broadly similar to the wild type (Fig. 3A and fig. S14).

Qualitative analysis of InsP levels in hydroponically grown plants by PAGE confirmed a pronounced reduction in InsP_8_ in the shoots and roots of both independent *vih2* mutants under P_i_ resupply conditions (Fig. 3, B and C, and fig. S10). Quantitative, isomer-resolved analysis by CE-ESI-MS confirmed this reduction and further revealed a substantial concomitant decrease in shoot 1/3-InsP_7_ under these conditions. In addition, CE-ESI-MS analysis showed increased levels of InsP_4_, InsP_5_, InsP_6_, 4/6-InsP_7_, and 5-InsP_7_ in shoots under P_i_ deficiency and in roots under both P_i_ sufficiency and P_i_ deficiency (Fig. 3, D to F, and figs. S11 and S12). While synthesis of InsP_8_ upon P_i_ starvation and resupply in *L. japonicus* shoots is comparable to *Arabidopsis* shoots, production of InsP_8_ in *L. japonicus* roots was markedly increased in comparison to *Arabidopsis* roots (Fig. 3C and fig. S10) (*24*).

As indicated by the expression of the PSI marker gene *SPX1*, PSR in *vih2* mutant lines was constitutively activated at both high (1500 μM) and low (25 μM) external P_i_ (fig. S13). Although *SPX1* expression was still repressed in *vih2* mutants under high P_i_, this can be explained by two factors. First, InsP_8_ is not completely abolished in the mutants, likely due to redundancy with VIH1. Second, 5-InsP_7_ has been shown in *Arabidopsis* to partially repress PSR by binding to SPX, albeit with lower affinity than InsP_8_ (*24*). Consistent with this, 5-InsP_7_ levels are not compromised in *vih2* mutants and even increase under +P_i_ conditions, supporting a role in residual repression of PSR. However, *vih2*-*1* and *vih2*-*2* mutants only accumulated significantly more shoot P than wild-type plants when grown in the presence of high (1500 or 2500 μM) external P_i_, but not when cultivated with low to medium (25 to 750 μM) external P_i_ (fig. S13). A slight reduction in shoot fresh weight was detectable for *vih2-1* mutants compared with wild-type plants when grown in 2500 μM P_i_ for 4.5 weeks, but not when plants were cultivated in the presence of 25 to 1500 μM P_i_ (fig. S15).

### Attenuation of VIH2 activity stimulates AM colonization and P_i_ uptake at a large range of external P conditions

**Fig. 4.**
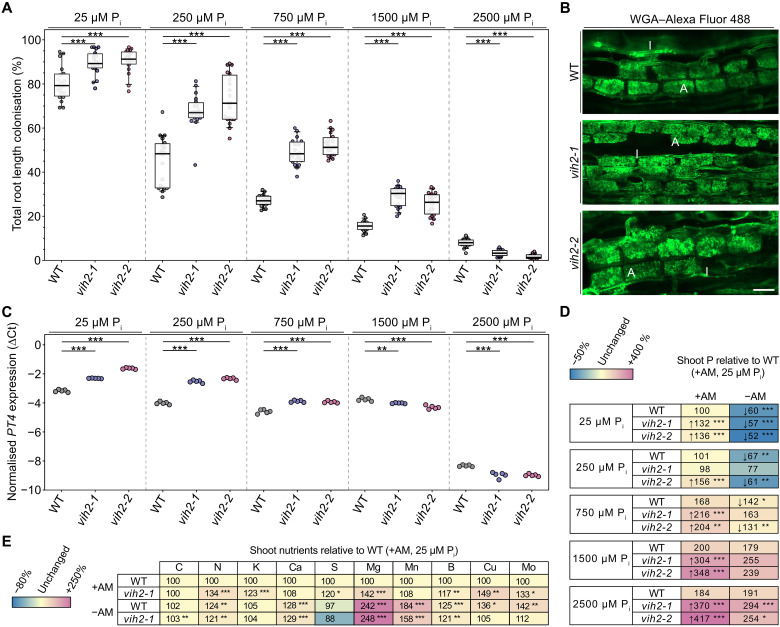
Attenuation of VIH2 activity stimulates AM colonization even under high external P_i_. Ten-day-old *L. japonicus* wild-type (WT) and *vih2* mutant seedlings were planted in open pots containing 300 ml of washed sand (five seedlings per pot) and grown in the presence of *R. irregularis* spore inoculum (Symplanta, Germany; 500 spores per plant). Plants were fertilized once a week with liquid *Lotus* cultivation medium containing the indicated P_i_ concentrations and harvested 4.5 weeks after planting. (**A**) Roots inoculated with AM fungal spores were stained with ink-vinegar, and AM colonization was quantified. *n* = 20. (**B**) Roots inoculated with AM fungal spores were stained with wheat germ agglutinin (WGA) conjugated to Alexa Fluor 488 and observed with a confocal microscope. Representative pictures are shown. Scale bar, 50 μm. A, arbuscule; I, intraradical hypha. (**C**) The expression level of the AM marker gene *PT4* was analyzed in roots inoculated with AM fungal spores via qRT-PCR. ∆Ct, delta cycle threshold; *n* = 5. (**D**) Shoot P levels were determined via ICP-OES analysis. Data are presented relative to P levels in wild-type shoots of plants inoculated with AM fungal spores and grown in the presence of 25 μM P_i_, which were set to 100%. *n* = 5 to 9. Numbers indicate percentages. Stars indicate significant differences to the corresponding +AM wild-type measurements. (**E**) Shoot nutrient levels were determined via ICP-OES or C and N elemental analysis. Data are presented relative to nutrient levels in wild-type shoots of plants inoculated with AM fungal spores grown in the presence of 25 μM P_i_, which were set to 100%. *n* = 5 to 9. Numbers indicate percentages. Stars indicate significant differences to the corresponding wild-type measurements. [(A) and (C) to (E)] For statistical analysis, an ordinary one-way ANOVA with Dunnett’s multiple comparisons test was performed. *P ≤ 0.05; **P ≤ 0.01; ***P ≤ 0.001.

To investigate whether altered InsP/PP-InsP synthesis (Fig. 3) influences AM colonization, we grew *L. japonicus* wild-type and *vih2* mutant seedlings in a sand-based open pot system and inoculated them with *Rhizophagus irregularis* spores. Thirty days after inoculation, we harvested the root systems and quantified AM colonization. Intriguingly, *L. japonicus vih2* mutants showed significantly increased total, arbuscule, and vesicle root length colonization across a large range of external P_i_ concentrations (25 to 1500 μM P_i_), indicating that VIH2-dependent InsPs/PP-InsPs attenuate AM colonization at both high and low external P_i_ levels. (Fig. 4, A and B; and fig. S16, A to C). Arbuscules in *vih2* roots were morphologically comparable to those formed in wild-type roots (Fig. 4B and fig. S16C). This was in line with the expression levels of the two AM marker genes, *PT4* and *SbtM1*, which were significantly increased in the *vih2* mutants compared with the wild type at external P_i_ concentrations of 25 to 750 μM (Fig. 4C and fig. S16D). At 1500 μM P_i_, *SbtM1* and *PT4* transcript levels were lower than expected from colonization percentages, possibly reflecting increased arbuscule degradation at this concentration (Fig. 4C and fig. S16D).

Moreover, AM colonization strongly enhanced P acquisition in *vih2* mutant lines. Even under low P_i_ conditions (25 μM), both independent *vih2* lines showed a substantial increase in shoot P compared with the wild type. This effect was further amplified under P_i_-sufficient conditions, resulting in approximately a twofold increase in shoot P compared with wild-type plants, suggesting an additive effect of AM-dependent and AM-independent *vih2*-associated increases in shoot P accumulation (Fig. 4D).

To determine whether this effect extended to other nutrients, we analyzed the accumulation of additional mineral elements in the shoot (Fig. 4E). Under AM-colonized conditions, *vih2* plants accumulated higher levels of several nutrients, including nitrogen, potassium, magnesium, and copper, compared with wild-type plants. When comparing AM-colonized and noncolonized plants, the response varied between elements: While some nutrients remained elevated in *vih2* plants under AM conditions, others were similar or lower relative to the corresponding non-AM condition (Fig. 4E). Together, these results indicate that the *vih2* mutation promotes increased nutrient accumulation particularly in the context of AM colonization, although the overall effect of AM symbiosis on individual nutrients differs between elements.

To investigate whether the *vih2* mutants were also better colonized by AM fungi than the wild type at earlier stages of AM development, we investigated total, arbuscule, and vesicle root length colonization when the wild type had a mean total root length colonization of 20 and 40%, respectively (fig. S17, A and D). Already at earlier time points, the *vih2* mutants were significantly better colonized than the wild type (fig. S17, A and D). The arbuscule morphology was similar in the roots of all tested genotypes (fig. S17, B and E). Enhanced AM colonization in the *vih2* mutants was accompanied by increased expression levels of the AM marker gene *PT4* (fig. S17, C and F).

Both *vih2* alleles showed concordant phenotypes, reduced InsP_8_ accumulation, increased AM colonization under different external P_i_, and PSR derepression, supporting a role for VIH2 in InsP_8_-dependent regulation. Minor quantitative differences likely reflect allele strength.

### Attenuation of VIH2 activity promotes the formation and maintenance of mature arbuscules

**Fig. 5.**
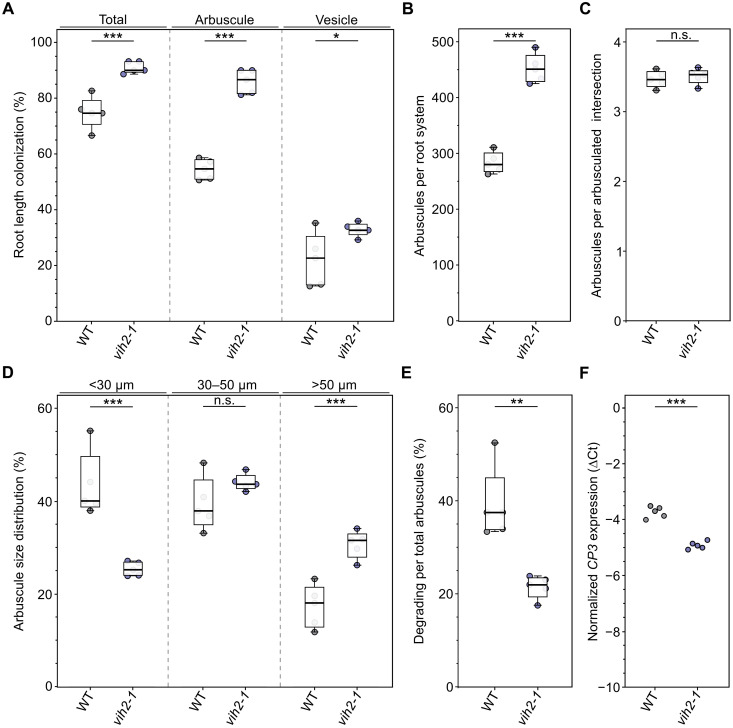
*L. japonicus vih2* mutants have significantly more arbuscules and significantly less degrading arbuscules than wild-type plants. Ten-day-old *L. japonicus* wild-type (WT) and *vih2* mutant seedlings were planted in open pots containing 300 ml of washed sand (five seedlings per pot) and inoculated with *R. irregularis* spores (Symplanta, Germany; 500 spores per plant). Plants were fertilized once a week with liquid *Lotus* cultivation medium containing 25 μM P_i_ and harvested 4.5 weeks after planting. Roots were stained with WGA conjugated to Alexa Fluor 488 and observed with a confocal microscope. Total, arbuscule, and vesicle root length colonization (**A**); arbuscule number per root system (**B**); the size distribution of arbuscules per root system (**C**); the percentage of degrading per total arbuscules per root system (**D**); as well as the number of arbuscules per arbusculated intersection per root system (**E**) were quantified. *n* = 5. (**F**) The expression level of the AM marker gene *CP3* was analyzed in roots via qRT-PCR. ∆Ct, delta cycle threshold; *n* = 5. [(A) to (F)] For statistical analysis, an unpaired, two-tailed *t* test was performed. n.s., not significant; *P < 0.05; **P ≤ 0.01; ***P ≤ 0.001.

It has been reported previously that overexpression of *SPX1* and *SPX3* in *M. truncatula* not only enhances AM fungal colonization but also promotes arbuscule degradation (*43*). To assess whether similar effects occur in the *L. japonicus vih2* mutant, we examined root length colonization, arbuscule abundance and developmental status in the wild type and the *vih2*-*1* mutant grown under P_i_-deficient conditions (25 μM). Fungal structures were visualized at 30 days postinoculation by staining with wheat germ agglutinin (WGA) conjugated to Alexa Fluor 488 and analyzed via confocal microscopy (Fig. 5).

Consistent with ink-vinegar staining results, the *vih2*-*1* mutant exhibited significantly higher total root length colonization as well as increased arbuscule and vesicle abundance compared with wild type (Figs. 4A and 5; and figs. S16, A and B, S17, A and D). On average, wild-type root systems contained 283 arbuscules, whereas the *vih2-1* mutant showed a significant increase to 452 arbuscules per root system (Fig. 5B). The average number of arbuscules per arbusculated intersection and root system (~3.5) was comparable between genotypes, indicating that the elevated arbuscule number in the *vih2*-*1* mutant is due to enhanced arbuscule colonization along the root length (Fig. 5C).

We next examined arbuscule size distribution in wild-type and *vih2*-*1* mutant roots by measuring all arbuscules observed across 750 intersections (150 per root system) per genotype. Arbuscules were grouped into three size classes: <30 μm, 30 to 50 μm, and >50 μm. Notably, septa, indicative of arbuscule degradation, were only observed in arbuscules of <50 μm, suggesting that arbuscules of >50 μm represent mature structures, while smaller arbuscules likely reflect either developmental (no septa) or degenerative (with septa) stages. Compared with wild type, the *vih2*-*1* mutant showed a significant decrease in the proportion of arbuscules of <30 μm and a corresponding increase in mature arbuscules (>50 μm) (Fig. 5D). Last, the proportion of degrading arbuscules relative to the total number per root system was significantly lower in the *vih2*-*1* mutant compared with the wild type, indicating that enhanced AM fungal colonization did not compromise arbuscule maintenance nor promote premature degeneration (Fig. 5E). This is consistent with the expression pattern of *Cysteine Protease 3* (*CP3*), an AM-induced gene associated with arbuscule degradation (*64*), whose transcript levels were significantly higher in *L. japonicus* wild-type roots compared with the *vih2*-*1* mutant (Fig. 5F).

### Attenuation of VIH2 activity enhanced AM colonization and nutrient acquisition in field-collected topsoil

**Fig. 6.**
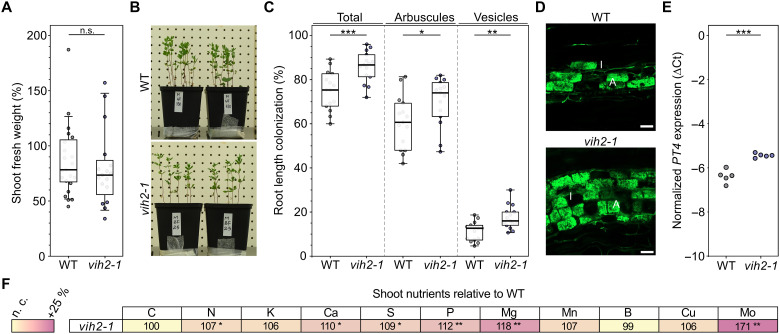
*L. japonicus vih2* mutants are significantly better colonized by AM fungi in field-collected topsoil than wild-type plants. Ten-day-old *L. japonicus* wild-type (WT) and *vih2* mutant seedlings were planted in open pots containing 300 ml of field-collected topsoil with moderate nitrogen, phosphorus, and potassium availabilities (five seedlings per pot) and were fertilized once with liquid *Lotus* cultivation medium containing 25 μM P_i_ and then once a week with Milli-Q water and harvested 5.5 weeks postplanting. (**A**) The shoot fresh weight per plant was measured. *n* = 17 to 21. (**B**) Representative pictures of plants analyzed in (A). (**C**) Roots were stained with ink-vinegar, and AM colonization was quantified. *n* = 15. (**D**) Roots were stained with WGA conjugated to Alexa Fluor 488 and observed with a confocal microscope. Representative pictures are shown. Scale bars, 50 μm. V, vesicles; A, arbuscule; I, intraradical hypha. (**E**) The expression level of the AM marker gene *PT4* was analyzed in roots via qRT-PCR. ∆Ct, delta cycle threshold; *n* = 5. (**F**) Shoot nutrient levels were determined via ICP-OES or C and N elemental analysis. Data are presented relative to nutrient levels in wild-type shoots, which were set to 100%. n. c., not changed; *n* = 5 to 9. Numbers indicate percentages. Stars indicate significant differences of the *vih2* mutant line in comparison to the corresponding wild-type measurements. [(A), (C), (E), (F)] For statistical analysis, an unpaired, two-tailed *t* test was performed. n.s., not significant; *P < 0.5; **P ≤ 0.01; ***P ≤ 0.001.

We next compared plant performance in a field-collected topsoil with moderate nitrogen, phosphorus, and potassium availability (table S3). Similar to plants grown in sterilized sand in the absence of AM fungal spores (fig. S15), the shoot fresh weight of the wild type and *vih2* mutant was comparable (Fig. 6A), and no obvious growth defects were detected for the *vih2* mutant when grown in nonsterile field-collected topsoil (Fig. 6B). Consistent with our sand-culture experiments using defined *R. irregularis* inoculation, *vih2* plants were significantly more highly colonized by naturally occurring AM fungi in the field soil (Fig. 6C). The morphology of arbuscules formed in *vih2* mutant roots was indistinguishable from that of arbuscules formed in wild-type roots (Fig. 6D).

In line with AM colonization, the expression level of the AM marker gene *PT4* was significantly increased in the *vih2* mutant compared with the wild type (Fig. 6E). Similar to *vih2* mutants grown in sand and fertilized with full-strength *Lotus* medium containing 25 μM P_i_ (Fig. 4E), we also observed a significant increase in phosphate, nitrogen, magnesium, sulfur, and molybdenum and additionally an increase in calcium when plants were grown in a field-collected topsoil with moderate nitrogen, phosphorus, and potassium availabilities (table S3 and Fig. 6F). Together, our data demonstrate that inactivation of VIH2 promotes arbuscule maintenance and AM fungal colonization from early stages of the interaction and translates into improved nutrient acquisition under both controlled nutrient supply and natural growth conditions.

### *L. japonicus* VIH2 controls AM colonization systemically and locally

**Fig. 7.**
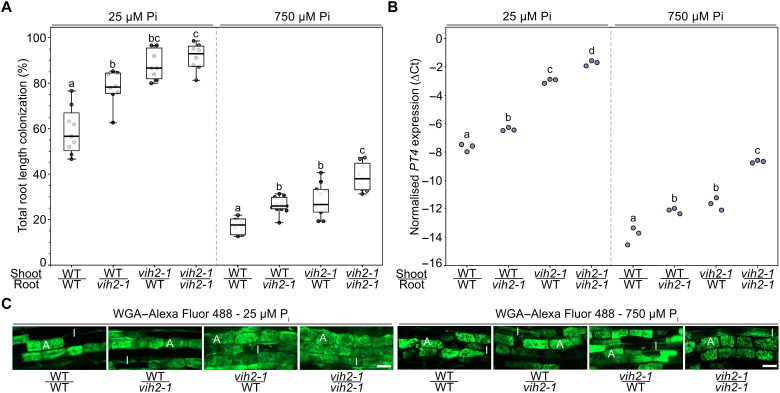
Colonization of *L. japonicus* by AM fungi is controlled systemically and locally by *VIH2*. Six-day-old AM and grown for 3 weeks on plates. Subsequently, successful grafts were planted in open pots containing 300 ml of washed sand (five seedlings per pot) and grown in the presence of *R. irregularis* spore inoculum (Symplanta, Germany; 500 spores per plant). Plants were fertilized once a week with liquid *Lotus* cultivation medium containing 25 μM P_i_ and harvested 5 weeks after planting. (**A**) Roots were stained with ink-vinegar, and AM colonization was quantified. *n* = 8 to 9. (**B**) The expression level of the AM marker gene *PT4* was analyzed in roots via qRT-PCR. ∆Ct, delta cycle threshold; *n* = 3. [(A) and (B)] For statistical analysis, an ordinary one-way ANOVA with Tukey’s multiple comparisons test was performed. Different letters indicate significant differences (*P* ≤ 0.05). (**C**) Roots were stained with WGA conjugated to Alexa Fluor 488 and observed with a confocal microscope. Representative pictures are shown. Scale bars, 50 μm. A, arbuscule; I, intraradical hypha.

We then conducted grafting experiments under P_i_-deficient and sufficient conditions to determine whether colonization of *L. japonicus* by AM is regulated systemically or locally by VIH2. As expected, a strong increase in AM colonization was observed when roots and shoots of *vih2* plants were self-grafted compared with self-grafted wild-type plants (Fig. 7A and fig. S18, A and B). Notably, AM colonization also increased to a similar extent when *vih2* shoots were grafted onto wild-type roots for both tested P_i_ regimes, suggesting a strong systemic component in VIH2-dependent AM colonization (Fig. 7A and fig. S18, A and B).

Grafting wild-type shoots onto *vih2* mutant roots also resulted in a robust increase in AM colonization, which was not statistically significantly different from the reciprocal graft, indicating that VIH2 also controls AM colonization locally (Fig. 7A and fig. S18, A and B). The expression of the AM marker gene *PT4* largely correlated with total root length colonization (Fig. 7B). The morphology of arbuscules was comparable in all tested grafts (Fig. 7C).

## DISCUSSION

Resilience and yield stability are crucial aspects of modern agriculture, particularly as farmers face increasing environmental unpredictability. While maximizing yield has traditionally been the primary goal, there is growing recognition that stable yields over time are equally important. High yield variability poses notable risks to food security and economic stability, making resilience a desirable trait for sustainable farming practices (*65*).

Breeding efforts in the past have primarily focused on easily measurable traits such as disease resistance and drought tolerance. However, incorporating beneficial plant-microbe interactions, like those mediated by AM fungi, into breeding remains challenging as they are influenced by multiple factors, including plant genetics, microbial compatibility, and environmental conditions such as soil properties and nutrient availability (*66*). Plants have evolved mechanisms to suppress AM colonization under high P availability (*51*, *52*), thereby economizing on carbon allocation to the fungal symbiont, a strategy that appears to be ecologically advantageous particularly in natural environments, but not in agriculturally used land. Agricultural practices, including fertilization, soil tillage, and monocropping, have been shown to negatively affect AM fungal richness and community composition over the long term (*67*). As a consequence, farmland often exhibits reduced AM fungal diversity and abundance compared with natural ecosystems (*68*, *69*). Accordingly, breeding crops capable of sustaining AM colonization under high external P conditions could help preserve AM fungal abundance and diversity. By ensuring the availability of compatible fungal partners, such crops would support AM colonization across a broader range of P_i_ levels and enable the rapid reestablishment of symbiosis under adverse soil conditions. This, in turn, may contribute to symbiotic persistence and resilience in increasingly unpredictable agricultural environments. However, whether such effects translate into increased biomass accumulation or yield will require direct testing under agronomically relevant conditions.

Here, we show that *L. japonicus* VIH2 functions as a PP-InsP kinase and is largely responsible for the synthesis of InsP_8_, which has been shown to serve as a proxy for the P_i_ status in *Arabidopsis* (Figs. 1 and 3 and figs. S10 to S12) (*20*, *23*, *24*). We further demonstrate that VIH2 inactivation not only induces PSR (fig. S13) but also significantly enhances AM colonization (Fig. 4 and figs. S16 and S17). Notably, in the absence of *R. irregularis*, P_i_ overaccumulation occurred only under high external P_i_, but not at low or medium levels (fig. S13), reminiscent of phenotypes in *Arabidopsis* mutants deficient in InsP_8_ synthesis (*24*). Enhanced AM colonization, as measured by total root colonization, arbuscule abundance, and vesicle formation, was observed consistently across a wide range of external P_i_ concentrations (25 to 1500 μM) (Fig. 4 and figs. S16 and S17). We speculate that this increased colonization, even at low external P_i_, may stem from a strong local signaling component, independent of systemic P_i_ status, which facilitates AM establishment across varying P_i_ conditions. This phenotype might be driven by elevated VIH2 root activity in *L. japonicus* compared with *Arabidopsis*, as indicated by the stronger InsP_8_ response in *L. japonicus* roots following P_i_ resupply to P_i_-starved plants (Fig. 3, C and D, and fig. S10), a response absent in *Arabidopsis* (*24*). Supporting this hypothesis, grafting experiments confirmed that root-specific inactivation of VIH2 is sufficient to enhance AM colonization at low and high external P_i_ (Fig. 7A and fig. S18A).

While systemic regulation of the PSR is best characterized in mechanistic detail in *Arabidopsis*, available evidence indicates that core components of PSR signaling, including PHR transcription factors and SPX proteins, are conserved in legumes such as *M. truncatula* or *L. japonicus* (*10*, *11*, *43*, *70*), although aspects of P_i_ sensing and systemic regulation remain less well resolved. In *Arabidopsis*, the shoot-derived microRNA miR399 represses expression of the ubiquitin conjugase PHO2 in the root, thereby enhancing phosphate uptake and translocation (*71*–*73*). Reciprocal grafting experiments have demonstrated that miR399 can move long-distance from shoot to root, directly establishing its role as a systemic signal in the PSR (*73*). Together with the transcription factor PHR1, which acts upstream of miR399 and PHO2 (*72*), this module defines a conserved systemic signaling pathway that coordinates P_i_ homeostasis between roots and shoots. Homologs of miR399 and PHO2 are present in legumes such as *M. truncatula* (*74*), and P_i_-dependent regulation of miR399 has been demonstrated in species such as rice (*72*), suggesting that this module is broadly conserved in higher plants. However, the extent to which the miR399-PHO2 pathway interacts with symbiotic signaling pathways, such as AM symbiosis, remains poorly understood in legumes. Intriguingly, AM colonization is regulated by a sophisticated root-shoot-root signaling circuit known as autoregulation of mycorrhization [AOM; (*75*)]. Under low P_i_ conditions, specific C-terminally encoded peptides [CEPs; (*76*)] are induced in the root of *M. truncatula* (*77*). These CEPs are perceived in the shoot by the leucine-rich repeat receptor-like protein kinase (LRR-RLK) Compact Root Architecture 2 (*78*), which, in turn, promotes AM colonization (*77*). Conversely, under high P_i_ conditions or during AM colonization, distinct CLAVAT3/Endosperm Surrounding Region–related [CLE; (*79*)] peptides are expressed in the root and perceived in the shoot by the LRR-RLK Super Numeric Nodules (*80*, *81*), leading to suppression of AM colonization by modulating strigolactone signaling (*82*, *83*). It has been demonstrated in *M. truncatula* that CLEs can also act as positive regulators of AM. MtCLE16, which is sensed by the pseudokinase CORYNE [CRN; (*84*)], attenuates plant stress signaling, thereby enhancing arbuscule life span and abundance (*85*). Notably, several AM fungi also express CLE peptides, and RiCLE1, a MtCLE16-like peptide, promotes AM fungal entry (*86*) and increases AM fungal colonization via MtCRN (*85*). While our data support a systemic role of VIH2 in regulating AM colonization, they do not yet reveal whether this involves cross-talk between PP-InsP signaling and the AOM pathway or whether it operates through an independent systemic signaling route, representing an important question for future work.

Previous work has demonstrated that the SPX-PHR signaling module provides a molecular framework linking plant P_i_ status to both PSR and AM regulation. For example, strong ectopic expression of *PHR* in rice, *L. japonicus* (*Ubiquitin* promoter), and *M. truncatula* (35*S* promoter) has been shown to promote AM colonization (*11*, *42*, *43*) and improve yield (*11*). However, in *M. truncatula*, strong ectopic expression of *PHR2* controlled by the *Ubiquitin* promoter resulted in reduced AM development and increased the number of degrading arbuscules (*43*). Although effective, such strategies rely on genetically modified organisms, which are subject to strict regulatory oversight in many countries. By contrast, our approach could use genome editing to deactivate *VIH* genes, a method that many countries already regulate less stringently or are likely to in the near future (*87*). Genome editing could, in theory, also be applied to inactivate transcriptional SPX repressors to enhance PSR. However, the genetic evidence reveals a more complex picture. For example, *spx1 spx2 spx3 spx5* quadruple mutants in rice and a *spx1* single mutant in tomato display increased PSR and mycorrhization, while ectopic overexpression of *SPX1* in tomato or of *SPX1* and *SPX2* decreased arbuscule frequency and overall colonization (*41*, *42*). By contrast, a *spx1 spx3* double mutant in *M. truncatula* exhibited decreased AM colonization, while ectopic overexpression of *SPX1* or *SPX3* resulted not only in an increase in mycorrhization but also in enhanced arbuscule degradation. Moreover, expression patterns differ across species: *PHR2* is induced in arbuscule-containing cells in rice, but not in *M. truncatula* (*42*, *43*), whereas *SPX* genes are induced in arbuscule-containing cells in *M. truncatula* and tomato, but not in rice (*11*, *41*, *42*), suggesting that the SPX-PHR module is wired differently in different plant species (*10*). Our findings extend this framework by identifying VIH2-dependent InsP_8_ as a critical component that modulates AM colonization. However, VIH2-dependent defects affect not only InsP_8_ but also 1/3-InsP_7_ in a similar manner (Fig. 3), raising the possibility that 1/3-InsP_7_ may also contribute to the regulation of AM symbiosis. In this context, previous work has shown that alterations in specific InsP_7_ isomers can influence PSR signaling, for example, in *Arabidopsis itpk1* mutants, reduced InsP_8_ levels are accompanied by increased 5-InsP_7_, which can partially compensate for the loss of InsP_8_ in PSR regulation (*24*). Together with our observation that 1/3-InsP_7_ levels are altered in *vih2* plants, this highlights the possibility that multiple InsP_7_ isomers contribute to the regulation of P_i_ signaling and symbiotic responses, an aspect that will require further mechanistic investigation.

We propose that VIH2-dependent InsP_8_ modulates AM colonization, at least, in part, through the activity of PHR transcription factors, which regulate AM-associated transcriptional programs and selected AM marker genes (*11*, *42*, *43*). Future genetic interaction studies will clarify the extent to which InsP_8_ and/or 1/3-InsP_7_ regulation converges on PHR-mediated transcriptional control. PP-InsPs thereby add a further regulatory layer to the integration of P_i_ availability with symbiotic interactions, highlighting their role as central signaling molecules at the PSR-AM interface. Our approach not only complements but also broadens previous strategies to enhance AM colonization by targeting P sensing mechanisms.

In contrast to earlier approaches, our strategy targets signaling molecules like InsP_8_ or 5-InsP_7_, providing a more nuanced and adaptable approach, which enables cells to still adjust in response to ATP availability, P_i_ status, and environmental conditions (Fig. 3) (*24*). Such fine-tuning is not easily achievable in *spx* knockout or *PHR/SPX* overexpression lines, which induce permanent changes in specific regulatory pathways. Notably, the percentage of degrading arbuscules was significantly reduced in the *vih2*-*1* mutant (Fig. 5), and *vih2* mutants accumulated significantly more nutrients when colonized by AM fungi and grown in sand or field-collected topsoil (Figs. 4 and 6). This suggests that, in contrast to previous reports of enhanced AM fungal colonization accompanied by increased arbuscule degradation in *M. truncatula* SPX overexpression lines (*43*), targeted manipulation of PP-InsP levels via VIH2 promotes nutrient uptake and AM colonization without compromising arbuscule maintenance. Furthermore, the finding that local inactivation of VIH2 in the root is sufficient to enhance AM colonization opens avenues to reduce potential trade-offs associated with manipulating VIH2-dependent PP-InsPs across the entire plant (*26*, *88*). Specifically, ectopic expression of 5-β-phosphate-specific PP-InsP pyrophosphatases, such as Plant and Fungi Atypical Dual Specificity Phosphatases (PFA-DSPs) (*89*, *90*), in root tissues associated with AM colonization might provide a promising direction to calibrate this interaction. This approach would likely reduce unintended consequences caused by altered PP-InsP levels in other plant tissues. Alternatively, NUDIX-type PP-InsP hydrolases have recently emerged as additional enzymatic regulators of PP-InsP turnover (*90*–*93*). However, these enzymes exhibit diverse and subclade-specific substrate preferences, often showing only limited activity toward 5-β-phosphates, suggesting a broader and more complex functional role that extends beyond canonical Pi signaling control (*93*). Although *vih2* mutants showed increased AM colonization and elevated shoot nutrient levels, this did not result in increased shoot biomass under the conditions tested here, and effects on yield were not assessed.

Enhanced AM colonization offers a wide range of agronomic and ecological benefits (*33*, *94*). Beyond improved P acquisition and reduced reliance on mineral P fertilizers, AM fungi can limit the accumulation of toxic contaminants such as cadmium and uranium in agricultural soils (*95*). In line with this broader context, our data show that *vih2* mutants accumulate increased levels of multiple nutrients when colonized by AM fungi, both under controlled and field soil conditions (Figs. 4E and 6 and table S3). Furthermore, AM fungi can improve plant drought tolerance due to the extension of the effective root length by hyphal networks and by improving soil water conductance (*96*), something that crops with enhanced AM-colonization might benefit from. Last, AM substantially contributes to global carbon cycles (e.g., ~4 billion tonne CO_2_ equivalents annually transferred from plants to AM mycelium) (*97*), underscoring the critical role of AM in global climate regulation and the potential benefits integrating AM into future-oriented agricultural practices might offer.

While these advantages are compelling, the agronomic outcome of increased AM colonization is likely to be context dependent, and potential trade-offs such as additional carbon costs should not be overlooked. Increased nutrient uptake per se does not necessarily lead to improved nutrient-use efficiency or yield and may be counterproductive in some contexts if enhanced uptake is not coupled to biomass production or beneficial nutrient allocation. Our approach provides a means both to stimulate AM colonization and to quantify such costs under realistic field conditions and in relevant crops, helping to identify scenarios where the benefits outweigh possible drawbacks. By modulating PP-InsPs, our strategy provides a conceptual framework to examine how AM colonization influences crop resilience and nutrient uptake while allowing the fine-tuning needed for different agricultural settings.

## MATERIALS AND METHODS

### Molecular cloning, constructs, and primers

A detailed description of the constructs used in this study is provided in table S1. A list of primers can be found in table S2. The open reading frames (ORFs) of both (short and long) versions of the *VIH2* kinase domain were amplified from *L. japonicus* seedling cDNA by polymerase chain reaction (PCR), fused to Gateway-compatible *attB*1 and *att*B2 recombination sites and cloned in pDONR221 (Invitrogen) with BP clonase II (Invitrogen). For the expression of recombinant fusion proteins with an N-terminal His_6_–maltose-binding protein (MBP) epitope tag, the ORFs were recombined into the pDest-566 bacterial expression vector (Addgene plasmid no. 11517 was a gift from D. Esposito; www.addgene.org/11517/; RRID: Addgene_11517) by LR clonase II (Invitrogen). For yeast experiments, constructs harboring the *VIH2* kinase domains translationally fused to a C-terminal V5 epitope tag were used. The ORFs harboring the *V5* sequence and Gateway-compatible *attB*1 and *att*B2 recombination sites were cloned with BP clonase II in pDONR221 and then recombined into the destination vector pAG426GPD-*ccdB* (*98*) (Addgene plasmid no. 14156 was a gift from S. Lindquist; www.addgene.org/14156/; RRID:Addgene_14156) with LR clonase II.

### Plant material

*L. japonicus* ecotype Gifu B-129 wild-type and two LORE1 mutant lines (*62*), 30002777 (*vih2*-*1*) and 30058924 (*vih2*-*2*), homozygous for a retrotransposon insertion in *L. japonicus VIH2* were used in this study.

### *L. japonicus* seed sterilization, hydration, stratification, and germination

*L. japonicus* seeds were scarified, surface sterilized with sterilization solution (1% sodium hypochlorite and 0.1% SDS) for 10 min, rinsed five times with autoclaved Milli-Q water, and soaked in autoclaved Milli-Q water with end-over-end mixing either for 16 hours at 4°C or for 4 hours at room temperature. Subsequently, seeds were stratified on 0.8% (w/v) plant agar (P1001; Duchefa Biochemie) in square plates (120 mm by 120 mm by 15 mm) for 3 days at 4°C in the dark. Plates were transferred to a phytochamber, and seeds were germinated for 3 days at 22°C in the dark.

For SAX-HPLC analyses, *L. japonicus* seeds were scarified and surface sterilized with 1.2% sodium hypochlorite for 3 min followed by 70% ethanol for 3 min and then 100% ethanol before transfer to filter paper for drying. Subsequently, seeds were stratified on 0.8% (w/v) bacteriological agar in square plates (120 mm by 120 mm by 15 mm) for 3 days at 4°C in the dark before transfer to short day conditions (8-hour light at 22°C and 16-hour dark at 20°C).

### *L. japonicus* inoculation with AM fungal spores

After germination, *L. japonicus* seedlings were cultivated for 7 days at 22°C in long-day conditions (16-hour light and 8-hour dark; 160 μmol m^−2^ s^−1^). Five 10-day-old seedlings were transplanted into one pot (9 cm by 9 cm by 9.5 cm) containing 300 ml of washed and autoclaved sand, and each plant was inoculated with 500 spores of *R. irregularis* SYMPLANTA-001 research grade (Symplanta, 00101SP). Plants were fertilized once a week with liquid *Lotus* cultivation medium [25, 250, 750, 1500, or 2500 μM KH_2_PO_4_; 0, 1000, 1750, 2250, or 2475 μM KCl; 5 mM KNO_3_; 50 μM FeNaEDTA; 1 μM MnSO_4_; 0.5 μM ZnSO_4_; 0.2 μM CuSO_4_; 2 μM H_3_BO_4_; 0.1 μM Na_2_MoO_4_; 0.1 μM CoSO_4_; 250 μM MgSO_4_; 1 mM CaCl_2_; and 2 mM MES (pH 6.1)] and grown at 22°C in long-day conditions (16-hour light and 8-hour dark; 160 μmol m^−2^ s^−1^). Shoots and roots were harvested at 20 to 35 days postinoculation.

### Cultivation of *L. japonicus* in field-collected topsoil with moderate nitrogen, phosphorus, and potassium availabilities

Topsoil with moderate nitrogen, phosphorus, and potassium availabilities was collected from a field site on the University of Bonn campus Poppelsdorf (Carl-Troll-Str. 8–10) with unknown management history but previously used for grazing goats. Freshly excavated soil, stored at 4°C, was extracted with 1% K_2_SO_4_ soon after sampling for determination of mineral N (nitrate, ammonium) using an autoanalyzer (SEAL Analytical AA3). Air-dried, mechanically broken up, and sieved (<2 mm) soil was analyzed for available phosphorus and potassium by the Calcium-Acetate-Lactate (CAL) extraction (Schüller) with buffered calcium lactate–acetate solution (pH 4.1); phosphorus was measured colorimetrically (ammonium molybdate method), and potassium was measured by flame atomic emission spectrometry (Eppendorf ELEX 6361). Micronutrients (B, Mn, and Cu) were extracted with CaCl_2_-DTPA (CAT) solution (following the guidlines of the Verband deutscher landwirtschaftlicher Untersuchungs- und Forschungsanstalten e. V. ) and measured by inductively coupled plasma optical emission spectroscopy (ICP-OES; iCAP PRO X Duo, Thermo Fisher Scientific). Total C, N, and S were determined by elemental analysis (EA 3000, HEKAtech). Soil pH was measured in 0.01 M CaCl_2_ solution (following the VDLUFA guidelines).

After germination, *L. japonicus* seedlings were cultivated for 7 days at 22°C in long-day conditions (16-hour light and 8-hour dark; 160 μmol m^−2^ s^−1^). Five 10-day-old seedlings were transplanted into one pot (9 cm by 9 cm by 9.5 cm) containing 300 ml of field-collected topsoil. Plants were fertilized once in the first week with liquid *Lotus* cultivation medium [25 μM KH_2_PO_4_; 0, 1000, 1750, 2250, or 2475 μM KCl; 5 mM KNO_3_; 50 μM FeNaEDTA; 1 μM MnSO_4_; 0.5 μM ZnSO_4_; 0.2 μM CuSO_4_; 2 μM H_3_BO_4_; 0.1 μM Na_2_MoO_4_; 0.1 μM CoSO_4_; 250 μM MgSO_4_; 1 mM CaCl_2_; and 2 mM MES (pH 6.1)] and subsequently once a week with Milli-Q water and grown at 22°C in long-day conditions (16-hour light and 8-hour dark; 160 μmol m^−2^ s^−1^). Shoots and roots were harvested at 5.5 weeks postinoculation.

### Ink-vinegar staining and AM quantification

The protocol for ink-vinegar staining of colonized *L. japonicus* roots was adapted from Vierheilig *et al.* (*99*). In brief, root systems with the exception of the uppermost and lowermost 1 cm were harvested, washed carefully to remove sand, and placed in 2-ml reaction tubes containing 1.5 ml of 10% KOH. Roots were cleared for 12 hours at 60°C. KOH was removed, and roots were rinsed once with autoclaved Milli-Q water and twice with 10% acetic acid. Roots were covered with 5% ink-vinegar staining solution (4001; Pelikan) and incubated at 95°C for 5 min. Roots were then rinsed once with autoclaved Milli-Q water and once with 5% acetic acid. Last, roots were destained in 5% acetic acid for 20 min at room temperature, 5% acetic acid was replaced with 50% glycerol, and roots were stored at 4°C until use.

The protocol for the quantification of AM structures was adapted from McGonigle *et al.* (*100*). In brief, ink-vinegar–stained roots were cut into 1.5-cm-long pieces and for each root system a random selection of 15 root pieces was mounted on a microscope slide and stored at 4°C until use. Stained roots were analyzed using a Zeiss Axioplan equipped with an Axiocam MRc5 digital camera with ×20 magnification. The presence or absence of AM fungal structures (arbuscules, hyphae, and vesicles) was scored in ten intersections per root piece resulting in a total of 150 observations per root system. The percentage of a specific AM fungal structure is calculated by dividing the sum of observations in all intersections by the number of intersections (e.g., 75 arbuscules per 150 intersections equals 50%).

### WGA staining and microscopic analysis

The protocol for WGA staining of colonized *L. japonicus* roots was adapted from Dreher *et al.* (*101*). In brief, root systems with the exception of the uppermost and lowermost 1 cm were harvested and placed in 2-ml reaction tubes containing 1.5 ml of 50% (v/v) ethanol for 4 hours at room temperature. Roots were then incubated in 20% KOH for 10 min at 90°C, rinsed once with autoclaved Milli-Q water, and incubated in 0.1 M HCl for 2 hours at room temperature. Subsequently, roots were transferred to WGA–Alexa Fluor 488 staining solution [WGA–Alexa Fluor 488, 0.2 μg ml^−1^ (W11261, Thermo Fisher Scientific) in phosphate-buffered saline, 135 mM NaCl, 3 mM KCl, 1.5 mM KH_2_PO_4_, and 8 mM Na_2_HPO_4_ (pH 7.4)] and incubated for a minimum of 4 days at room temperature. For analysis and quantification, root systems were cut into 1.5-cm-long pieces, and, for each root system, a random selection of 15 root pieces was mounted on a microscope slide. Stained roots were visualized using a Zeiss LSM 900 confocal microscope with ×20 or ×40 magnification. The presence or absence of AM fungal structures (arbuscules, hyphae, and vesicles) was scored in 10 intersections per root piece resulting in a total of 150 observations per root system. The percentage of a specific AM fungal structure is calculated by dividing the sum of observations in all intersections by the number of intersections (e.g., 75 arbuscules per 150 intersections equals 50%). In addition, the overall number of arbuscules per intersection was assessed, and the length of each arbuscule within a scored intersection was measured using the Zeiss ZEN Black software. The presence or absence of septa was investigated for each scored arbuscule. Representative WGA images were selected from these randomly sampled root segments.

### *L. japonicus* grafting

The protocol for grafting of *L. japonicus* was adapted from Sexauer *et al.* (*102*). In brief, *L. japonicus* seeds were sterilized, hydrated, and stratified as described above. For germination, plates were incubated horizontally upside down in a phytochamber for 3 days at 24°C in the dark. After germination, plants were transferred to square plates (120 mm by 120 mm by 15 mm) containing *Lotus* cultivation medium supplemented with 750 μM KH_2_PO_4_ and 1% Noble-Agar (J10907.A1, Thermo Fisher Scientific) and cultivated vertically for 3 days at 21°C in long-day conditions (16-hour light and 8-hour dark; 140 μmol m^−2^ s^−1^). Seedlings were then cut in the middle of the hypocotyl, and shoots were transplanted on root stocks to generate self-grafts and reciprocal grafts between the wild type and the *vih2*-*1* mutant. To arrest the fresh grafts, shoots and roots were inserted in sterile silicone tubings (0.76 mm, VWR). Grafts were cultivated on square plates (120 mm by 120 mm by 15 mm) containing *Lotus* cultivation medium supplemented with 25 or 750 μM KH_2_PO_4_ and 1% Noble-Agar (J10907.A1, Thermo Fisher Scientific) for 7 days at 24°C in long-day conditions (16-hour light and 8-hour dark; 160 μmol m^−2^ s^−1^) and then subjected to inoculation with AM fungal spores as described above.

### *L. japonicus* hydroponic cultivation

*L. japonicus* seeds were sterilized and hydrated as described above. Seeds were germinated in cut-open 1.5-ml reaction tubes filled with rockwool and inserted into flat black boxes (bikapak; Logiflex, lid black, 00202201; Logiflex, 660 ml, box black, 0202233) filled with 250 ml of liquid *Lotus* cultivation medium containing sufficient P_i_ [+P_i_ liquid medium; 1500 μM KH_2_PO_4_, 5 mM KNO_3_, 50 μM FeNaEDTA, 1 μM MnSO_4_, 0.5 μM ZnSO_4_, 0.2 μM CuSO_4_, 2 μM H_3_BO_4_, 0.1 μM Na_2_MoO_4_, 0.1 μM CoSO_4_, 250 μM MgSO_4_, 1 mM CaCl_2_, and 2 mM MES (pH 6.1)] for 3 days at 22°C in the dark. Seedlings were further grown in the flat boxes for 17 days, and the nutrient solution was exchanged weekly. Plants were then transferred to black 2.5-liter buckets (bikapak; round bucket, 2.5 liters, black, 00201989; lid for round bucket, 2.5 liters, black, 00201899) containing 1.8 liters of +P_i_ liquid medium and grown for 7 days. The nutrient solution was exchanged every second day. An air pump (OSAGA, MK-9502) was connected to the system to ensure continuous aeration. Subsequently, plants were transferred to buckets containing P_i_-free liquid *Lotus* cultivation medium [−P_i_ liquid medium; 1500 μM KCl, 5 mM KNO_3_, 50 μM FeNaEDTA, 1 μM MnSO_4_, 0.5 μM ZnSO_4_, 0.2 μM CuSO_4_, 2 μM H_3_BO_4_, 0.1 μM Na_2_MoO_4_, 0.1 μM CoSO_4_, 250 μM MgSO_4_, 1 mM CaCl_2_, and 2 mM MES (pH 6.1)] and starved for P_i_ for 10 days. Following P_i_ starvation, plants were transferred back to +P_i_ liquid medium for P_i_ resupply for 0.5 to 96 hours. Plants were maintained at 22°C in long-day conditions (16-hour light and 8-hour dark; 160 μmol m^−2^ s^−1^) throughout the experiment. Shoots and roots were harvested from plants grown in +P_i_ liquid medium, from P_i_ starved plants, and from plants resupplied with P_i_ for the indicated time.

### RNA extraction and cDNA synthesis

Total RNA was extracted from *L. japonicus* tissue using the innuPREP Plant RNA kit (Innuscreen GmbH) according to the manufacturer’s instructions. Lysis Solution PL was applied for root material. The optional deoxyribonuclease (DNase) I on-column treatment using the innuPREP DNase I Digest kit (Innuscreen GmbH) was performed according to the manufacturer’s instructions to ensure removal of genomic DNA. RNA was eluted in 35 μl of ribonuclease-free water, and concentration was measured with a spectrophotometer. The absence of genomic DNA was confirmed by PCR. First-strand cDNA was synthesized in 20-μl reactions from 500 ng of total RNA with the RevertAid First Strand cDNA Synthesis kit (Thermo Fisher Scientific) according to the manufacturer’s instructions using oligo(dT)_18_ primers. For subsequent gene expression analysis, cDNA was diluted 1:10 in autoclaved Milli-Q water.

### Gene expression analysis

Quantitative reverse transcription (qRT)–PCR was performed using the PowerUP SYBR Green Master Mix for qPCR (Applied Biosystems) according to the manufacturer’s instructions for a final volume of 10 μl containing 15 ng of cDNA and 400 nM of each primer. The reactions were carried out in a QuantStudio 5 real-time PCR system (Applied Biosystems) following the manufacturer’s instructions for the fast-cycling mode. PCR program: 50°C for 2 min, 95°C for 2 min, 40× (95°C for 1 s and 60°C for 30 s), 95°C for 10 s, and a melt curve from 60° to 95°C, with increment of 0.5°C per 5 s. Expression was normalized to the reference gene *Ubiquitin*. Three to five biological replicates per sample were analyzed in technical duplicates or triplicates. A primer list can be found in table S2.

### Yeast strains, transformation, growth, and complementation (spotting assay)

The BY4741 wild type (MATa *his*3Δ *leu*2Δ *met*15Δ *ura*3Δ) and *vip1*Δ (MATa *his*3Δ *leu*2Δ *met*15Δ *ura*3Δ *vip1*Δ*::KanMX*) were obtained from Euroscarf. The yeast cells were transformed with the Li-acetate method (*103*) and grown on 2× CSM-Ura plates for 3 days at 28°C. Yeast transformants were grown in the respective liquid medium overnight at 28°C in a rotating wheel before radioactive labeling with [^3^H]-*myo*-inositol [American Radiolabeled Chemicals (ARC)] or performing growth complementation assays. For the latter, optical density at 600 nm from the overnight cultures was measured, and the values adjusted to 1.0. Then, an eightfold serial dilution was prepared in a 96-well plate, and, subsequently, 10 μl of each dilution was spotted on selective solid medium (*104*) with or without 2 mM 6-azauracil (Sigma-Aldrich, A1757) dissolved in water. After 3 days incubation at 28°C, pictures were taken with a ChemiDoc MP imager (Bio-Rad) using white backlight.

### Radiolabeling of transformed yeast and of *L. japonicus* and SAX-HPLC analysis

Transformed yeast was radiolabeled with [^3^H]-*myo*-inositol (ARC) and extracted as published previously (*26*, *105*). *L. japonicus* seedlings were radiolabeled, extracted, and analyzed by SAX-HPLC as described before (*63*).

### Yeast protein extraction and immunodetection

Yeast proteins were extracted (*89*), and LjVIH2^KD^ was visualized via immunodetection with a primary mouse anti-V5 antibody (Invitrogen, 1:2000 dilution) followed by a secondary Alexa Fluor Plus 680 goat anti-mouse antibody (Invitrogen, 1:10 000 dilution). A rabbit anti-Gal4 antibody (Santa Cruz Biotechnology, 1:1000 dilution) followed by a secondary Alexa Fluor Plus 800 goat anti-rabbit antibody (Invitrogen, 1:10 000 dilution) was used as loading control. The fluorescent signals were detected with a ChemiDoc MP imager (Bio-Rad).

### Generation of recombinant proteins and in vitro kinase assays

His_6_-MBP–fused recombinant LjVIH2 protein and free His_8_-MBP protein (*26*) were expressed in *E. coli* BL21-CodonPlus(DE3)-RIL cells (Stratagene). The cells were grown in LB medium overnight at 37°C and diluted 1:500 in 2x Yeast Extract Tryptone medium the next morning. The cultures were grown at 37°C for 3 hours until protein expression was induced with 100 μM isopropyl-β-d-thiogalactopyranoside. They were then transferred to 12°C, and, after 3 days, the cells were pelleted and resuspended in 15 ml of lysis buffer [20 mM Na_2_HPO_4_, 300 mM NaCl, 2 mM dithiothreitol (DTT), 0.05 mM EDTA, and 1% (v/v) Triton X-100 (pH 7.4)]. Glass beads (5 ml; 0.1-mm diameter) were added, and the tubes vortexed eight times for 1 min, with 1 min on ice between each vortexing step. The samples were centrifuged at 15,600*g* at 4°C for 20 min, and the supernatant transferred to a fresh 50-ml tube and centrifuged again for 20 min. The supernatant was transferred to 400 μl of Ni–nitrilotriacetic acid (NTA) agarose beads (Macherey-Nagel), which had previously been washed once with ddH_2_O and twice with lysis buffer. The samples were incubated overnight in an overhead shaker at 4°C. Next, the samples were centrifuged at 800*g* at 4°C for 1 min, the supernatant was discarded, and the beads were washed three times with binding buffer [20 mM Na_2_HPO_4_, 500 mM NaCl, and 25 mM imidazole (pH 7.4)]. For elution of the protein, the Ni-NTA resin was incubated three times with 250 μl of elution buffer [20 mM Na_2_HPO_4_, 500 mM NaCl, and 250 mM imidazole (pH 7.4)] for 5 min, and the eluates were collected after centrifugation at 800*g* at 4°C for 1 min. The proteins were desalted with the use of Amicon 50-kDa cutoff centricons (Merck) following the manufacturer’s protocol. For protein storage, 20% glycerol was added. Protein concentrations were estimated by comparison to bovine serum albumin (BSA) standard amounts on a Coomassie blue–stained SDS-PAGE.

Kinase reactions were performed by incubating 0.2 to 100 μM LjVIH2^sKD^ with 0.33 mM InsP_7_ in 20 mM Hepes (pH 7.5), 5 mM MgCl_2_, 1 mM DTT, and 12.5 mM ATP for 1 hour at 22°C. After incubation, InsPs and PP-InsPs were visualized by PAGE (*106*). Extracts of *Arabidopsis mrp5* seed, which accumulate high amounts of InsP_7_ and InsP_8_ (*27*), were purified by TiO_2_ pull-down and used as a marker for InsP_6_, InsP_7_, and InsP_8_ (*107*).

### NMR spectroscopy analysis of in vitro kinase assays

LjVIH2 kinase assays were carried out in buffered solutions in D_2_O containing 20 mM Hepes (pH 7.5), 150 mM NaCl, BSA (0.2 mg/ml), 1 mM DTT, 0.1% (m/v) CHAPS, 5 mM MgCl_2_, 0.5 mM ATP, 5 mM creatine phosphate, and creatine kinase (1 U/ml; from rabbit muscle) at a total volume of 150 μl. LjVIH2 (2 μM) was preincubated in the respective reaction buffer at 22°C, and the reaction was started by adding [^13^C_6_]5-InsP_7_ to yield a final concentration of 100 μM. Reactions were incubated for 7 hours at 22°C and were quenched by the addition of 400 μl of a quenching solution [20 mM EDTA (pH 6.0) and 68.75 mM NaCl]. For reactions containing hPPIP5K2^KD^, 200 nM SUMO-PPIP5K2^KD^ was preincubated at 37°C in a buffered solution containing 20 mM MES (pH 6.0), 150 mM NaCl, BSA (0.2 mg/ml), 1 mM DTT, 0.1% (v/v) CHAPS, 5 mM MgCl_2_, 0.5 mM ATP, 5 mM creatine phosphate, and creatine kinase (1 U/ml; from rabbit muscle) at a total volume of 150 μl. The reaction was started by adding [^13^C_6_]5-InsP_7_ to yield a final concentration of 100 μM. After overnight treatment at 37°C, reactions were quenched by the addition of 400 μl of a quenching solution [20 mM EDTA (pH 6.0) and 68.75 mM NaCl]. LjVIH2 was incubated for 7 hours, allowing simultaneous detection of starting material and product, whereas hPPIP5K2 was incubated overnight to ensure complete conversion to 1/3,5-InsP8.

Quenched samples were centrifuged at 15,000*g* for 5 min, and the supernatant was analyzed by NMR spectroscopy. Samples were measured as previously described (*108*): NMR spectra were recorded on an AVIII 600 spectrometer (600 MHz for ^1^H-, 151 MHz for ^13^C-, and 61 MHz for ^15^N-measurements) with a cryogenically cooled CQI-cryoprobe from Bruker Corp. The pulse sequence for BIRD-{^1^H,^13^C}HMQC used for measuring was based on the hmqcbiph pulse program from Bruker Corp. 2D-BIRD-{^1^H,^13^C}HMQC measurements were carried out with TD(^13^C) = 256 and 64 scans at a temperature of 310 K. Analysis of NMR spectra was performed with TopSpin 3.5 from Bruker Corp.

### Purification of inositol phosphates from *L. japonicus*, PAGE of inositol phosphates, and CE-ESI-MS analysis

Inositol phosphates were extracted with TiO_2_ or Nb_2_O_5_ as indicated and purified as described previously (*109*). Purified inositol phosphates were analyzed by PAGE as a purely qualitative assessment (*n* = 1 to 2) and then quantified by CE-ESI-MS (*n* = 4 to 5) (*24*, *59*, *61*). Enantiomers are not resolved. For InsP_4_, the existence of undefined isomers cannot be excluded.

### Measurement of shoot nutrient content

For nutrient analysis, plant material was dried at 60°C and ground to powder in a bead mill using ZrO_2_ beads, with agate beads included in most cases. Approximately 5 mg of the powdered material was analyzed for carbon (C) and nitrogen (N) content using an elemental analyzer (EuroEA 3000, HEKAtech). For the determination of all other reported nutrient concentrations, acid digestions with ~50 mg of dried plant material and 2.5 to 3.5 ml of concentrated HNO_3_ were performed in a microwave-accelerated reaction system (Mars6, CEM Corporation) followed by analysis with ICP-OES (iCAP PRO X Duo, Thermo Fisher Scientific).

### BLAST search and phylogenetic analysis

A protein BLAST search was conducted using the amino acid sequence of the *S. cerevisiae* Vip1 ATP-grasp kinase domain (amino acid residues 1 to 515) as a query. Tools that were used for protein BLAST search are listed in table S4. The ATP-grasp kinase domains of the sequences retrieved were annotated using InterPro [www.ebi.ac.uk/interpro/; (*110*)]. Subsequently, the annotated ATP-grasp kinase domains were aligned using the MUSCLE 5.1 algorithm (*111*). The aligned sequences were then used to construct a maximum-likelihood phylogenetic tree using IQ-Tree (*112*), with a bootstrap support of 1000 replicates. Bootstrap values below 20% were omitted. The resulting phylogenetic tree was visualized using iTOL [https://itol.embl.de/ (*113*)].

### Statistical analysis and data visualization

Statistical analysis and data visualization were performed in GraphPad Prism (version 10.4.2 for Windows, GraphPad Software, Boston, Massachusetts, USA; www.graphpad.com) and Affinity Designer (version 2.6.0 for Windows; https://affinity.serif.com/de/designer/). Boxplots together with individual data points were used to display data in Figs. 2C, 3 (D to F), 4A, 5A, 6 (A to E), and 7 (A and C) and figs. S6 to S8, S11, S12, S15A, S16 (A and B), S17 (A and D), and S18 (black line, median; box, 25th to 75th percentiles; whiskers, 10th to 90th percentiles). Individual data points only were plotted in Figs. 2A, 4C, 5B, 6F, and 7E and figs. S4, S13A, S14, S16D, and S17 (C and F). Statistical tests applied are stated in the figure legends. All experiments have been performed independently between one and four times with similar results.

## Supplementary Materials

### This PDF file includes:

Figs. S1 to S18 Tables S1 to S4 References

## References

[1] J. Paz-Ares, M. I. Puga, M. Rojas-Triana, I. Martinez-Hevia, S. Diaz, C. Poza-Carrión, M. Miñambres, A. Leyva, Plant adaptation to low phosphorus availability: Core signaling, crosstalks, and applied implications. Mol. Plant 15, 104–124 (2022). 34954444 10.1016/j.molp.2021.12.005

[2] C. L. Dybas, Dead zones spreading in world oceans. Bioscience 55, 552–557 (2005).

[3] V. Rubio, F. Linhares, R. Solano, A. C. Martín, J. Iglesias, A. Leyva, J. Paz-Ares, A conserved MYB transcription factor involved in phosphate starvation signaling both in vascular plants and in unicellular algae. Genes Dev. 15, 2122–2133 (2001). 11511543 10.1101/gad.204401 PMC312755

[4] R. Bustos, G. Castrillo, F. Linhares, M. I. Puga, V. Rubio, J. Pérez-Pérez, R. Solano, A. Leyva, J. Paz-Ares, A central regulatory system largely controls transcriptional activation and repression responses to phosphate starvation in *Arabidopsis*. PLOS Genet. 6, e1001102 (2010). 20838596 10.1371/journal.pgen.1001102 PMC2936532

[5] L. Sun, L. Song, Y. Zhang, Z. Zheng, D. Liu, Arabidopsis PHL2 and PHR1 act redundantly as the key components of the central regulatory System controlling transcriptional responses to phosphate starvation. Plant Physiol. 170, 499–514 (2016). 26586833 10.1104/pp.15.01336 PMC4704584

[6] Z. Wang, Z. Zheng, L. Song, D. Liu, Functional characterization of Arabidopsis PHL4 in plant response to phosphate starvation. Front. Plant Sci. 9, 1432 (2018). 30327661 10.3389/fpls.2018.01432 PMC6174329

[7] F. Ren, Q.-Q. Guo, L.-L. Chang, L. Chen, C.-Z. Zhao, H. Zhong, X.-B. Li, Brassica napus PHR1 gene encoding a MYB-like protein functions in response to phosphate starvation. PLOS ONE 7, e44005 (2012). 22952851 10.1371/journal.pone.0044005 PMC3430610

[8] J. Zhou, F. Jiao, Z. Wu, Y. Li, X. Wang, X. He, W. Zhong, P. Wu, OsPHR2 is involved in phosphate-starvation signaling and excessive phosphate accumulation in shoots of plants. Plant Physiol. 146, 1673–1686 (2008). 18263782 10.1104/pp.107.111443 PMC2287342

[9] J. Wang, J. Sun, J. Miao, J. Guo, Z. Shi, M. He, Y. Chen, X. Zhao, B. Li, F. Han, Y. Tong, Z. Li, A phosphate starvation response regulator Ta-PHR1 is involved in phosphate signalling and increases grain yield in wheat. Ann. Bot. 111, 1139–1153 (2013). 23589634 10.1093/aob/mct080 PMC3662521

[10] P. Wang, R. Snijders, W. Kohlen, J. Liu, T. Bisseling, E. Limpens, Medicago SPX1 and SPX3 regulate phosphate homeostasis, mycorrhizal colonization, and arbuscule degradation. Plant Cell 33, 3470–3486 (2021). 34469578 10.1093/plcell/koab206 PMC8567062

[11] D. Das, M. Paries, K. Hobecker, M. Gigl, C. Dawid, H.-M. Lam, J. Zhang, M. Chen, C. Gutjahr, PHOSPHATE STARVATION RESPONSE transcription factors enable arbuscular mycorrhiza symbiosis. Nat. Commun. 13, 477 (2022). 35078978 10.1038/s41467-022-27976-8 PMC8789775

[12] Q. Lv, Y. Zhong, Y. Wang, Z. Wang, L. Zhang, J. Shi, Z. Wu, Y. Liu, C. Mao, K. Yi, P. Wu, SPX4 negatively regulates phosphate signaling and homeostasis through its interaction with PHR2 in rice. Plant Cell 26, 1586–1597 (2014). 24692424 10.1105/tpc.114.123208 PMC4036573

[13] M. I. Puga, I. Mateos, R. Charukesi, Z. Wang, J. M. Franco-Zorrilla, L. de Lorenzo, M. L. Irigoyen, S. Masiero, R. Bustos, J. Rodríguez, A. Leyva, V. Rubio, H. Sommer, J. Paz-Ares, SPX1 is a phosphate-dependent inhibitor of PHOSPHATE STARVATION RESPONSE 1 in *Arabidopsis*. Proc. Natl. Acad. Sci. U.S.A. 111, 14947–14952 (2014). 25271326 10.1073/pnas.1404654111 PMC4205628

[14] Z. Wang, W. Ruan, J. Shi, L. Zhang, D. Xiang, C. Yang, C. Li, Z. Wu, Y. Liu, Y. Yu, H. Shou, X. Mo, C. Mao, P. Wu, Rice SPX1 and SPX2 inhibit phosphate starvation responses through interacting with PHR2 in a phosphate-dependent manner. Proc. Natl. Acad. Sci. U.S.A. 111, 14953–14958 (2014). 25271318 10.1073/pnas.1404680111 PMC4205599

[15] W. Qi, I. W. Manfield, S. P. Muench, A. Baker, AtSPX1 affects the AtPHR1–DNA-binding equilibrium by binding monomeric AtPHR1 in solution. Biochem. J. 474, 3675–3687 (2017). 28887383 10.1042/BCJ20170522 PMC5651819

[16] Y. Zhong, Y. Wang, J. Guo, X. Zhu, J. Shi, Q. He, Y. Liu, Y. Wu, L. Zhang, Q. Lv, C. Mao, Rice SPX6 negatively regulates the phosphate starvation response through suppression of the transcription factor PHR2. New Phytol. 219, 135–148 (2018). 29658119 10.1111/nph.15155

[17] M. B. Osorio, S. Ng, O. Berkowitz, I. De Clercq, C. Mao, H. Shou, J. Whelan, R. Jost, SPX4 Acts on PHR1-dependent and -independent regulation of shoot phosphorus status in Arabidopsis. Plant Physiol. 181, 332–352 (2019). 31262954 10.1104/pp.18.00594 PMC6716250

[18] R. Wild, R. Gerasimaite, J.-Y. Jung, V. Truffault, I. Pavlovic, A. Schmidt, A. Saiardi, H. J. Jessen, Y. Poirier, M. Hothorn, A. Mayer, Control of eukaryotic phosphate homeostasis by inositol polyphosphate sensor domains. Science 352, 986–990 (2016). 27080106 10.1126/science.aad9858

[19] M. K. Ried, R. Wild, J. Zhu, J. Pipercevic, K. Sturm, L. Broger, R. K. Harmel, L. A. Abriata, L. A. Hothorn, D. Fiedler, S. Hiller, M. Hothorn, Inositol pyrophosphates promote the interaction of SPX domains with the coiled-coil motif of PHR transcription factors to regulate plant phosphate homeostasis. Nat. Commun. 12, 384 (2021). 33452263 10.1038/s41467-020-20681-4 PMC7810988

[20] J. Dong, G. Ma, L. Sui, M. Wei, V. Satheesh, R. Zhang, S. Ge, J. Li, T. E. Zhang, C. Wittwer, H. J. Jessen, H. Zhang, G. Y. An, D. Y. Chao, D. Liu, M. Lei, Inositol pyrophosphate InsP8 acts as an intracellular phosphate signal in *Arabidopsis*. Mol. Plant 12, 1463–1473 (2019). 31419530 10.1016/j.molp.2019.08.002

[21] V. Raboy, *Low phytic acid* crops: Observations based on four decades of research. Plants 9, 140 (2020). 31979164 10.3390/plants9020140 PMC7076677

[22] S. B. Shears, Inositol pyrophosphates: Why so many phosphates? Adv. Biol. Regul. 57, 203–216 (2015). 25453220 10.1016/j.jbior.2014.09.015 PMC4291286

[23] J. Zhu, K. Lau, R. Puschmann, R. K. Harmel, Y. Zhang, V. Pries, P. Gaugler, L. Broger, A. K. Dutta, H. J. Jessen, G. Schaaf, A. R. Fernie, L. A. Hothorn, D. Fiedler, M. Hothorn, Two bifunctional inositol pyrophosphate kinases/phosphatases control plant phosphate homeostasis. eLife 8, e43582 (2019). 31436531 10.7554/eLife.43582 PMC6731061

[24] E. Riemer, D. Qiu, D. Laha, R. K. Harmel, P. Gaugler, V. Gaugler, M. Frei, M.-R. Hajirezaei, N. P. Laha, L. Krusenbaum, R. Schneider, A. Saiardi, D. Fiedler, H. J. Jessen, G. Schaaf, R. F. H. Giehl, ITPK1 is an InsP6/ADP phosphotransferase that controls phosphate signaling in *Arabidopsis*. Mol. Plant 14, 1864–1880 (2021). 34274522 10.1016/j.molp.2021.07.011 PMC8573591

[25] C. Cridland, A. Russo, B. Craige, J. Donahue, X. M. Zhang, M. Payne, G. Gillaspy, C. Freed, Enhancing inositol pyrophosphate accumulation in plants alters growth, phosphate homeostasis, and insect herbivory. Plant J. 123, e70315 (2025). 40639431 10.1111/tpj.70315 PMC12245475

[26] D. Laha, P. Johnen, C. Azevedo, M. Dynowski, M. Weiß, S. Capolicchio, H. Mao, T. Iven, M. Steenbergen, M. Freyer, P. Gaugler, M. K. F. de Campos, N. Zheng, I. Feussner, H. J. Jessen, S. C. M. Van Wees, A. Saiardi, G. Schaaf, VIH2 regulates the synthesis of inositol pyrophosphate InsP_8_ and jasmonate-dependent defenses in *Arabidopsis*. Plant Cell 27, 1082–1097 (2015). 25901085 10.1105/tpc.114.135160 PMC4558690

[27] M. Desai, P. Rangarajan, J. L. Donahue, S. P. Williams, E. S. Land, M. K. Mandal, B. Q. Phillippy, I. Y. Perera, V. Raboy, G. E. Gillaspy, Two inositol hexakisphosphate kinases drive inositol pyrophosphate synthesis in plants. Plant J. 80, 642–653 (2014). 25231822 10.1111/tpj.12669

[28] H. Wang, V. S. Nair, A. A. Holland, S. Capolicchio, H. J. Jessen, M. K. Johnson, S. B. Shears, Asp1 from Schizosaccharomyces pombe binds a [2Fe-2S](2+) cluster which inhibits inositol pyrophosphate 1-phosphatase activity. Biochemistry 54, 6462–6474 (2015). 26422458 10.1021/acs.biochem.5b00532 PMC4641441

[29] M. Pascual-Ortiz, A. Saiardi, E. Walla, V. Jakopec, N. A. Künzel, I. Span, A. Vangala, U. Fleig, Asp1 bifunctional activity modulates spindle function via controlling cellular inositol pyrophosphate levels in *Schizosaccharomyces pombe*. Mol. Cell. Biol. 38, e00047-18 (2018). 29440310 10.1128/MCB.00047-18 PMC5902593

[30] D. E. Dollins, W. Bai, P. C. Fridy, J. C. Otto, J. L. Neubauer, S. G. Gattis, K. P. M. Mehta, J. D. York, Vip1 is a kinase and pyrophosphatase switch that regulates inositol diphosphate signaling. Proc. Natl. Acad. Sci. U.S.A. 117, 9356–9364 (2020). 32303658 10.1073/pnas.1908875117 PMC7196807

[31] E. Riemer, N. J. Pullagurla, R. Yadav, P. Rana, H. J. Jessen, M. Kamleitner, G. Schaaf, D. Laha, Regulation of plant biotic interactions and abiotic stress responses by inositol polyphosphates. Front. Plant Sci. 13, 944515 (2022). 36035672 10.3389/fpls.2022.944515 PMC9403785

[32] H. Whitfield, G. White, C. Sprigg, A. M. Riley, B. V. L. Potter, A. M. Hemmings, C. A. Brearley, An ATP-responsive metabolic cassette comprised of inositol tris/tetrakisphosphate kinase 1 (ITPK1) and inositol pentakisphosphate 2-kinase (IPK1) buffers diphosphosphoinositol phosphate levels. Biochem. J. 477, 2621–2638 (2020). 32706850 10.1042/BCJ20200423 PMC7115839

[33] J. Shi, X. Wang, E. Wang, Mycorrhizal symbiosis in plant growth and stress adaptation: From genes to ecosystems. Annu. Rev. Plant Biol. 74, 569–607 (2023). 36854473 10.1146/annurev-arplant-061722-090342

[34] M. C. Brundrett, L. Tedersoo, Evolutionary history of mycorrhizal symbioses and global host plant diversity. New Phytol. 220, 1108–1115 (2018). 29355963 10.1111/nph.14976

[35] W. Remy, T. N. Taylor, H. Hass, H. Kerp, Four hundred-million-year-old vesicular arbuscular mycorrhizae. Proc. Natl. Acad. Sci. U.S.A. 91, 11841–11843 (1994). 11607500 10.1073/pnas.91.25.11841 PMC45331

[36] B. J. W. Mills, S. A. Batterman, K. J. Field, Nutrient acquisition by symbiotic fungi governs Palaeozoic climate transition. Philos. Trans. R. Soc. Lond. B Biol. Sci. 373, 20160503 (2018). 29254967 10.1098/rstb.2016.0503 PMC5745338

[37] A. Genre, M. Chabaud, T. Timmers, P. Bonfante, D. G. Barker, Arbuscular mycorrhizal fungi elicit a novel intracellular apparatus in *Medicago truncatula* root epidermal cells before infection. Plant Cell 17, 3489–3499 (2005). 16284314 10.1105/tpc.105.035410 PMC1315383

[38] A. Genre, M. Chabaud, A. Faccio, D. G. Barker, P. Bonfante, Prepenetration apparatus assembly precedes and predicts the colonization patterns of arbuscular mycorrhizal fungi within the root cortex of both *Medicago truncatula* and *Daucus carota*. Plant Cell 20, 1407–1420 (2008). 18515499 10.1105/tpc.108.059014 PMC2438458

[39] M. J. Harrison, Cellular programs for arbuscular mycorrhizal symbiosis. Curr. Opin. Plant Biol. 15, 691–698 (2012). 23036821 10.1016/j.pbi.2012.08.010

[40] S. Campo, B. San Segundo, Systemic induction of phosphatidylinositol-based signaling in leaves of arbuscular mycorrhizal rice plants. Sci. Rep. 10, 15896 (2020). 32985595 10.1038/s41598-020-72985-6 PMC7522983

[41] D. Liao, C. Sun, H. Liang, Y. Wang, X. Bian, C. Dong, X. Niu, M. Yang, G. Xu, A. Chen, S. Wu, SlSPX1-SlPHR complexes mediate the suppression of arbuscular mycorrhizal symbiosis by phosphate repletion in tomato. Plant Cell 34, 4045–4065 (2022). 35863053 10.1093/plcell/koac212 PMC9516199

[42] J. Shi, B. Zhao, S. Zheng, X. Zhang, X. Wang, W. Dong, Q. Xie, G. Wang, Y. Xiao, F. Chen, N. Yu, E. Wang, A phosphate starvation response-centered network regulates mycorrhizal symbiosis. Cell 184, 5527–5540.e18 (2021). 34644527 10.1016/j.cell.2021.09.030

[43] P. Wang, Y. Zhong, Y. Li, W. Zhu, Y. Zhang, J. Li, Z. Chen, E. Limpens, The phosphate starvation response regulator PHR2 antagonizes arbuscule maintenance in Medicago. New Phytol. 244, 1979–1993 (2024). 38803107 10.1111/nph.19869

[44] F. Lota, S. Wegmüller, B. Buer, S. Sato, A. Bräutigam, B. Hanf, M. Bucher, The *cis*-acting CTTC-P1BS module is indicative for gene function of *LjVTI12*, a Qb-SNARE protein gene that is required for arbuscule formation in *Lotus japonicus*. Plant J. 74, 280–293 (2013). 23452278 10.1111/tpj.12120

[45] A. Chen, M. Gu, S. Sun, L. Zhu, S. Hong, G. Xu, Identification of two conserved cis-acting elements, MYCS and P1BS, involved in the regulation of mycorrhiza-activated phosphate transporters in eudicot species. New Phytol. 189, 1157–1169 (2011). 21106037 10.1111/j.1469-8137.2010.03556.x

[46] L. H. Luginbuehl, G. N. Menard, S. Kurup, H. Van Erp, G. V. Radhakrishnan, A. Breakspear, G. E. D. Oldroyd, P. J. Eastmond, Fatty acids in arbuscular mycorrhizal fungi are synthesized by the host plant. Science 356, 1175–1178 (2017). 28596311 10.1126/science.aan0081

[47] Y. Jiang, W. Wang, Q. Xie, N. Liu, L. Liu, D. Wang, X. Zhang, C. Yang, X. Chen, D. Tang, E. Wang, Plants transfer lipids to sustain colonization by mutualistic mycorrhizal and parasitic fungi. Science 356, 1172–1175 (2017). 28596307 10.1126/science.aam9970

[48] A. Keymer, P. Pimprikar, V. Wewer, C. Huber, M. Brands, S. L. Bucerius, P.-M. Delaux, V. Klingl, E. von Röpenack-Lahaye, T. L. Wang, W. Eisenreich, P. Dörmann, M. Parniske, C. Gutjahr, Lipid transfer from plants to arbuscular mycorrhiza fungi. eLife 6, e29107 (2017). 28726631 10.7554/eLife.29107 PMC5559270

[49] S. E. Smith, F. A. Smith, Roles of arbuscular mycorrhizas in plant nutrition and growth: New paradigms from cellular to ecosystem scales. Annu. Rev. Plant Biol. 62, 227–250 (2011). 21391813 10.1146/annurev-arplant-042110-103846

[50] N. Marro, G. Grilli, F. Soteras, M. Caccia, S. Longo, N. Cofré, V. Borda, M. Burni, M. Janoušková, C. Urcelay, The effects of arbuscular mycorrhizal fungal species and taxonomic groups on stressed and unstressed plants: A global meta-analysis. New Phytol. 235, 320–332 (2022). 35302658 10.1111/nph.18102

[51] F. Breuillin, J. Schramm, M. Hajirezaei, A. Ahkami, P. Favre, U. Druege, B. Hause, M. Bucher, T. Kretzschmar, E. Bossolini, C. Kuhlemeier, E. Martinoia, P. Franken, U. Scholz, D. Reinhardt, Phosphate systemically inhibits development of arbuscular mycorrhiza in Petunia hybrida and represses genes involved in mycorrhizal functioning. Plant J. 64, 1002–1017 (2010). 21143680 10.1111/j.1365-313X.2010.04385.x

[52] C. Balzergue, V. Puech-Pagès, G. Bécard, S. F. Rochange, The regulation of arbuscular mycorrhizal symbiosis by phosphate in pea involves early and systemic signalling events. J. Exp. Bot. 62, 1049–1060 (2011). 21045005 10.1093/jxb/erq335 PMC3022399

[53] T. Mun, A. Bachmann, V. Gupta, J. Stougaard, S. U. Andersen, *Lotus* Base: An integrated information portal for the model legume *Lotus japonicus*. Sci. Rep. 6, 39447 (2016). 28008948 10.1038/srep39447 PMC5180183

[54] S. Osada, K. Kageyama, Y. Ohnishi, J.-I. Nishikawa, T. Nishihara, M. Imagawa, Inositol phosphate kinase Vip1p interacts with histone chaperone Asf1p in *Saccharomyces cerevisiae*. Mol. Biol. Rep. 39, 4989–4996 (2012). 22160571 10.1007/s11033-011-1295-z

[55] S. Mulugu, W. Bai, P. C. Fridy, R. J. Bastidas, J. C. Otto, D. E. Dollins, T. A. Haystead, A. A. Ribeiro, J. D. York, A conserved family of enzymes that phosphorylate inositol hexakisphosphate. Science 316, 106–109 (2007). 17412958 10.1126/science.1139099

[56] S. M. N. Onnebo, A. Saiardi, Inositol pyrophosphates modulate hydrogen peroxide signalling. Biochem. J. 423, 109–118 (2009). 19614566 10.1042/BJ20090241

[57] H. Lin, P. C. Fridy, A. A. Ribeiro, J. H. Choi, D. K. Barma, G. Vogel, J. R. Falck, S. B. Shears, J. D. York, G. W. Mayr, Structural analysis and detection of biological inositol pyrophosphates reveal that the family of VIP/diphosphoinositol pentakisphosphate kinases are 1/3-kinases. J. Biol. Chem. 284, 1863–1872 (2009). 18981179 10.1074/jbc.M805686200 PMC2615522

[58] G. Liu, E. Riemer, R. Schneider, D. Cabuzu, O. Bonny, C. A. Wagner, D. Qiu, A. Saiardi, A. Strauss, T. Lahaye, G. Schaaf, T. Knoll, J. P. Jessen, H. J. Jessen, The phytase RipBL1 enables the assignment of a specific inositol phosphate isomer as a structural component of human kidney stones. RSC Chem. Biol. 4, 300–309 (2023). 37034402 10.1039/d2cb00235c PMC10074554

[59] D. Qiu, C. Gu, G. Liu, K. Ritter, V. B. Eisenbeis, T. Bittner, A. Gruzdev, L. Seidel, B. Bengsch, S. B. Shears, H. J. Jessen, Capillary electrophoresis mass spectrometry identifies new isomers of inositol pyrophosphates in mammalian tissues. Chem. Sci. 14, 658–667 (2023). 36741535 10.1039/d2sc05147h PMC9847636

[60] D. Qiu, V. B. Eisenbeis, A. Saiardi, H. J. Jessen, Absolute quantitation of inositol pyrophosphates by capillary electrophoresis electrospray ionization mass spectrometry. J. Vis. Exp., 10.3791/62847-v (2021). 10.3791/62847 34459823

[61] D. Qiu, M. S. Wilson, V. B. Eisenbeis, R. K. Harmel, E. Riemer, T. M. Haas, C. Wittwer, N. Jork, C. Gu, S. B. Shears, G. Schaaf, B. Kammerer, D. Fiedler, A. Saiardi, H. J. Jessen, Analysis of inositol phosphate metabolism by capillary electrophoresis electrospray ionization mass spectrometry. Nat. Commun. 11, 6035 (2020). 33247133 10.1038/s41467-020-19928-x PMC7695695

[62] A. Małolepszy, T. Mun, N. Sandal, V. Gupta, M. Dubin, D. Urbański, N. Shah, A. Bachmann, E. Fukai, H. Hirakawa, S. Tabata, M. Nadzieja, K. Markmann, J. Su, Y. Umehara, T. Soyano, A. Miyahara, S. Sato, M. Hayashi, J. Stougaard, S. U. Andersen, The LORE1 insertion mutant resource. Plant J. 88, 306–317 (2016). 27322352 10.1111/tpj.13243

[63] P. Gaugler, V. Gaugler, M. Kamleitner, G. Schaaf, Extraction and quantification of soluble, radiolabeled inositol polyphosphates from different plant species using SAX-HPLC. J. Vis. Exp., 10.3791/61495 (2020). 10.3791/61495 32658188

[64] D. S. Floss, S. K. Gomez, H.-J. Park, A. M. MacLean, L. M. Müller, K. K. Bhattarai, V. Lévesque-Tremblay, I. E. Maldonado-Mendoza, M. J. Harrison, A transcriptional program for arbuscule degeneration during AM symbiosis is regulated by MYB1. Curr. Biol. 27, 1206–1212 (2017). 28392110 10.1016/j.cub.2017.03.003

[65] L. Arata, E. Fabrizi, P. Sckokai, A worldwide analysis of trend in crop yields and yield variability: Evidence from FAO data. Econ. Model. 90, 190–208 (2020).

[66] R. J. H. Sawers, C. Gutjahr, U. Paszkowski, Cereal mycorrhiza: An ancient symbiosis in modern agriculture. Trends Plant Sci. 13, 93–97 (2008). 18262822 10.1016/j.tplants.2007.11.006

[67] E. Verbruggen, M. G. A. van der Heijden, M. C. Rillig, E. T. Kiers, Mycorrhizal fungal establishment in agricultural soils: Factors determining inoculation success. New Phytol. 197, 1104–1109 (2013). 23495389 10.1111/j.1469-8137.2012.04348.x

[68] M. Moora, J. Davison, M. Öpik, M. Metsis, Ü. Saks, T. Jairus, M. Vasar, M. Zobel, Anthropogenic land use shapes the composition and phylogenetic structure of soil arbuscular mycorrhizal fungal communities. FEMS Microbiol. Ecol. 90, 609–621 (2014). 25187481 10.1111/1574-6941.12420

[69] D. Xiang, E. Verbruggen, Y. Hu, S. D. Veresoglou, M. C. Rillig, W. Zhou, T. Xu, H. Li, Z. Hao, Y. Chen, B. Chen, Land use influences arbuscular mycorrhizal fungal communities in the farming-pastoral ecotone of northern China. New Phytol. 204, 968–978 (2014). 25103342 10.1111/nph.12961

[70] M. Giovannetti, C. Göschl, C. Dietzen, S. U. Andersen, S. Kopriva, W. Busch, Identification of novel genes involved in phosphate accumulation in *Lotus japonicus* through Genome Wide Association mapping of root system architecture and anion content. PLOS Genet. 15, e1008126 (2019). 31856195 10.1371/journal.pgen.1008126 PMC6941899

[71] K. Aung, S.-I. Lin, C.-C. Wu, Y.-T. Huang, C.-L. Su, T.-J. Chiou, pho2, a phosphate overaccumulator, is caused by a nonsense mutation in a microRNA399 target gene. Plant Physiol. 141, 1000–1011 (2006). 16679417 10.1104/pp.106.078063 PMC1489903

[72] R. Bari, B. Datt Pant, M. Stitt, W.-R. Scheible, PHO2, microRNA399, and PHR1 define a phosphate-signaling pathway in plants. Plant Physiol. 141, 988–999 (2006). 16679424 10.1104/pp.106.079707 PMC1489890

[73] S.-I. Lin, S.-F. Chiang, W.-Y. Lin, J.-W. Chen, C.-Y. Tseng, P.-C. Wu, T.-J. Chiou, Regulatory network of microRNA399 and PHO2 by systemic signaling. Plant Physiol. 147, 732–746 (2008). 18390805 10.1104/pp.108.116269 PMC2409027

[74] R. Huertas, I. Torres-Jerez, S. J. Curtin, W. Scheible, M. Udvardi, *Medicago truncatula PHO2* genes have distinct roles in phosphorus homeostasis and symbiotic nitrogen fixation. Front. Plant Sci. 14, 1211107 (2023). 37409286 10.3389/fpls.2023.1211107 PMC10319397

[75] H. Vierheilig, J. M. Garcia-Garrido, U. Wyss, Y. Piché, Systemic suppression of mycorrhizal colonization of barley roots already colonized by AM fungi. Soil Biol. Biochem. 32, 589–595 (2000).

[76] N. Imin, N. A. Mohd-Radzman, H. A. Ogilvie, M. A. Djordjevic, The peptide-encoding CEP1 gene modulates lateral root and nodule numbers in *Medicago truncatula*. J. Exp. Bot. 64, 5395–5409 (2013). 24259455 10.1093/jxb/ert369

[77] L. Pedinotti, J. Teyssendier de la Serve, T. Roudaire, H. San Clemente, M. Aguilar, W. Kohlen, F. Frugier, N. Frei Dit Frey, The CEP peptide-CRA2 receptor module promotes arbuscular mycorrhizal symbiosis. Curr. Biol. 34, 5366–5373.e4 (2024). 39437785 10.1016/j.cub.2024.09.058

[78] N. A. Mohd-Radzman, C. Laffont, A. Ivanovici, N. Patel, D. Reid, J. Stougaard, F. Frugier, N. Imin, M. A. Djordjevic, Different pathways act downstream of the CEP peptide receptor CRA2 to regulate lateral root and nodule development. Plant Physiol. 171, 2536–2548 (2016). 27342310 10.1104/pp.16.00113 PMC4972263

[79] M. G. Mitchum, X. Wang, E. L. Davis, Diverse and conserved roles of CLE peptides. Curr. Opin. Plant Biol. 11, 75–81 (2008). 18078779 10.1016/j.pbi.2007.10.010

[80] R. V. Penmetsa, J. A. Frugoli, L. S. Smith, S. R. Long, D. R. Cook, Dual genetic pathways controlling nodule number in *Medicago truncatula*. Plant Physiol. 131, 998–1008 (2003). 12644652 10.1104/pp.015677 PMC166865

[81] E. Schnabel, E.-P. Journet, F. de Carvalho-Niebel, G. Duc, J. Frugoli, The *Medicago truncatula* SUNN gene encodes a CLV1-like leucine-rich repeat receptor kinase that regulates nodule number and root length. Plant Mol. Biol. 58, 809–822 (2005). 16240175 10.1007/s11103-005-8102-y

[82] M. Karlo, C. Boschiero, K. G. Landerslev, G. S. Blanco, J. Wen, K. S. Mysore, X. Dai, P. X. Zhao, T. C. de Bang, The CLE53-SUNN genetic pathway negatively regulates arbuscular mycorrhiza root colonization in *Medicago truncatula*. J. Exp. Bot. 71, 4972–4984 (2020). 32309861 10.1093/jxb/eraa193 PMC7410177

[83] L. M. Müller, K. Flokova, E. Schnabel, X. Sun, Z. Fei, J. Frugoli, H. J. Bouwmeester, M. J. Harrison, A CLE-SUNN module regulates strigolactone content and fungal colonization in arbuscular mycorrhiza. Nat. Plants 5, 933–939 (2019). 31477892 10.1038/s41477-019-0501-1

[84] R. Müller, A. Bleckmann, R. Simon, The receptor kinase CORYNE of *Arabidopsis* transmits the stem cell-limiting signal CLAVATA3 independently of CLAVATA1. Plant Cell 20, 934–946 (2008). 18381924 10.1105/tpc.107.057547 PMC2390746

[85] S. Bashyal, H. Everett, S. Matsuura, L. M. Müller, A plant CLE peptide and its fungal mimic promote arbuscular mycorrhizal symbiosis via CRN-mediated ROS suppression. Proc. Natl. Acad. Sci. U.S.A. 122, e2422215122 (2025). 40228122 10.1073/pnas.2422215122 PMC12037060

[86] M. Le Marquer, G. Bécard, N. Frei Dit Frey, Arbuscular mycorrhizal fungi possess a CLAVATA3/embryo surrounding region-related gene that positively regulates symbiosis. New Phytol. 222, 1030–1042 (2019). 30554405 10.1111/nph.15643

[87] S. M. Schmidt, M. Belisle, W. B. Frommer, The evolving landscape around genome editing in agriculture: Many countries have exempted or move to exempt forms of genome editing from GMO regulation of crop plants. EMBO Rep. 21, e50680 (2020). 32431018 10.15252/embr.202050680 PMC7271327

[88] N. P. Laha, R. F. H. Giehl, E. Riemer, D. Qiu, N. J. Pullagurla, R. Schneider, Y. W. Dhir, R. Yadav, Y. E. Mihiret, P. Gaugler, V. Gaugler, H. Mao, N. Zheng, N. von Wirén, A. Saiardi, S. Bhattacharjee, H. J. Jessen, D. Laha, G. Schaaf, INOSITOL (1,3,4) TRIPHOSPHATE 5/6 KINASE1-dependent inositol polyphosphates regulate auxin responses in Arabidopsis. Plant Physiol. 190, 2722–2738 (2022). 36124979 10.1093/plphys/kiac425 PMC9706486

[89] P. Gaugler, R. Schneider, G. Liu, D. Qiu, J. Weber, J. Schmid, N. Jork, M. Häner, K. Ritter, N. Fernández-Rebollo, R. F. H. Giehl, M. N. Trung, R. Yadav, D. Fiedler, V. Gaugler, H. J. Jessen, G. Schaaf, D. Laha, *Arabidopsis* PFA-DSP-type phosphohydrolases target specific inositol pyrophosphate messengers. Biochemistry 61, 1213–1227 (2022). 35640071 10.1021/acs.biochem.2c00145 PMC9351621

[90] F. Laurent, S. M. Bartsch, A. Shukla, F. Rico-Resendiz, D. Couto, C. Fuchs, J. Nicolet, S. Loubéry, H. J. Jessen, D. Fiedler, M. Hothorn, Inositol pyrophosphate catabolism by three families of phosphatases regulates plant growth and development. PLOS Genet. 20, e1011468 (2024). 39531477 10.1371/journal.pgen.1011468 PMC11611267

[91] K. Chalak, R. Yadav, G. Liu, P. Rana, H. J. Jessen, D. Laha, Functional conservation of the DDP1-type inositol pyrophosphate phosphohydrolases in land plant. Biochemistry 63, 2723–2728 (2024). 39404446 10.1021/acs.biochem.4c00458

[92] C. Freed, B. Craige, J. Donahue, C. Cridland, S. P. Williams, C. Pereira, J. Kim, H. Blice, J. Owen Jr., G. Gillaspy, Using native and synthetic genes to disrupt inositol pyrophosphates and phosphate accumulation in plants. Plant Physiol. 197, kiae582 (2024). 39474910 10.1093/plphys/kiae582 PMC11663554

[93] R. Schneider, K. Lami, I. Prucker, S. C. Stolze, A. Strauß, J. M. Schmidt, S. M. Bartsch, K. Langenbach, E. Lange, K. Ritter, D. Furkert, N. Faiß, S. Kumar, M. Shamim Hasan, A. Makris, L. Krusenbaum, S. Wege, Y. Z. Belay, S. Kriescher, J. The, M. Harings, F. M. W. Grundler, M. K. Ried-Lasi, H. Schoof, P. Gaugler, M. Kamleitner, D. Fiedler, H. Nakagami, R. F. H. Giehl, T. Lahaye, S. Bhattacharjee, H. J. Jessen, V. Gaugler, G. Schaaf, NUDIX hydrolases target specific inositol pyrophosphates and regulate phosphate homeostasis and bacterial pathogen susceptibility in *Arabidopsis*. J. Integr. Plant Biol. 67, 3123–3151 (2025). 41165244 10.1111/jipb.70060 PMC12678693

[94] S. E. Smith, D. J. Read, *Mycorrhizal Symbiosis* (Academic Press, ed. 3, 2010).

[95] M. Bigalke, A. Ulrich, A. Rehmus, A. Keller, Accumulation of cadmium and uranium in arable soils in Switzerland. Environ. Pollut. 221, 85–93 (2017). 27908488 10.1016/j.envpol.2016.11.035

[96] M. Abdalla, M. Bitterlich, J. Jansa, D. Püschel, M. A. Ahmed, The role of arbuscular mycorrhizal symbiosis in improving plant water status under drought. J. Exp. Bot. 74, 4808–4824 (2023). 37409696 10.1093/jxb/erad249

[97] H.-J. Hawkins, R. I. M. Cargill, M. E. Van Nuland, S. C. Hagen, K. J. Field, M. Sheldrake, N. A. Soudzilovskaia, E. T. Kiers, Mycorrhizal mycelium as a global carbon pool. Curr. Biol. 33, R560–R573 (2023). 37279689 10.1016/j.cub.2023.02.027

[98] S. Alberti, A. D. Gitler, S. Lindquist, A suite of Gateway cloning vectors for high-throughput genetic analysis in *Saccharomyces cerevisiae*. Yeast 24, 913–919 (2007). 17583893 10.1002/yea.1502 PMC2190539

[99] H. Vierheilig, A. P. Coughlan, U. Wyss, Y. Piché, Ink and vinegar, a simple staining technique for arbuscular-mycorrhizal fungi. Appl. Environ. Microbiol. 64, 5004–5007 (1998). 9835596 10.1128/aem.64.12.5004-5007.1998 PMC90956

[100] T. P. McGonigle, M. H. Miller, D. G. Evans, G. L. Fairchild, J. A. Swan, A new method which gives an objective measure of colonization of roots by vesicular-arbuscular mycorrhizal fungi. New Phytol. 115, 495–501 (1990). 33874272 10.1111/j.1469-8137.1990.tb00476.x

[101] D. Dreher, H. Yadav, S. Zander, B. Hause, Is there genetic variation in mycorrhization of *Medicago truncatula*? PeerJ 5, e3713 (2017). 28894638 10.7717/peerj.3713 PMC5592082

[102] M. Sexauer, H. Bhasin, M. Schön, E. Roitsch, C. Wall, U. Herzog, K. Markmann, A micro RNA mediates shoot control of root branching. Nat. Commun. 14, 8083 (2023). 38057302 10.1038/s41467-023-43738-6 PMC10700597

[103] R. D. Gietz, R. H. Schiestl, A. R. Willems, R. A. Woods, Studies on the transformation of intact yeast cells by the LiAc/SS-DNA/PEG procedure. Yeast 11, 355–360 (1995). 7785336 10.1002/yea.320110408

[104] B. J. M. Zonneveld, Cheap and simple yeast media. J. Microbiol. Methods 4, 287–291 (1986).

[105] C. Azevedo, A. Saiardi, Extraction and analysis of soluble inositol polyphosphates from yeast. Nat. Protoc. 1, 2416–2422 (2006). 17406485 10.1038/nprot.2006.337

[106] O. Losito, Z. Szijgyarto, A. C. Resnick, A. Saiardi, Inositol pyrophosphates and their unique metabolic complexity: Analysis by gel electrophoresis. PLOS ONE 4, e5580 (2009). 19440344 10.1371/journal.pone.0005580 PMC2680042

[107] D. Laha, N. Parvin, A. Hofer, R. F. H. Giehl, N. Fernandez-Rebollo, N. von Wirén, A. Saiardi, H. J. Jessen, G. Schaaf, *Arabidopsis* ITPK1 and ITPK2 have an evolutionarily conserved phytic acid kinase activity. ACS Chem. Biol. 14, 2127–2133 (2019). 31525024 10.1021/acschembio.9b00423

[108] R. K. Harmel, R. Puschmann, M. Nguyen Trung, A. Saiardi, P. Schmieder, D. Fiedler, Harnessing 13C-labeled myo-inositol to interrogate inositol phosphate messengers by NMR. Chem. Sci. 10, 5267–5274 (2019). 31191882 10.1039/c9sc00151d PMC6540952

[109] R. Schneider, K. Lami, I. Prucker, S. C. Stolze, A. Strauß, K. Langenbach, M. Kamleitner, Y. Z. Belay, K. Ritter, D. Furkert, P. Gaugler, E. Lange, N. Faiß, J. M. Schmidt, M. Harings, L. Krusenbaum, S. Wege, S. Kriescher, The Jeremy, H. Schoof, D. Fiedler, H. Nakagami, R. F. H. Giehl, T. Lahaye, H. J. Jessen, V. Gaugler, G. Schaaf, NUDIX hydrolases target specific inositol pyrophosphates and regulate phosphate and iron homeostasis, and the expression of defense genes in Arabidopsis. bioRxiv 619122 [Preprint] (2024). 10.1101/2024.10.18.619122.

[110] M. Blum, H.-Y. Chang, S. Chuguransky, T. Grego, S. Kandasaamy, A. Mitchell, G. Nuka, T. Paysan-Lafosse, M. Qureshi, S. Raj, L. Richardson, G. A. Salazar, L. Williams, P. Bork, A. Bridge, J. Gough, D. H. Haft, I. Letunic, A. Marchler-Bauer, H. Mi, D. A. Natale, M. Necci, C. A. Orengo, A. P. Pandurangan, C. Rivoire, C. J. A. Sigrist, I. Sillitoe, N. Thanki, P. D. Thomas, S. C. E. Tosatto, C. H. Wu, A. Bateman, R. D. Finn, The InterPro protein families and domains database: 20 Years on. Nucleic Acids Res. 49, D344–D354 (2021). 33156333 10.1093/nar/gkaa977 PMC7778928

[111] R. C. Edgar, MUSCLE: Multiple sequence alignment with high accuracy and high throughput. Nucleic Acids Res. 32, 1792–1797 (2004). 15034147 10.1093/nar/gkh340 PMC390337

[112] L.-T. Nguyen, H. A. Schmidt, A. von Haeseler, B. Q. Minh, IQ-TREE: A fast and effective stochastic algorithm for estimating maximum-likelihood phylogenies. Mol. Biol. Evol. 32, 268–274 (2015). 25371430 10.1093/molbev/msu300 PMC4271533

[113] I. Letunic, P. Bork, Interactive Tree of Life (iTOL) v6: Recent updates to the phylogenetic tree display and annotation tool. Nucleic Acids Res. 52, W78–W82 (2024). 38613393 10.1093/nar/gkae268 PMC11223838

[114] V. Volpe, M. Giovannetti, X.-G. Sun, V. Fiorilli, P. Bonfante, The phosphate transporters LjPT4 and MtPT4 mediate early root responses to phosphate status in non mycorrhizal roots. Plant Cell Environ. 39, 660–671 (2016). 26476189 10.1111/pce.12659

[115] M. Groth, S. Kosuta, C. Gutjahr, K. Haage, S. L. Hardel, M. Schaub, A. Brachmann, S. Sato, S. Tabata, K. Findlay, T. L. Wang, M. Parniske, Two *Lotus japonicus* symbiosis mutants impaired at distinct steps of arbuscule development. Plant J. 75, 117–129 (2013). 23627596 10.1111/tpj.12220

[116] I. V. Grigoriev, R. D. Hayes, S. Calhoun, B. Kamel, A. Wang, S. Ahrendt, S. Dusheyko, R. Nikitin, S. J. Mondo, A. Salamov, I. Shabalov, A. Kuo, PhycoCosm, a comparative algal genomics resource. Nucleic Acids Res. 49, D1004–D1011 (2021). 33104790 10.1093/nar/gkaa898 PMC7779022

[117] Y. Lee, C. H. Cho, C. Noh, J. H. Yang, S. I. Park, Y. M. Lee, J. A. West, D. Bhattacharya, K. Jo, H. S. Yoon, Origin of minicircular mitochondrial genomes in red algae. Nat. Commun. 14, 3363 (2023). 37291154 10.1038/s41467-023-39084-2 PMC10250338

[118] A. W. Rossoni, D. C. Price, M. Seger, D. Lyska, P. Lammers, D. Bhattacharya, A. P. M. Weber, The genomes of polyextremophilic cyanidiales contain 1% horizontally transferred genes with diverse adaptive functions. eLife 8, e45017 (2019). 31149898 10.7554/eLife.45017 PMC6629376

[119] J. Lee, D. Kim, D. Bhattacharya, H. S. Yoon, Expansion of phycobilisome linker gene families in mesophilic red algae. Nat. Commun. 10, 4823 (2019). 31645564 10.1038/s41467-019-12779-1 PMC6811547

[120] D. C. Price, U. W. Goodenough, R. Roth, J.-H. Lee, T. Kariyawasam, M. Mutwil, C. Ferrari, F. Facchinelli, S. G. Ball, U. Cenci, C. X. Chan, N. E. Wagner, H. S. Yoon, A. P. M. Weber, D. Bhattacharya, Analysis of an improved *Cyanophora paradoxa* genome assembly. DNA Res. 26, 287–299 (2019). 31098614 10.1093/dnares/dsz009 PMC6704402

[121] T. Nishiyama, H. Sakayama, J. de Vries, H. Buschmann, D. Saint-Marcoux, K. K. Ullrich, F. B. Haas, L. Vanderstraeten, D. Becker, D. Lang, S. Vosolsobě, S. Rombauts, P. K. I. Wilhelmsson, P. Janitza, R. Kern, A. Heyl, F. Rümpler, L. I. A. C. Villalobos, J. M. Clay, R. Skokan, A. Toyoda, Y. Suzuki, H. Kagoshima, E. Schijlen, N. Tajeshwar, B. Catarino, A. J. Hetherington, A. Saltykova, C. Bonnot, H. Breuninger, A. Symeonidi, G. V. Radhakrishnan, F. Van Nieuwerburgh, D. Deforce, C. Chang, K. G. Karol, R. Hedrich, P. Ulvskov, G. Glöckner, C. F. Delwiche, J. Petrášek, Y. Van de Peer, J. Friml, M. Beilby, L. Dolan, Y. Kohara, S. Sugano, A. Fujiyama, P.-M. Delaux, M. Quint, G. Theißen, M. Hagemann, J. Harholt, C. Dunand, S. Zachgo, J. Langdale, F. Maumus, D. Van Der Straeten, S. B. Gould, S. A. Rensing, The Chara genome: Secondary complexity and implications for plant terrestrialization. Cell 174, 448–464.e24 (2018). 30007417 10.1016/j.cell.2018.06.033

[122] D. M. Goodstein, S. Shu, R. Howson, R. Neupane, R. D. Hayes, J. Fazo, T. Mitros, W. Dirks, U. Hellsten, N. Putnam, D. S. Rokhsar, Phytozome: A comparative platform for green plant genomics. Nucleic Acids Res. 40, D1178–D1186 (2012). 22110026 10.1093/nar/gkr944 PMC3245001

[123] S. S. Merchant, S. E. Prochnik, O. Vallon, E. H. Harris, S. J. Karpowicz, G. B. Witman, A. Terry, A. Salamov, L. K. Fritz-Laylin, L. Maréchal-Drouard, W. F. Marshall, L.-H. Qu, D. R. Nelson, A. A. Sanderfoot, M. H. Spalding, V. V. Kapitonov, Q. Ren, P. Ferris, E. Lindquist, H. Shapiro, S. M. Lucas, J. Grimwood, J. Schmutz, P. Cardol, H. Cerutti, G. Chanfreau, C.-L. Chen, V. Cognat, M. T. Croft, R. Dent, S. Dutcher, E. Fernández, H. Fukuzawa, D. González-Ballester, D. González-Halphen, A. Hallmann, M. Hanikenne, M. Hippler, W. Inwood, K. Jabbari, M. Kalanon, R. Kuras, P. A. Lefebvre, S. D. Lemaire, A. V. Lobanov, M. Lohr, A. Manuell, I. Meier, L. Mets, M. Mittag, T. Mittelmeier, J. V. Moroney, J. Moseley, C. Napoli, A. M. Nedelcu, K. Niyogi, S. V. Novoselov, I. T. Paulsen, G. Pazour, S. Purton, J.-P. Ral, D. M. Riaño-Pachón, W. Riekhof, L. Rymarquis, M. Schroda, D. Stern, J. Umen, R. Willows, N. Wilson, S. L. Zimmer, J. Allmer, J. Balk, K. Bisova, C.-J. Chen, M. Elias, K. Gendler, C. Hauser, M. R. Lamb, H. Ledford, J. C. Long, J. Minagawa, M. D. Page, J. Pan, W. Pootakham, S. Roje, A. Rose, E. Stahlberg, A. M. Terauchi, P. Yang, S. Ball, C. Bowler, C. L. Dieckmann, V. N. Gladyshev, P. Green, R. Jorgensen, S. Mayfield, B. Mueller-Roeber, S. Rajamani, R. T. Sayre, P. Brokstein, I. Dubchak, D. Goodstein, L. Hornick, Y. W. Huang, J. Jhaveri, Y. Luo, D. Martínez, W. C. A. Ngau, B. Otillar, A. Poliakov, A. Porter, L. Szajkowski, G. Werner, K. Zhou, I. V. Grigoriev, D. S. Rokhsar, A. R. Grossman, The *Chlamydomonas* genome reveals the evolution of key animal and plant functions. Science 318, 245–250 (2007). 17932292 10.1126/science.1143609 PMC2875087

[124] S. E. Prochnik, J. Umen, A. M. Nedelcu, A. Hallmann, S. M. Miller, I. Nishii, P. Ferris, A. Kuo, T. Mitros, L. K. Fritz-Laylin, U. Hellsten, J. Chapman, O. Simakov, S. A. Rensing, A. Terry, J. Pangilinan, V. Kapitonov, J. Jurka, A. Salamov, H. Shapiro, J. Schmutz, J. Grimwood, E. Lindquist, S. Lucas, I. V. Grigoriev, R. Schmitt, D. Kirk, D. S. Rokhsar, Genomic analysis of organismal complexity in the multicellular green alga *Volvox carteri*. Science 329, 223–226 (2010). 20616280 10.1126/science.1188800 PMC2993248

[125] G. V. Radhakrishnan, J. Keller, M. K. Rich, T. Vernié, D. L. Mbadinga Mbadinga, N. Vigneron, L. Cottret, H. S. Clemente, C. Libourel, J. Cheema, A.-M. Linde, D. M. Eklund, S. Cheng, G. K. S. Wong, U. Lagercrantz, F.-W. Li, G. E. D. Oldroyd, P.-M. Delaux, An ancestral signalling pathway is conserved in intracellular symbioses-forming plant lineages. Nat. Plants 6, 280–289 (2020). 32123350 10.1038/s41477-020-0613-7

[126] J. L. Bowman, T. Kohchi, K. T. Yamato, J. Jenkins, S. Shu, K. Ishizaki, S. Yamaoka, R. Nishihama, Y. Nakamura, F. Berger, C. Adam, S. S. Aki, F. Althoff, T. Araki, M. A. Arteaga-Vazquez, S. Balasubrmanian, K. Barry, D. Bauer, C. R. Boehm, L. Briginshaw, J. Caballero-Perez, B. Catarino, F. Chen, S. Chiyoda, M. Chovatia, K. M. Davies, M. Delmans, T. Demura, T. Dierschke, L. Dolan, A. E. Dorantes-Acosta, D. M. Eklund, S. N. Florent, E. Flores-Sandoval, A. Fujiyama, H. Fukuzawa, B. Galik, D. Grimanelli, J. Grimwood, U. Grossniklaus, T. Hamada, J. Haseloff, A. J. Hetherington, A. Higo, Y. Hirakawa, H. N. Hundley, Y. Ikeda, K. Inoue, S.-I. Inoue, S. Ishida, Q. Jia, M. Kakita, T. Kanazawa, Y. Kawai, T. Kawashima, M. Kennedy, K. Kinose, T. Kinoshita, Y. Kohara, E. Koide, K. Komatsu, S. Kopischke, M. Kubo, J. Kyozuka, U. Lagercrantz, S.-S. Lin, E. Lindquist, A. M. Lipzen, C.-W. Lu, E. De Luna, R. A. Martienssen, N. Minamino, M. Mizutani, M. Mizutani, N. Mochizuki, I. Monte, R. Mosher, H. Nagasaki, H. Nakagami, S. Naramoto, K. Nishitani, M. Ohtani, T. Okamoto, M. Okumura, J. Phillips, B. Pollak, A. Reinders, M. Rövekamp, R. Sano, S. Sawa, M. W. Schmid, M. Shirakawa, R. Solano, A. Spunde, N. Suetsugu, S. Sugano, A. Sugiyama, R. Sun, Y. Suzuki, M. Takenaka, D. Takezawa, H. Tomogane, M. Tsuzuki, T. Ueda, M. Umeda, J. M. Ward, Y. Watanabe, K. Yazaki, R. Yokoyama, Y. Yoshitake, I. Yotsui, S. Zachgo, J. Schmutz, Insights into land plant evolution garnered from the Marchantia polymorpha genome. Cell 171, 287–304.e15 (2017). 28985561 10.1016/j.cell.2017.09.030

[127] D. Lang, K. K. Ullrich, F. Murat, J. Fuchs, J. Jenkins, F. B. Haas, M. Piednoel, H. Gundlach, M. Van Bel, R. Meyberg, C. Vives, J. Morata, A. Symeonidi, M. Hiss, W. Muchero, Y. Kamisugi, O. Saleh, G. Blanc, E. L. Decker, N. van Gessel, J. Grimwood, R. D. Hayes, S. W. Graham, L. E. Gunter, S. F. McDaniel, S. N. W. Hoernstein, A. Larsson, F.-W. Li, P.-F. Perroud, J. Phillips, P. Ranjan, D. S. Rokshar, C. J. Rothfels, L. Schneider, S. Shu, D. W. Stevenson, F. Thümmler, M. Tillich, J. C. Villarreal Aguilar, T. Widiez, G. K.-S. Wong, A. Wymore, Y. Zhang, A. D. Zimmer, R. S. Quatrano, K. F. X. Mayer, D. Goodstein, J. M. Casacuberta, K. Vandepoele, R. Reski, A. C. Cuming, G. A. Tuskan, F. Maumus, J. Salse, J. Schmutz, S. A. Rensing, The *Physcomitrella patens* chromosome-scale assembly reveals moss genome structure and evolution. Plant J. 93, 515–533 (2018). 29237241 10.1111/tpj.13801

[128] J. A. Banks, T. Nishiyama, M. Hasebe, J. L. Bowman, M. Gribskov, C. dePamphilis, V. A. Albert, N. Aono, T. Aoyama, B. A. Ambrose, N. W. Ashton, M. J. Axtell, E. Barker, M. S. Barker, J. L. Bennetzen, N. D. Bonawitz, C. Chapple, C. Cheng, L. G. G. Correa, M. Dacre, J. DeBarry, I. Dreyer, M. Elias, E. M. Engstrom, M. Estelle, L. Feng, C. Finet, S. K. Floyd, W. B. Frommer, T. Fujita, L. Gramzow, M. Gutensohn, J. Harholt, M. Hattori, A. Heyl, T. Hirai, Y. Hiwatashi, M. Ishikawa, M. Iwata, K. G. Karol, B. Koehler, U. Kolukisaoglu, M. Kubo, T. Kurata, S. Lalonde, K. Li, Y. Li, A. Litt, E. Lyons, G. Manning, T. Maruyama, T. P. Michael, K. Mikami, S. Miyazaki, S.-I. Morinaga, T. Murata, B. Mueller-Roeber, D. R. Nelson, M. Obara, Y. Oguri, R. G. Olmstead, N. Onodera, B. L. Petersen, B. Pils, M. Prigge, S. A. Rensing, D. M. Riaño-Pachón, A. W. Roberts, Y. Sato, H. V. Scheller, B. Schulz, C. Schulz, E. V. Shakirov, N. Shibagaki, N. Shinohara, D. E. Shippen, I. Sørensen, R. Sotooka, N. Sugimoto, M. Sugita, N. Sumikawa, M. Tanurdzic, G. Theissen, P. Ulvskov, S. Wakazuki, J.-K. Weng, W. W. G. T. Willats, D. Wipf, P. G. Wolf, L. Yang, A. D. Zimmer, Q. Zhu, T. Mitros, U. Hellsten, D. Loqué, R. Otillar, A. Salamov, J. Schmutz, H. Shapiro, E. Lindquist, S. Lucas, D. Rokhsar, I. V. Grigoriev, The Selaginella genome identifies genetic changes associated with the evolution of vascular plants. Science 332, 960–963 (2011). 21551031 10.1126/science.1203810 PMC3166216

[129] K.-J. Gu, C.-F. Lin, J.-J. Wu, Y.-P. Zhao, GinkgoDB: An ecological genome database for the living fossil, *Ginkgo biloba*. Database 2022, baac046 (2022). 35758513 10.1093/database/baac046 PMC9235008

[130] Amborella Genome Project, The *Amborella* genome and the evolution of flowering plants. Science 342, 1241089 (2013). 24357323 10.1126/science.1241089

[131] L. Qin, Y. Hu, J. Wang, X. Wang, R. Zhao, H. Shan, K. Li, P. Xu, H. Wu, X. Yan, L. Liu, X. Yi, S. Wanke, J. E. Bowers, J. H. Leebens-Mack, C. W. dePamphilis, P. S. Soltis, D. E. Soltis, H. Kong, Y. Jiao, Insights into angiosperm evolution, floral development and chemical biosynthesis from the *Aristolochia fimbriata* genome. Nat. Plants 7, 1239–1253 (2021). 34475528 10.1038/s41477-021-00990-2 PMC8445822

[132] J. Cai, X. Liu, K. Vanneste, S. Proost, W.-C. Tsai, K.-W. Liu, L.-J. Chen, Y. He, Q. Xu, C. Bian, Z. Zheng, F. Sun, W. Liu, Y.-Y. Hsiao, Z.-J. Pan, C.-C. Hsu, Y.-P. Yang, Y.-C. Hsu, Y.-C. Chuang, A. Dievart, J.-F. Dufayard, X. Xu, J.-Y. Wang, J. Wang, X.-J. Xiao, X.-M. Zhao, R. Du, G.-Q. Zhang, M. Wang, Y.-Y. Su, G.-C. Xie, G.-H. Liu, L.-Q. Li, L.-Q. Huang, Y.-B. Luo, H.-H. Chen, Y. Van de Peer, Z.-J. Liu, The genome sequence of the orchid *Phalaenopsis equestris*. Nat. Genet. 47, 65–72 (2015). 25420146 10.1038/ng.3149

[133] A. D’Hont, F. Denoeud, J.-M. Aury, F.-C. Baurens, F. Carreel, O. Garsmeur, B. Noel, S. Bocs, G. Droc, M. Rouard, C. Da Silva, K. Jabbari, C. Cardi, J. Poulain, M. Souquet, K. Labadie, C. Jourda, J. Lengellé, M. Rodier-Goud, A. Alberti, M. Bernard, M. Correa, S. Ayyampalayam, M. R. Mckain, J. Leebens-Mack, D. Burgess, M. Freeling, D. Mbéguié-A-Mbéguié, M. Chabannes, T. Wicker, O. Panaud, J. Barbosa, E. Hribova, P. Heslop-Harrison, R. Habas, R. Rivallan, P. Francois, C. Poiron, A. Kilian, D. Burthia, C. Jenny, F. Bakry, S. Brown, V. Guignon, G. Kema, M. Dita, C. Waalwijk, S. Joseph, A. Dievart, O. Jaillon, J. Leclercq, X. Argout, E. Lyons, A. Almeida, M. Jeridi, J. Dolezel, N. Roux, A.-M. Risterucci, J. Weissenbach, M. Ruiz, J.-C. Glaszmann, F. Quétier, N. Yahiaoui, P. Wincker, The banana (*Musa acuminata*) genome and the evolution of monocotyledonous plants. Nature 488, 213–217 (2012). 22801500 10.1038/nature11241

[134] N. Bornowski, K. J. Michel, J. P. Hamilton, S. Ou, A. S. Seetharam, J. Jenkins, J. Grimwood, C. Plott, S. Shu, J. Talag, M. Kennedy, H. Hundley, V. R. Singan, K. Barry, C. Daum, Y. Yoshinaga, J. Schmutz, C. N. Hirsch, M. B. Hufford, N. de Leon, S. M. Kaeppler, C. R. Buell, Genomic variation within the maize stiff-stalk heterotic germplasm pool. Plant Genome 14, e20114 (2021). 34275202 10.1002/tpg2.20114 PMC12806955

[135] H. Sakai, S. S. Lee, T. Tanaka, H. Numa, J. Kim, Y. Kawahara, H. Wakimoto, C.-C. Yang, M. Iwamoto, T. Abe, Y. Yamada, A. Muto, H. Inokuchi, T. Ikemura, T. Matsumoto, T. Sasaki, T. Itoh, Rice Annotation Project Database (RAP-DB): An integrative and interactive database for rice genomics. Plant Cell Physiol. 54, e6 (2013). 23299411 10.1093/pcp/pcs183 PMC3583025

[136] M. Mascher, T. Wicker, J. Jenkins, C. Plott, T. Lux, C. S. Koh, J. Ens, H. Gundlach, L. B. Boston, Z. Tulpová, S. Holden, I. Hernández-Pinzón, U. Scholz, K. F. X. Mayer, M. Spannagl, C. J. Pozniak, A. G. Sharpe, H. Šimková, M. J. Moscou, J. Grimwood, J. Schmutz, N. Stein, Long-read sequence assembly: A technical evaluation in barley. Plant Cell 33, 1888–1906 (2021). 33710295 10.1093/plcell/koab077 PMC8290290

[137] J. M. McGrath, A. Funk, P. Galewski, S. Ou, B. Townsend, K. Davenport, H. Daligault, S. Johnson, J. Lee, A. Hastie, A. Darracq, G. Willems, S. Barnes, I. Liachko, S. Sullivan, S. Koren, A. Phillippy, J. Wang, T. Liu, J. Pulman, K. Childs, S. Shu, A. Yocum, D. Fermin, E. Mutasa-Göttgens, P. Stevanato, K. Taguchi, R. Naegele, K. M. Dorn, A contiguous *de novo* genome assembly of sugar beet EL10 (*Beta vulgaris* L.). DNA Res. 30, dsac033 (2023). 36208288 10.1093/dnares/dsac033 PMC9896481

[138] R. van Velzen, R. Holmer, F. Bu, L. Rutten, A. van Zeijl, W. Liu, L. Santuari, Q. Cao, T. Sharma, D. Shen, Y. Roswanjaya, T. A. K. Wardhani, M. S. Kalhor, J. Jansen, J. van den Hoogen, B. Güngör, M. Hartog, J. Hontelez, J. Verver, W.-C. Yang, E. Schijlen, R. Repin, M. Schilthuizen, M. E. Schranz, R. Heidstra, K. Miyata, E. Fedorova, W. Kohlen, T. Bisseling, S. Smit, R. Geurts, Comparative genomics of the nonlegume Parasponia reveals insights into evolution of nitrogen-fixing rhizobium symbioses. Proc. Natl. Acad. Sci. U.S.A. 115, E4700–E4709 (2018). 29717040 10.1073/pnas.1721395115 PMC5960304

[139] M. Griesmann, Y. Chang, X. Liu, Y. Song, G. Haberer, M. B. Crook, B. Billault-Penneteau, D. Lauressergues, J. Keller, L. Imanishi, Y. P. Roswanjaya, W. Kohlen, P. Pujic, K. Battenberg, N. Alloisio, Y. Liang, H. Hilhorst, M. G. Salgado, V. Hocher, H. Gherbi, S. Svistoonoff, J. J. Doyle, S. He, Y. Xu, S. Xu, J. Qu, Q. Gao, X. Fang, Y. Fu, P. Normand, A. M. Berry, L. G. Wall, J.-M. Ané, K. Pawlowski, X. Xu, H. Yang, M. Spannagl, K. F. X. Mayer, G. K.-S. Wong, M. Parniske, P.-M. Delaux, S. Cheng, Phylogenomics reveals multiple losses of nitrogen-fixing root nodule symbiosis. Science 361, eaat1743 (2018). 29794220 10.1126/science.aat1743

[140] B. Hufnagel, A. Marques, A. Soriano, L. Marquès, F. Divol, P. Doumas, E. Sallet, D. Mancinotti, S. Carrere, W. Marande, S. Arribat, J. Keller, C. Huneau, T. Blein, D. Aimé, M. Laguerre, J. Taylor, V. Schubert, M. Nelson, F. Geu-Flores, M. Crespi, K. Gallardo, P.-M. Delaux, J. Salse, H. Bergès, R. Guyot, J. Gouzy, B. Péret, High-quality genome sequence of white lupin provides insight into soil exploration and seed quality. Nat. Commun. 11, 492 (2020). 31980615 10.1038/s41467-019-14197-9 PMC6981116

[141] N. D. Young, F. Debellé, G. E. D. Oldroyd, R. Geurts, S. B. Cannon, M. K. Udvardi, V. A. Benedito, K. F. X. Mayer, J. Gouzy, H. Schoof, Y. Van de Peer, S. Proost, D. R. Cook, B. C. Meyers, M. Spannagl, F. Cheung, S. De Mita, V. Krishnakumar, H. Gundlach, S. Zhou, J. Mudge, A. K. Bharti, J. D. Murray, M. A. Naoumkina, B. Rosen, K. A. T. Silverstein, H. Tang, S. Rombauts, P. X. Zhao, P. Zhou, V. Barbe, P. Bardou, M. Bechner, A. Bellec, A. Berger, H. Bergès, S. Bidwell, T. Bisseling, N. Choisne, A. Couloux, R. Denny, S. Deshpande, X. Dai, J. J. Doyle, A.-M. Dudez, A. D. Farmer, S. Fouteau, C. Franken, C. Gibelin, J. Gish, S. Goldstein, A. J. González, P. J. Green, A. Hallab, M. Hartog, A. Hua, S. J. Humphray, D.-H. Jeong, Y. Jing, A. Jöcker, S. M. Kenton, D.-J. Kim, K. Klee, H. Lai, C. Lang, S. Lin, S. L. Macmil, G. Magdelenat, L. Matthews, J. McCorrison, E. L. Monaghan, J.-H. Mun, F. Z. Najar, C. Nicholson, C. Noirot, M. O’Bleness, C. R. Paule, J. Poulain, F. Prion, B. Qin, C. Qu, E. F. Retzel, C. Riddle, E. Sallet, S. Samain, N. Samson, I. Sanders, O. Saurat, C. Scarpelli, T. Schiex, B. Segurens, A. J. Severin, D. J. Sherrier, R. Shi, S. Sims, S. R. Singer, S. Sinharoy, L. Sterck, A. Viollet, B.-B. Wang, K. Wang, M. Wang, X. Wang, J. Warfsmann, J. Weissenbach, D. D. White, J. D. White, G. B. Wiley, P. Wincker, Y. Xing, L. Yang, Z. Yao, F. Ying, J. Zhai, L. Zhou, A. Zuber, J. Dénarié, R. A. Dixon, G. D. May, D. C. Schwartz, J. Rogers, F. Quétier, C. D. Town, B. A. Roe, The *Medicago* genome provides insight into the evolution of rhizobial symbioses. Nature 480, 520–524 (2011). 22089132 10.1038/nature10625 PMC3272368

[142] J. Schmutz, S. B. Cannon, J. Schlueter, J. Ma, T. Mitros, W. Nelson, D. L. Hyten, Q. Song, J. J. Thelen, J. Cheng, D. Xu, U. Hellsten, G. D. May, Y. Yu, T. Sakurai, T. Umezawa, M. K. Bhattacharyya, D. Sandhu, B. Valliyodan, E. Lindquist, M. Peto, D. Grant, S. Shu, D. Goodstein, K. Barry, M. Futrell-Griggs, B. Abernathy, J. Du, Z. Tian, L. Zhu, N. Gill, T. Joshi, M. Libault, A. Sethuraman, X.-C. Zhang, K. Shinozaki, H. T. Nguyen, R. A. Wing, P. Cregan, J. Specht, J. Grimwood, D. Rokhsar, G. Stacey, R. C. Shoemaker, S. A. Jackson, Genome sequence of the palaeopolyploid soybean. Nature 463, 178–183 (2010). 20075913 10.1038/nature08670

[143] N. Kamal, T. Mun, D. Reid, J.-S. Lin, T. Y. Akyol, N. Sandal, T. Asp, H. Hirakawa, J. Stougaard, K. F. X. Mayer, S. Sato, S. U. Andersen, Insights into the evolution of symbiosis gene copy number and distribution from a chromosome-scale *Lotus japonicus* Gifu genome sequence. DNA Res. 27, (2020), 10.1093/dnares/dsaa015. PMC7508351 32658273

[144] N. Fernandez-Pozo, N. Menda, J. D. Edwards, S. Saha, I. Y. Tecle, S. R. Strickler, A. Bombarely, T. Fisher-York, A. Pujar, H. Foerster, A. Yan, L. A. Mueller, The Sol Genomics Network (SGN)—From genotype to phenotype to breeding. Nucleic Acids Res. 43, D1036–D1041 (2015). 25428362 10.1093/nar/gku1195 PMC4383978

[145] Tomato Genome Consortium, The tomato genome sequence provides insights into fleshy fruit evolution. Nature 485, 635–641 (2012). 22660326 10.1038/nature11119 PMC3378239

[146] G. M. Pham, J. P. Hamilton, J. C. Wood, J. T. Burke, H. Zhao, B. Vaillancourt, S. Ou, J. Jiang, C. R. Buell, Construction of a chromosome-scale long-read reference genome assembly for potato. Gigascience 9, giaa100 (2020). 32964225 10.1093/gigascience/giaa100 PMC7509475

[147] P. Lamesch, T. Z. Berardini, D. Li, D. Swarbreck, C. Wilks, R. Sasidharan, R. Muller, K. Dreher, D. L. Alexander, M. Garcia-Hernandez, A. S. Karthikeyan, C. H. Lee, W. D. Nelson, L. Ploetz, S. Singh, A. Wensel, E. Huala, The Arabidopsis Information Resource (TAIR): Improved gene annotation and new tools. Nucleic Acids Res. 40, D1202–D1210 (2012). 22140109 10.1093/nar/gkr1090 PMC3245047

[148] X. Argout, J. Salse, J.-M. Aury, M. J. Guiltinan, G. Droc, J. Gouzy, M. Allegre, C. Chaparro, T. Legavre, S. N. Maximova, M. Abrouk, F. Murat, O. Fouet, J. Poulain, M. Ruiz, Y. Roguet, M. Rodier-Goud, J. F. Barbosa-Neto, F. Sabot, D. Kudrna, J. S. S. Ammiraju, S. C. Schuster, J. E. Carlson, E. Sallet, T. Schiex, A. Dievart, M. Kramer, L. Gelley, Z. Shi, A. Bérard, C. Viot, M. Boccara, A. M. Risterucci, V. Guignon, X. Sabau, M. J. Axtell, Z. Ma, Y. Zhang, S. Brown, M. Bourge, W. Golser, X. Song, D. Clement, R. Rivallan, M. Tahi, J. M. Akaza, B. Pitollat, K. Gramacho, A. D’Hont, D. Brunel, D. Infante, I. Kebe, P. Costet, R. Wing, W. R. McCombie, E. Guiderdoni, F. Quetier, O. Panaud, P. Wincker, S. Bocs, C. Lanaud, The genome of *Theobroma cacao*. Nat. Genet. 43, 101–108 (2011). 21186351 10.1038/ng.736

[149] R. Ming, S. Hou, Y. Feng, Q. Yu, A. Dionne-Laporte, J. H. Saw, P. Senin, W. Wang, B. V. Ly, K. L. T. Lewis, S. L. Salzberg, L. Feng, M. R. Jones, R. L. Skelton, J. E. Murray, C. Chen, W. Qian, J. Shen, P. Du, M. Eustice, E. Tong, H. Tang, E. Lyons, R. E. Paull, T. P. Michael, K. Wall, D. W. Rice, H. Albert, M.-L. Wang, Y. J. Zhu, M. Schatz, N. Nagarajan, R. A. Acob, P. Guan, A. Blas, C. M. Wai, C. M. Ackerman, Y. Ren, C. Liu, J. Wang, J. Wang, J.-K. Na, E. V. Shakirov, B. Haas, J. Thimmapuram, D. Nelson, X. Wang, J. E. Bowers, A. R. Gschwend, A. L. Delcher, R. Singh, J. Y. Suzuki, S. Tripathi, K. Neupane, H. Wei, B. Irikura, M. Paidi, N. Jiang, W. Zhang, G. Presting, A. Windsor, R. Navajas-Pérez, M. J. Torres, F. A. Feltus, B. Porter, Y. Li, A. M. Burroughs, M.-C. Luo, L. Liu, D. A. Christopher, S. M. Mount, P. H. Moore, T. Sugimura, J. Jiang, M. A. Schuler, V. Friedman, T. Mitchell-Olds, D. E. Shippen, C. W. dePamphilis, J. D. Palmer, M. Freeling, A. H. Paterson, D. Gonsalves, L. Wang, M. Alam, The draft genome of the transgenic tropical fruit tree papaya (*Carica papaya* Linnaeus). Nature 452, 991–996 (2008). 18432245 10.1038/nature06856 PMC2836516

[150] G. A. Wu, S. Prochnik, J. Jenkins, J. Salse, U. Hellsten, F. Murat, X. Perrier, M. Ruiz, S. Scalabrin, J. Terol, M. A. Takita, K. Labadie, J. Poulain, A. Couloux, K. Jabbari, F. Cattonaro, C. Del Fabbro, S. Pinosio, A. Zuccolo, J. Chapman, J. Grimwood, F. R. Tadeo, L. H. Estornell, J. V. Muñoz-Sanz, V. Ibanez, A. Herrero-Ortega, P. Aleza, J. Pérez-Pérez, D. Ramón, D. Brunel, F. Luro, C. Chen, W. G. Farmerie, B. Desany, C. Kodira, M. Mohiuddin, T. Harkins, K. Fredrikson, P. Burns, A. Lomsadze, M. Borodovsky, G. Reforgiato, J. Freitas-Astúa, F. Quetier, L. Navarro, M. Roose, P. Wincker, J. Schmutz, M. Morgante, M. A. Machado, M. Talon, O. Jaillon, P. Ollitrault, F. Gmitter, D. Rokhsar, Sequencing of diverse mandarin, pummelo and orange genomes reveals complex history of admixture during citrus domestication. Nat. Biotechnol. 32, 656–662 (2014). 24908277 10.1038/nbt.2906 PMC4113729

