# Supplementary material for: Lotus japonicus VIH2 is an inositol pyrophosphate synthase that regulates arbuscular mycorrhiza
Source: Sci Adv. 2026 May 22;12(21):eaec5607. doi: 10.1126/sciadv.aec5607 (PMC13196753; doi:10.1126/sciadv.aec5607)
Supplement: Supplementary file 1 — Figs. S1 to S18 Tables S1 to S4 References [file sciadv.aec5607_sm.pdf]

Science Advances
AAAS

Supplementary Materials for

Lotus japonicus VIH2 is an inositol pyrophosphate synthase that regulates arbuscular mycorrhiza

Kiran Raj *et al.*

Corresponding author: Martina K. Ried-Lasi, martina.ried@ipb-halle.de;
Gabriel Schaaf, gabriel.schaaf@uni-bonn.de

Sci. Adv. **12**, eaec5607 (2026)
DOI: 10.1126/sciadv.aec5607

This PDF file includes:

Figs. S1 to S18
Tables S1 to S4
References

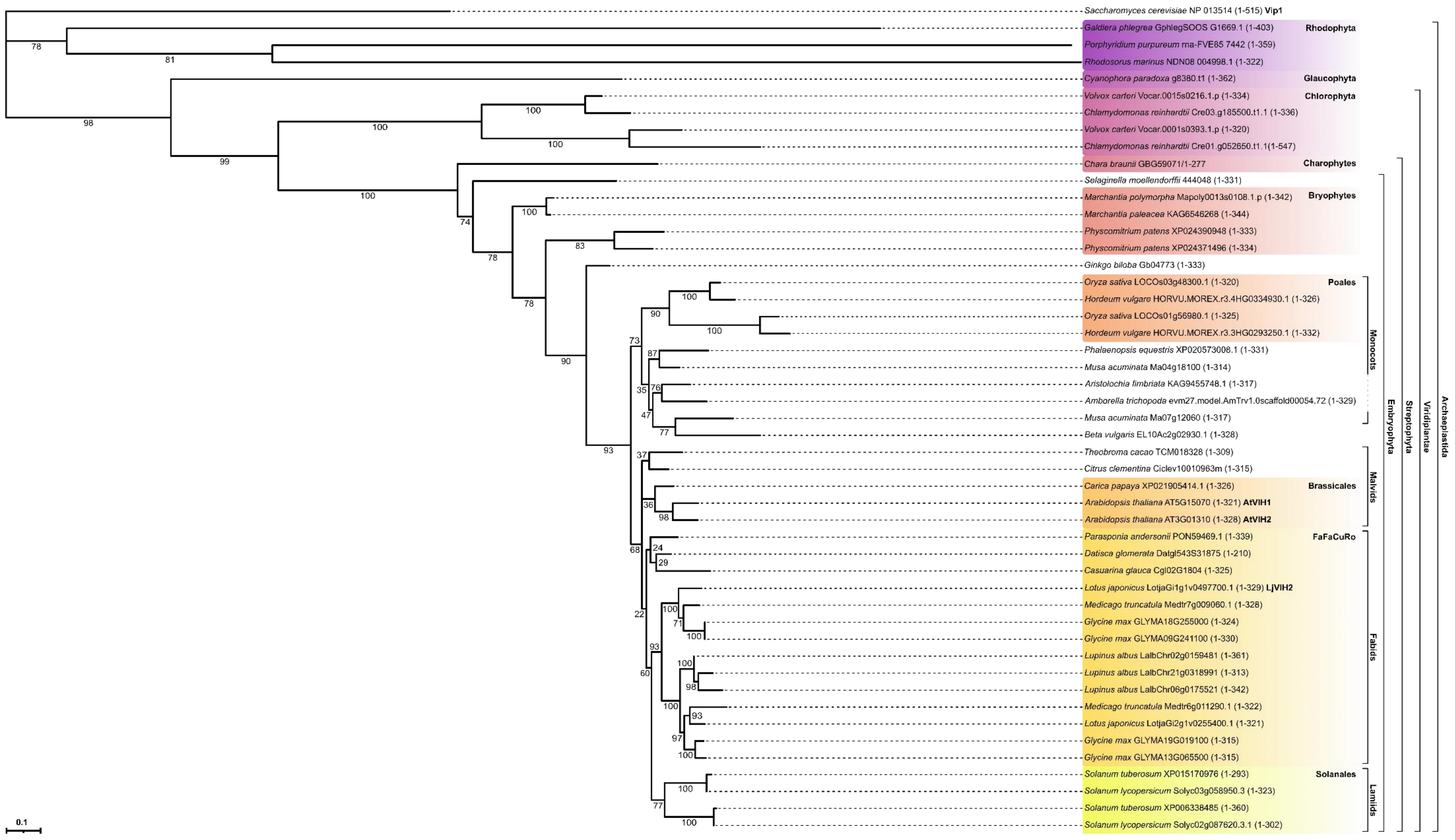
Saccharomyces cerevisiae NP 013514 (1-515) Vip1
Galdieria phlegrea GphlegSOOS G1669.1 (1-403)
Porphyridium purpureum rna-FVE85 7442 (1-359)
Rhodosorus marinus NDN08 004998.1 (1-322)
Rhodophyta
Cyanophora paradoxa g8380.t1 (1-362)
Glaucophyta
Volvox carteri Vocar.0015s0216.1.p (1-334)
Chlamydomonas reinhardtii Cre03.g185500.t1.1 (1-336)
Volvox carteri Vocar.0001s0393.1.p (1-320)
Chlamydomonas reinhardtii Cre01.g052650.t1.1(1-547)
Chlorophyta
Chara braunii GBG59071/1-277
Charophytes
Selaginella moellendorffii 444048 (1-331)
Marchantia polymorpha Mapoly0013s0108.1.p (1-342)
Marchantia paleacea KAG6546268 (1-344)
Physcomitrium patens XP024390948 (1-333)
Physcomitrium patens XP024371496 (1-334)
Bryophytes
Ginkgo biloba Gb04773 (1-333)
Oryza sativa LOCOs03g48300.1 (1-320)
Hordeum vulgare HORVU.MOREX.r3.4HG0334930.1 (1-326)
Oryza sativa LOCOs01g56980.1 (1-325)
Hordeum vulgare HORVU.MOREX.r3.3HG0293250.1 (1-332)
Poales
Phalaenopsis equestris XP020573008.1 (1-331)
Musa acuminata Ma04g18100 (1-314)
Aristolochia fimbriata KAG9455748.1 (1-317)
Amborella trichopoda evm27.model.AmTrv1.0scaffold00054.72 (1-329)
Musa acuminata Ma07g12060 (1-317)
Beta vulgaris EL10Ac2g02930.1 (1-328)
Monocots
Theobroma cacao TCM018328 (1-309)
Citrus clementina Ciclev10010963m (1-315)
Carica papaya XP021905414.1 (1-326)
Arabidopsis thaliana AT5G15070 (1-321) AtVIH1
Arabidopsis thaliana AT3G01310 (1-328) AtVIH2
Brassicales
Malvids
Parasponia andersonii PON59469.1 (1-339)
Datisca glomerata Dalgl543S31875 (1-210)
Casuarina glauca Cgl02G1804 (1-325)
FaFaCuRo
Lotus japonicus LotjaGi1g1v0497700.1 (1-329) LjVIH2
Medicago truncatula Medtr7g009060.1 (1-328)
Glycine max GLYMA18G255000 (1-324)
Glycine max GLYMA09G241100 (1-330)
Lupinus albus LalbChr02g0159481 (1-361)
Lupinus albus LalbChr21g0318991 (1-313)
Lupinus albus LalbChr06g0175521 (1-342)
Medicago truncatula Medtr6g011290.1 (1-322)
Lotus japonicus LotjaGi2g1v0255400.1 (1-321)
Glycine max GLYMA19G019100 (1-315)
Glycine max GLYMA13G065500 (1-315)
Fabids
Solanum tuberosum XP015170976 (1-293)
Solanum lycopersicum Solyc03g058950.3 (1-323)
Solanum tuberosum XP006338485 (1-360)
Solanum lycopersicum Solyc02g087620.3.1 (1-302)
Solanales
Lamiids
Embryophyta
Streptophyta
Viridiplantae
Archaeplastida
0.1

Fig. S1. Phylogenetic analysis of VIH2 homologs in algae and land plants. A maximum-likelihood tree was constructed based on the amino acid sequence alignment of the ATP-grasp kinase domains of VIH2 homologs, using the Saccharomyces cerevisiae Vip1 ATP-grasp kinase domain as query. ScVip1 was used as an outgroup. Numbers on each node represent the respective bootstrap values. Bootstrap values below 20 were omitted. Branch lengths are proportional to the number of amino acid substitutions per site. Species names and unique protein identifiers are indicated. Amino acid residues used for the alignment are stated in brackets. A. fimbriata and A. trichopoda are not included in the monocots (dashed line). FaFaCuRo, Nitrogen-fixing nodulating clade (Fabales, Fagales, Cucurbitales, Rosales); Bar, average number of substitutions per site.

Exon 1 short

ATG ACG GCG ACG GTG GAG GAA GAA GCG GTT GCG GTG AAG AAG ATA ACG ATT GGA GTC TGC GTG ATG GAA AAG AAG
M T A T V E E E A V A V K K I T I G V C V M E K K

Exon 1 long

GTG AAA TGT GGC TCC GAG gtcctctctcttatactttttcacgctctggatccactgttttgctactcgcgagtccagcgagttgctatgaatg
V K C G S E

Exon2

atcactctgttttgaatcgc GTT TCA TCG GCG CCT ATG GAG CAG ATT CTT CGC CGT TTA CAA GCA TTT GGT GAA TTT GAG
V S S A P M E Q I L R R L Q A F G E F E

Fig. S2. *Lotus japonicus* VIH2 splice variants. The nucleotide sequence of the 5’ genomic region (positions 1–261) of *L. japonicus VIH2* is shown, corresponding to the start codon and N-terminal coding sequence with intron/exon boundaries, and the putative amino acid sequence is stated below. Capital letters indicate exons while small letters indicate the intron. The short version of exon 1 and exon 2 are highlighted in cyan, while the long version of exon 1 is encircled in yellow. Note that only this portion of the gene is shown; the full gene sequence is considerably longer. Both splice variants were detected in our cDNA preparations and were therefore analysed functionally.

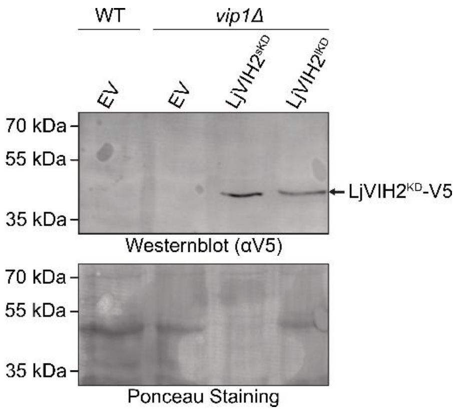

Fig. S3. ***Lotus japonicus*** **VIH2 is expressed in the** ***Saccharomyces cerevisiae vip1*****Δ knock-out mutant strain.** The *S. cerevisiae* wildtype or *vip1*Δ mutant strain was transformed with the episomal pAG426GPD-*ccdB* empty vector (EV) or with plasmids carrying sequences encoding either a short (LjVIH2^{sKD}) or a long (LjVIH2^{lKD}) version of the isolated *L. japonicus* VIH2 kinase domain fused to a C-terminal V5-tag. Gal4 served as loading control.

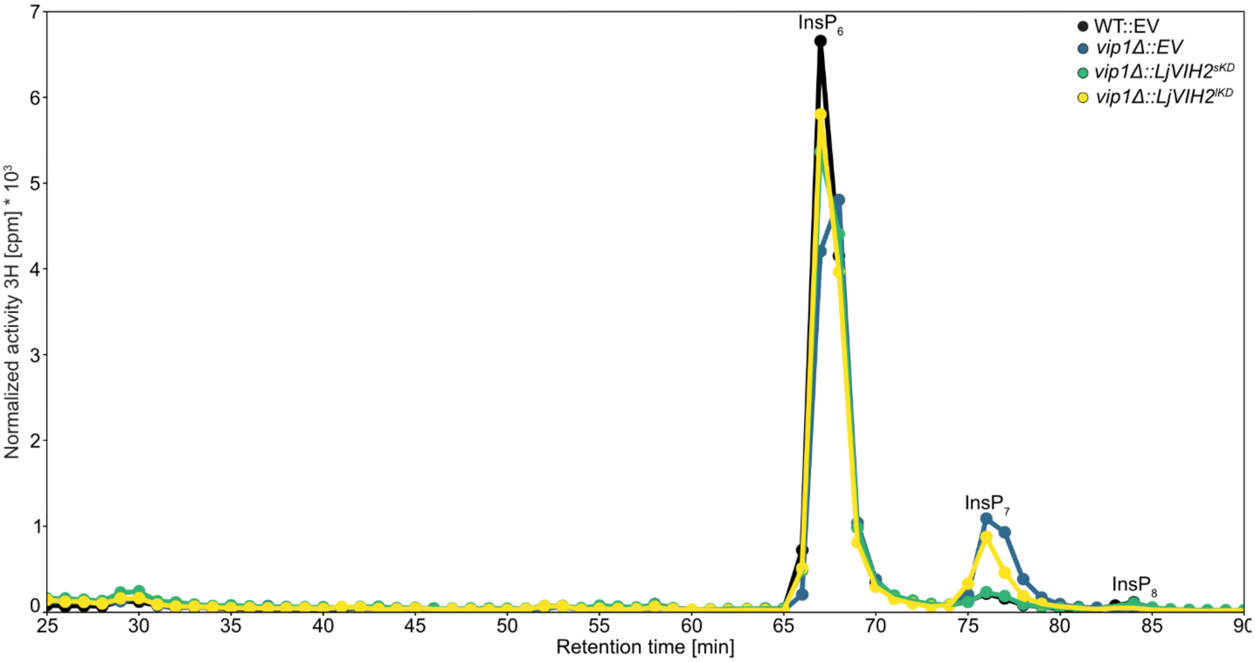

Fig. S4. *Lotus japonicus* VIH2 is a functional Vip1-type PP-InsP synthase. Normalized HPLC profiles of InsPs of extracts from [^{3}H]-*myo*-inositol-labelled *S. cerevisiae* BY4741 wildtype (WT) or a *vip1Δ* mutant strain transformants either carrying the empty vector (EV) or ectopically expressing a short (LjVIH2^{sKD}) or a long (LjVIH2^{lKD}) splice variant of the isolated *L. japonicus* VIH2 kinase domain. Extracts were resolved by SAX-HPLC and fractions were collected each minute for subsequent determination of radioactivity.

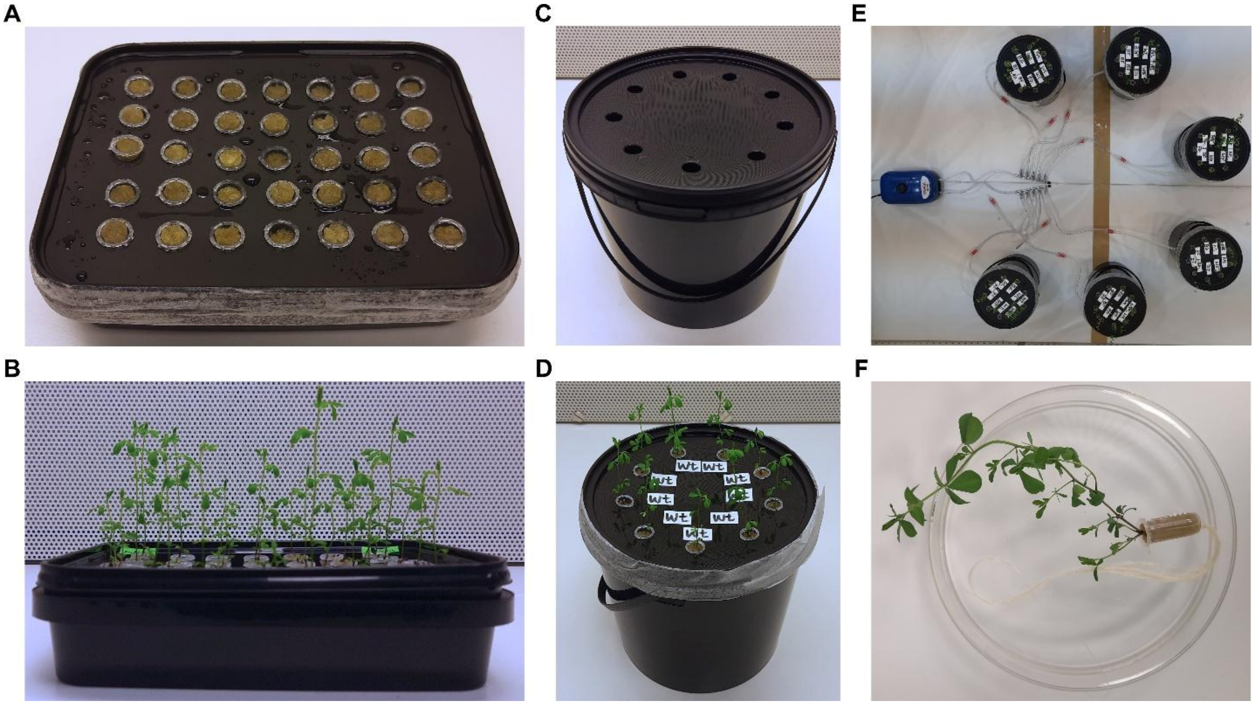

Fig. S5. A hydroponic cultivation system to study PSRs and InsP signalling in *Lotus japonicus*. A+B, *L. japonicus* seeds were germinated in cut-open 1.5 mL reaction tubes filled with rockwool and placed into flat black boxes (bikapak; Logiflex lid black, 00202201; Logiflex 660 mL box black, 0202233) containing 250 mL liquid Lotus cultivation medium. **C+D,** Once the root systems reached the desired length, plants were transferred to black 2.5 L buckets (bikapak; round bucket 2.5 L black, 00201989; lid for round bucket 2.5 L black, 00201899) containing 1.8 L liquid Lotus cultivation medium. **E,** Buckets were connected to an air pump (OSAGA, MK-9502) to ensure continuous aeration. **F,** Reaction tubes were removed from the bucket lids for harvesting.

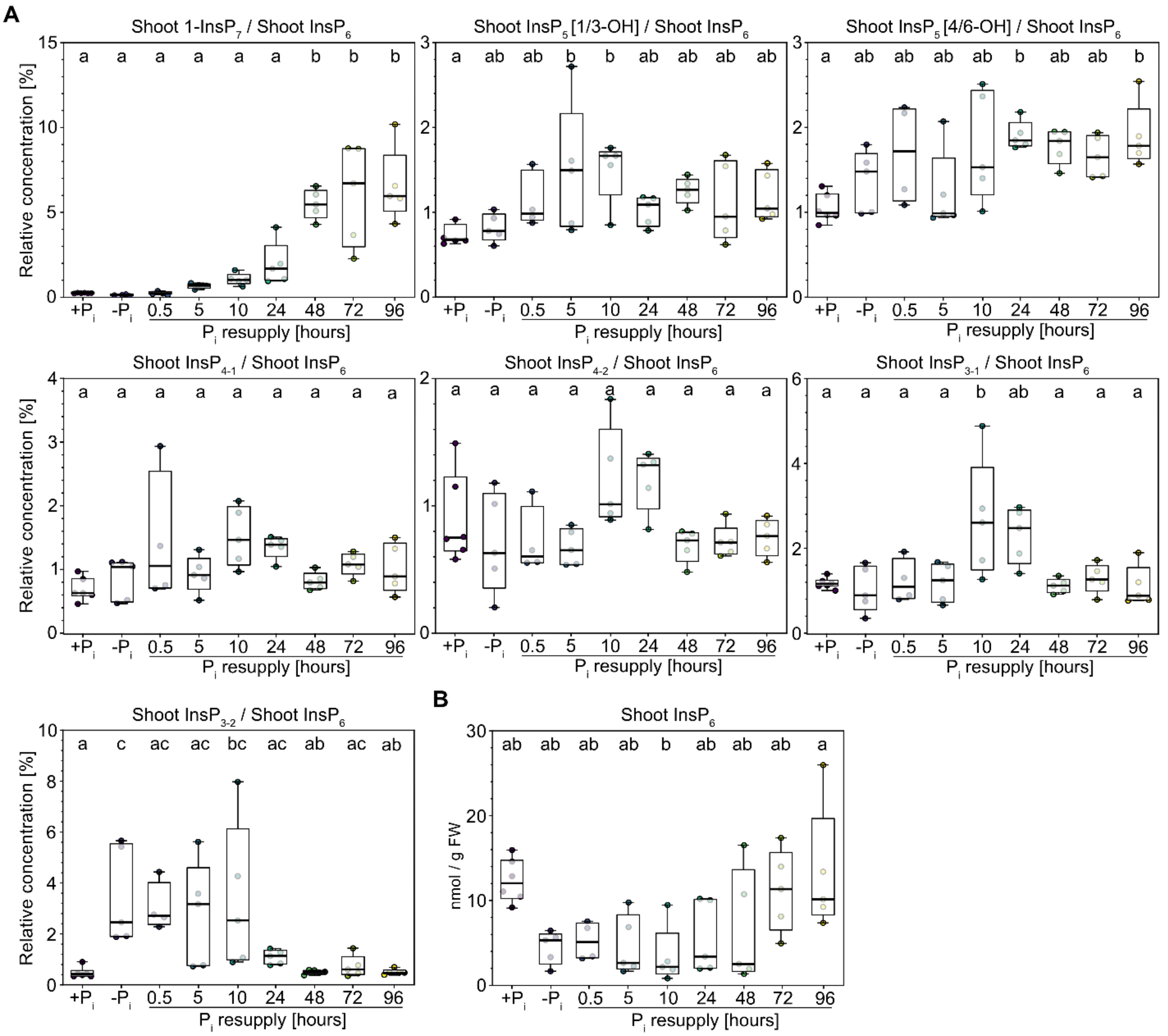

Fig. S6. Changes in shoot InsP levels in hydroponically grown *Lotus japonicus* upon phosphate starvation and resupply. *L. japonicus* wildtype seeds were germinated and seedlings were grown in +P_i liquid medium containing 1500 µM P_i for four weeks. Subsequently, plants were transferred to -P_i liquid medium, starved for 8 days (-P_i), followed by transfer back to +P_i liquid medium for P_i resupply for 0.5 to 96 hours (P_i resupply). Shoot InsPs were enriched by TiO_2 pull-down and quantified *via* CE-ESI-MS analysis. **A,** Shoot InsP levels are presented relative to shoot $InsP_6$. $InsP_{4-1}$ contains 1,4,5,6-$InsP_4$, whereas $InsP_{4-2}$ contains 2,3,4,5-$InsP_4$ but the potential presence of undefined isomers cannot be excluded. $InsP_{3-1}$ represents an inseparable mixture that likely includes 1,2,3-$InsP_3$, 3,4,5-$InsP_3$, and 1,2,6-$InsP_3$, whereas $InsP_{3-2}$ comprises an inseparable mixture potentially containing 1,3,4-$InsP_3$, 1,4,5-$InsP_3$, and 1,4,6-$InsP_3$. **B,** Shoot $InsP_6$ levels. n = 4–5. For statistical analysis, an ordinary one-way ANOVA with Tukey's multiple comparisons test was performed. Different letters indicate significant differences ($p \leq 0.05$).

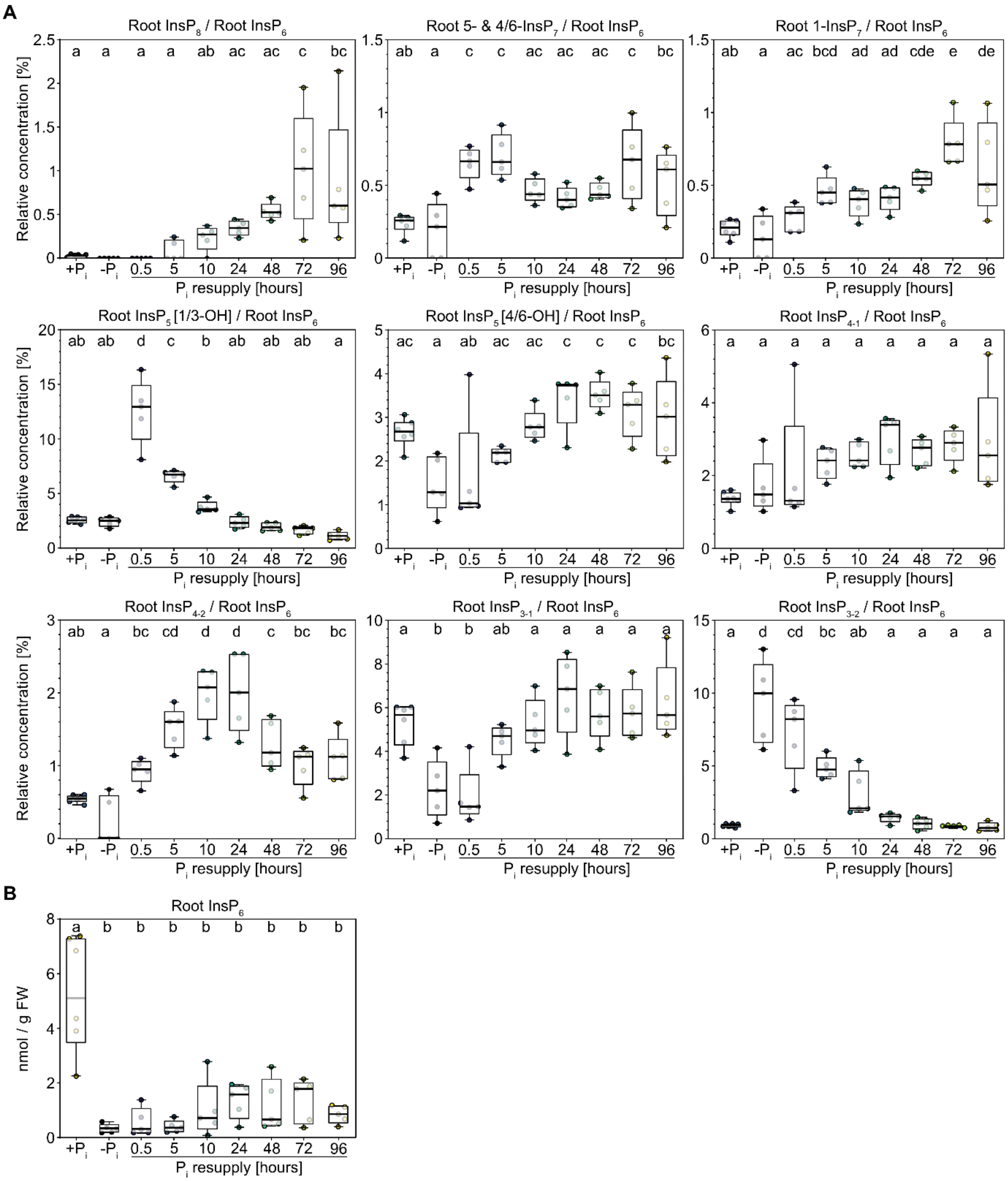

Fig. S7. Changes in root InsP levels in hydroponically grown *Lotus japonicus* upon phosphate starvation and resupply. *L. japonicus* wildtype seeds were germinated and seedlings were grown in +P_i liquid medium containing 1500 µM P_i for four weeks. Subsequently, plants were transferred to -P_i liquid medium, starved for 8 days (-P_i), followed by transfer back to +P_i liquid medium for P_i resupply for 0.5 to 96 hours (P_i resupply). Root InsPs were enriched by TiO_2 pull-down and quantified *via* CE-ESI-MS analysis. **A,** Root InsP levels are presented relative to root $InsP_6$. $InsP_{4-1}$ contains 1,4,5,6-$InsP_4$, whereas $InsP_{4-2}$ contains 2,3,4,5-$InsP_4$ but the potential presence of undefined isomers cannot be excluded $InsP_{3-1}$ represents an inseparable mixture that likely

includes 1,2,3-$InsP_3$, 3,4,5-$InsP_3$, and 1,2,6-$InsP_3$, whereas $InsP_{3\text{-}2}$ comprises an inseparable mixture potentially containing 1,3,4-$InsP_3$, 1,4,5-$InsP_3$, and 1,4,6-$InsP_3$. **B,** Root $InsP_6$ levels. $n = 5$. For statistical analysis, an ordinary one-way ANOVA with Tukey's multiple comparisons test was performed. Different letters indicate significant differences ($p \leq 0.05$).

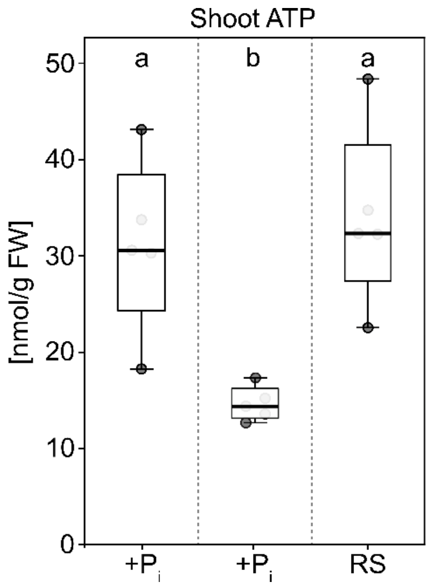

Fig. S8. Changes in shoot ATP levels in hydroponically grown *Lotus japonicus* upon phosphate starvation and resupply. *L. japonicus* wildtype seeds were germinated and seedlings were grown in +P_i liquid medium containing 1500 µM P_i for four weeks. Subsequently, plants were transferred to -P_i liquid medium, starved for 8 days (-P_i), followed by transfer back to +P_i liquid medium for P_i resupply for 72 hours (RS). Shoot ATP was enriched by Nb_2O_5 pull-down and quantified *via* CE-ESI-MS analysis. Shoot ATP levels are presented. n = 5. For statistical analysis, an ordinary one-way ANOVA with Tukey's multiple comparisons test was performed. Different letters indicate significant differences ($p \leq 0.05$).

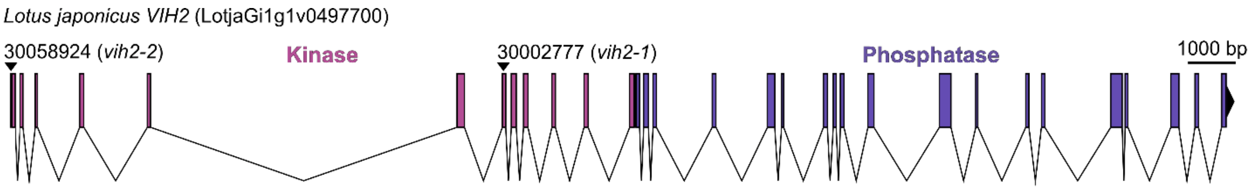

Fig. S9. ***Lotus japonicus VIH2*** **LORE1 retrotransposon insertion lines.** Visual representation of the intron-exon structure of *L. japonicus VIH2*. LORE1 retrotransposon insertions in the *vih2-1* and *vih2-2* mutant lines and LORE1 line identifiers are stated. Pink, kinase domain; lilac, phosphatase domain. The figure was created with the help of Exon-Intron Graphic Maker (http://www.wormweb.org/exonintron; Nikhil Bhatla). Bar, 1000 bp.

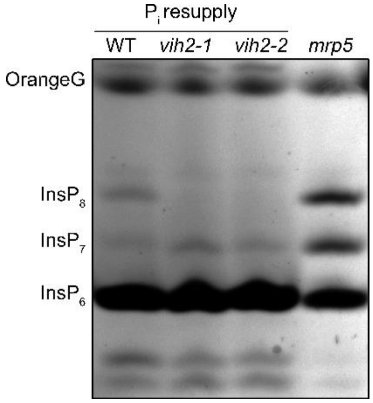

Fig. S10. ***Lotus japonicus vih2*** **mutants have altered PP-InsP levels.** *L. japonicus* wildtype (WT) and *vih2* mutant seeds were germinated and seedlings were grown in +P_i liquid medium containing 1500 µM P_i for four weeks. Subsequently, plants were transferred to -P_i liquid medium, starved for 10 days, followed by transfer back to +P_i liquid medium for P_i resupply for 72 hours (P_i resupply). Root InsPs were enriched by Nb_2O_5 pull-down, mixed with a loading buffer containing OrG as a loading dye, and resolved by by PAGE for qualitative illustration of InsP profiles and visualized by toluidine blue and subsequent DAPI staining. TiO2-purified Arabidopsis *mrp5* seed extracts, which accumulate high amounts of $InsP_7$ and $InsP_8$ were used as a marker for $InsP_6$, $InsP_7$ and $InsP_8$.

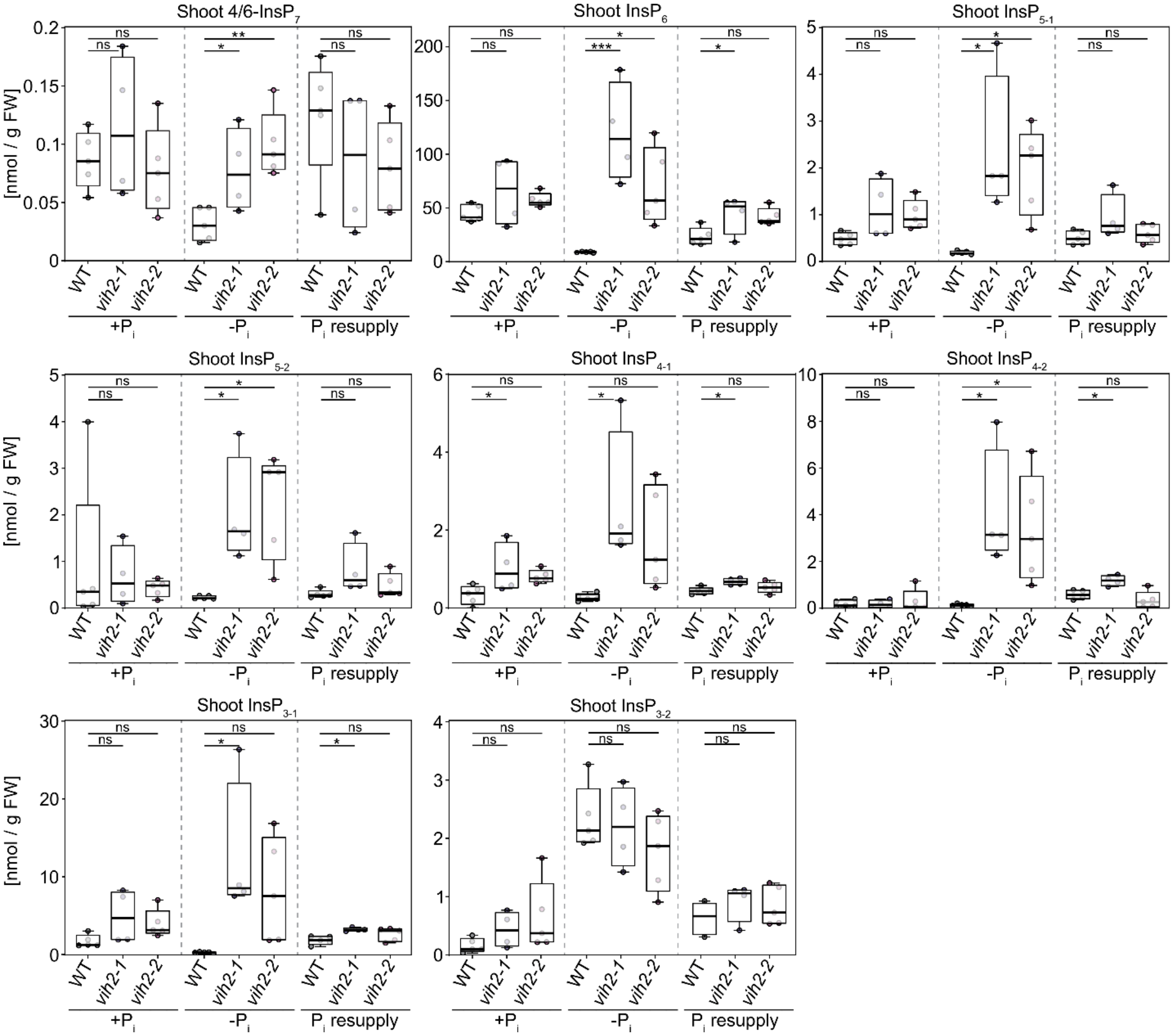

Fig. S11. *Lotus japonicus vih2* mutants have altered shoot InsP levels. *L. japonicus* wildtype (WT) and *vih2* mutant seeds were germinated and seedlings were grown in +P_i liquid medium containing 1500 µM P_i for four weeks. Subsequently, plants were either transferred to -P_i liquid medium, starved for 10 days (-P_i), followed by transfer back to +P_i liquid medium for P_i resupply for 72 hours (P_i resupply), or kept on +P_i liquid medium for the whole time (+P_i). Shoot InsPs were enriched by Nb_2O_5 pull-down and quantified *via* CE-ESI-MS analysis. $InsP_{5-1}$ may include $InsP_5$ [4/6-OH] and $InsP_5$ [5-OH], while $InsP_{5-2}$ refers to $InsP_5$ [2-OH] and $InsP_5$ [1/3-OH]. $InsP_{4-1}$ contains 1,4,5,6-$InsP_4$, whereas $InsP_{4-2}$ contains 2,3,4,5-$InsP_4$ but the potential presence of undefined isomers cannot be excluded. $InsP_{3-1}$ represents an inseparable mixture that likely includes 1,2,3-$InsP_3$, 3,4,5-$InsP_3$, and 1,2,6-$InsP_3$, whereas $InsP_{3-2}$ comprises an inseparable mixture potentially containing 1,3,4-$InsP_3$, 1,4,5-$InsP_3$, and 1,4,6-$InsP_3$. n = 4–5. For statistical analysis, an ordinary one-way ANOVA with Dunnett's multiple comparisons test was performed. *, $p \leq 0.05$; **, $p \leq 0.01$; ***, $p \leq 0.001$.

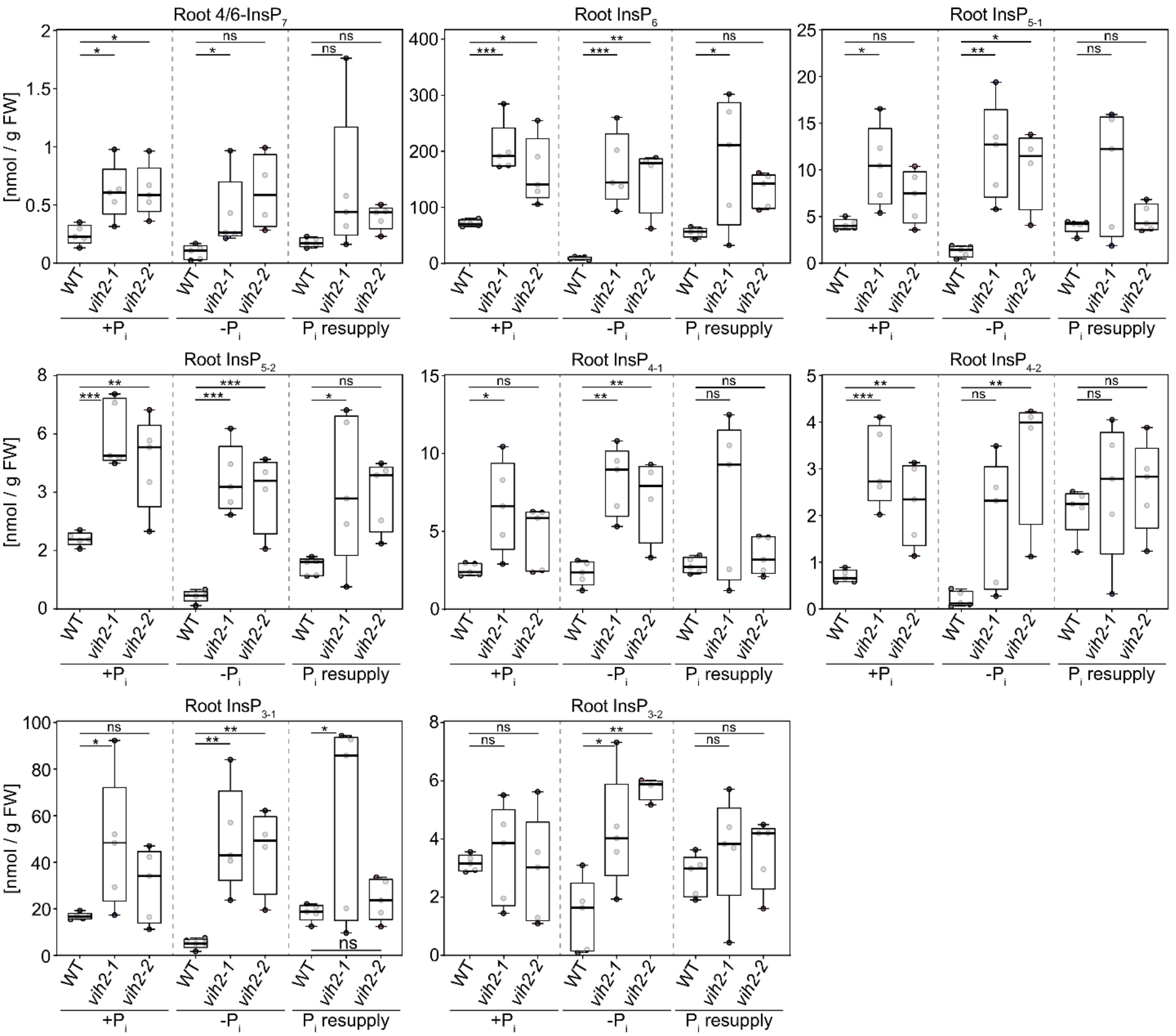

Fig. S12. *Lotus japonicus vih2* mutants have altered root InsP levels. *L. japonicus* wildtype (WT) and *vih2* mutant seeds were germinated and seedlings were grown in $+P_i$ liquid medium containing 1500 µM P_i for four weeks. Subsequently, plants were either transferred to $-P_i$ liquid medium, starved for 10 days ($-P_i$), followed by transfer back to $+P_i$ liquid medium for P_i resupply for 72 hours (P_i resupply), or kept on $+P_i$ liquid medium for the whole time ($+P_i$). Root InsPs were enriched by Nb_2O_5 pull-down and quantified *via* CE-ESI-MS analysis. $InsP_{5\text{-}1}$ may include $InsP_5$ [4/6-OH] and $InsP_5$ [5-OH], while $InsP_{5\text{-}2}$ refers to $InsP_5$ [2-OH] and $InsP_5$ [1/3-OH]. $InsP_{4\text{-}1}$ contains 1,4,5,6-$InsP_4$, whereas $InsP_{4\text{-}2}$ contains 2,3,4,5-$InsP_4$ but the potential presence of undefined isomers cannot be excluded. $InsP_{3\text{-}1}$ represents an inseparable mixture that likely includes 1,2,3-$InsP_3$, 3,4,5-$InsP_3$, and 1,2,6-$InsP_3$, whereas $InsP_{3\text{-}2}$ comprises an inseparable mixture potentially containing 1,3,4-$InsP_3$, 1,4,5-$InsP_3$, and 1,4,6-$InsP_3$. n = 4–5. For statistical analysis, an ordinary one-way ANOVA with Dunnett's multiple comparisons test was performed. *, $p \leq 0.05$; **, $p \leq 0.01$; ***, $p \leq 0.001$.

A

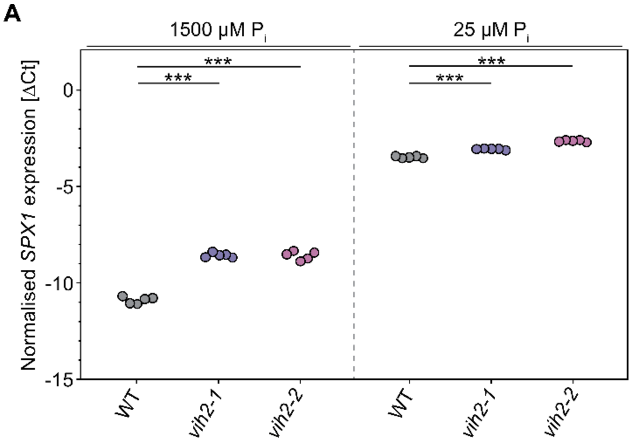

B

- 20% n. c. + 400%

Shoot P content relative to WT (25 µM P_i)

	25 µM P_i	250 µM P_i	750 µM P_i	1500 µM P_i	2500 µM P_i
WT	100	112	238	300	320
vih2-1	95	129 **	273	428 ***	493 ***
vih2-2	86	102	220	401 ***	427 **

Fig. S13. *Lotus japonicus vih2* mutants show enhanced, constitutive PSRs. Ten-day-old *L. japonicus* wildtype (WT) and *vih2* mutant seedlings were planted in open pots containing 300 mL washed sand (5 seedlings per pot). Plants were fertilized once a week with liquid Lotus cultivation medium containing 25–2500 µM P_i and harvested five weeks after planting. **A,** The expression level of the PSI marker gene *SPX1* was analysed in roots *via* qRT-PCR. n = 5. **B,** Shoot P levels were determined *via* ICP-OES analysis. Data is presented relative to P levels in wildtype shoots grown in the presence of 25 µM P_i, which were set to 100 %. n = 4–10. Numbers indicate percentages. Stars indicate significant differences of the respective *vih2* mutant line in comparison to the corresponding wildtype. Note: The shoot P data shown here and the data presented in Fig. 4D originate from the same ICP–OES dataset and are displayed in both figures for comparative purposes. **A+B,** For statistical analysis, an ordinary one-way ANOVA with Dunnett's multiple comparisons test was performed. **, $p \leq 0.01$; ***, $p \leq 0.001$.

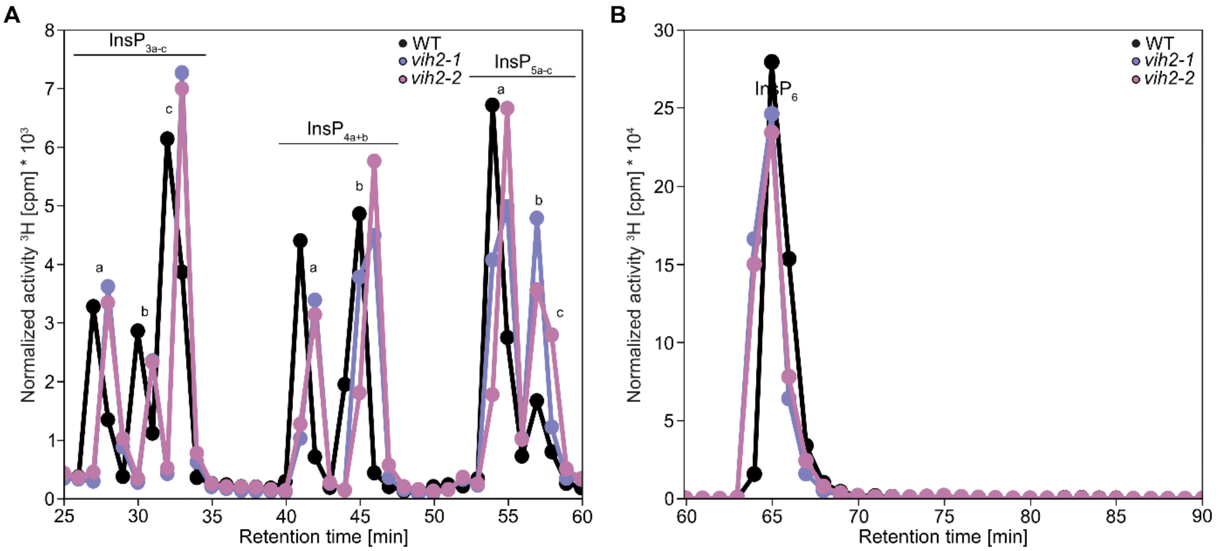

Fig. S14. *Lotus japonicus vih2* mutants have altered (PP)-InsP levels. *L. japonicus* wildtype (WT) and *vih2* mutant seeds were germinated and subsequently grown for 14 days on water agar plates before transfer to liquid ½ MS media supplemented with 1 % sucrose and 45 µCi of [^{3}H]-*myo*-inositol for 5 days. InsPs were extracted and separated by SAX-HPLC. Data for retention times of **A,** 25–60 minutes and **B,** 60–90 minutes are plotted separately. $InsP_{4a}$ likely refers to Ins(1,4,5,6)P_4 or Ins(3,4,5,6)P_4, $InsP_{5a}$ refers to $InsP_5$ [2-OH], $InsP_{5b}$ refers to $InsP_5$ [4-OH] or its enantiomeric form $InsP_5$ [6-OH], and $InsP_{5c}$ refers to $InsP_5$ [1-OH] or its enantiomeric form $InsP_5$ [3-OH]. The isomeric natures of $InsP_{3a\text{-}c}$ and $InsP_{4b}$ are still unknown. cpm, counts per minute.

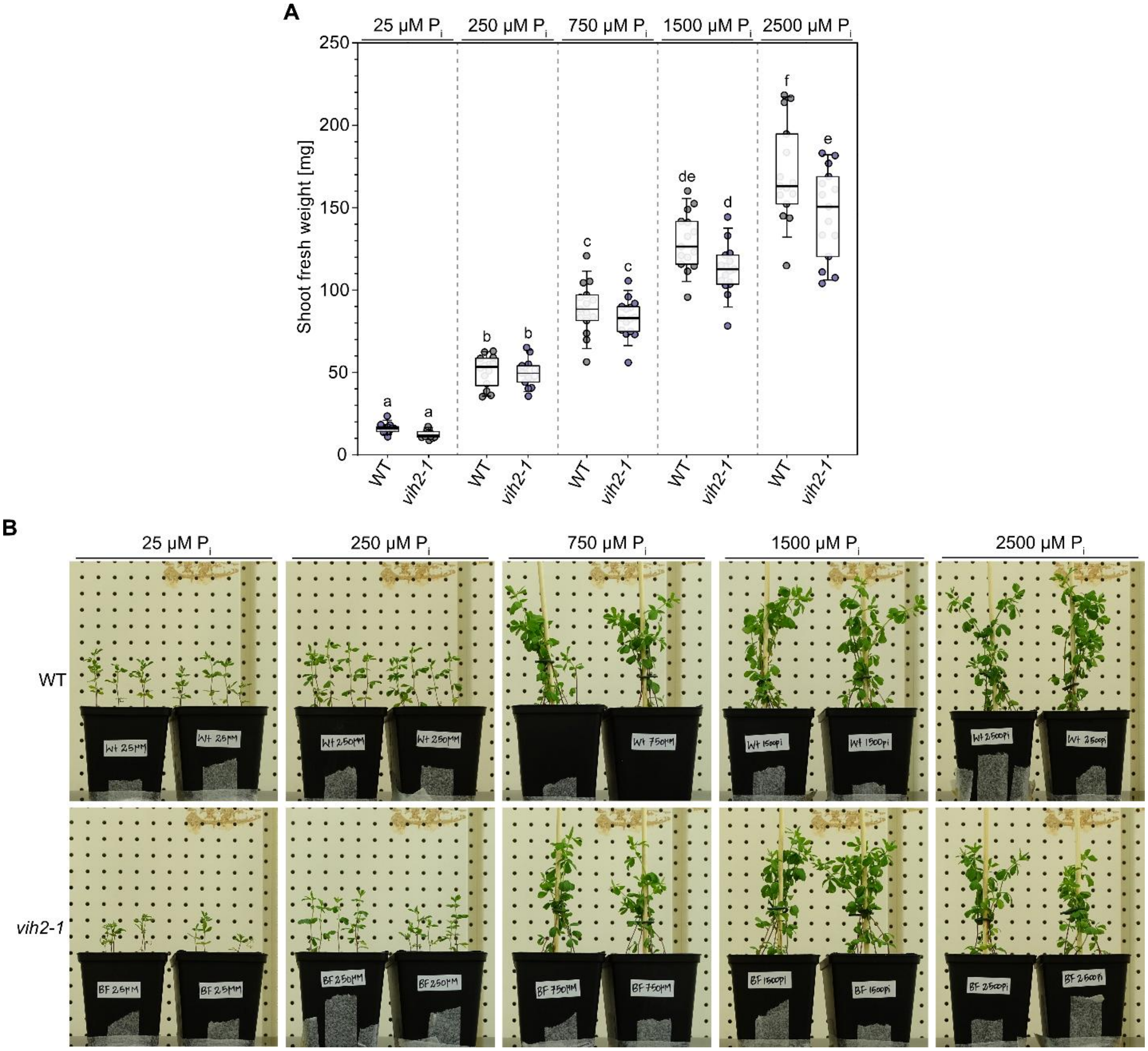

Fig. S15. *Lotus japonicus vih2* mutants do not show strong growth defects. Ten-day-old *L. japonicus* wildtype (WT) and *vih2* mutant seedlings were planted in open pots containing 300 mL washed sand (5 seedlings per pot). Plants were fertilized once a week with liquid Lotus cultivation medium containing 25–2500 µM P_i. **A,** The shoot fresh weight per plant was measured 4.5 weeks post planting. n = 13-15. For statistical analysis, an ordinary one-way ANOVA with Tukey's multiple comparisons test was performed. Different letters indicate significant differences ($p \leq 0.5$). **B,** Representative pictures of plants analysed in **A,**.

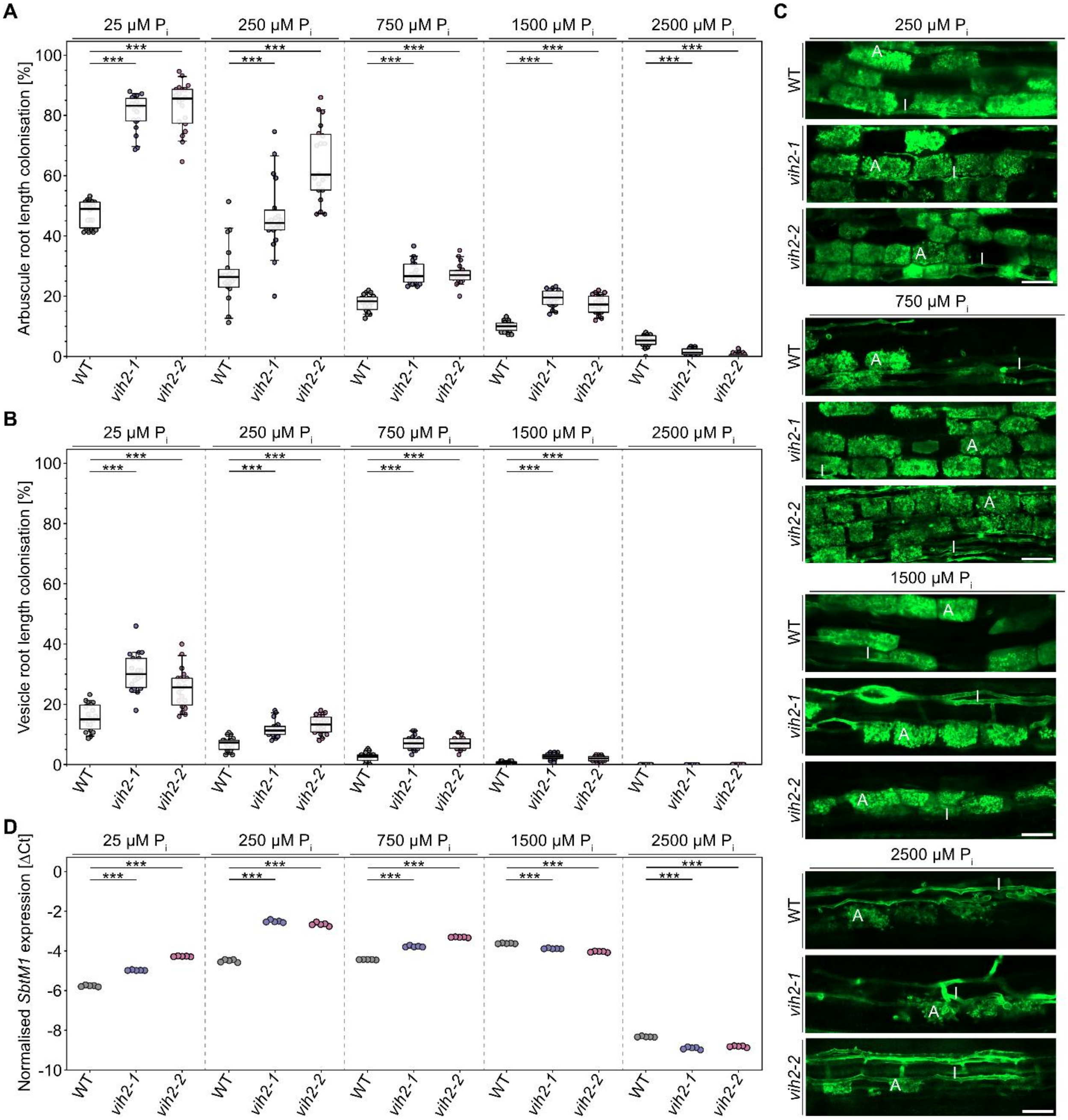

Fig. S16. *Lotus japonicus vih2* mutants are significantly better colonized by AM fungi than wildtype plants. Ten-day-old *L. japonicus* wildtype (WT) and *vih2* mutant seedlings were planted in open pots containing 300 mL washed sand (5 seedlings per pot) and inoculated with *R. irregularis* spores (Symplanta, Germany; 500 spores per plant). Plants were fertilized once a week with liquid Lotus cultivation medium containing the indicated P_i concentrations and harvested 4.5 weeks after planting. Roots were stained with ink-vinegar and arbuscules **A,** and vesicles **B,** were quantified. n = 20. **C,** Roots were stained with wheat germ agglutinin conjugated to Alexa Fluor 488 and observed with a confocal microscope. Representative pictures are shown. Bar, 50 µm; A, arbuscule; I, intraradical hypha. **D,** The expression level of the AM marker *SbtM1* was analyzed

in roots *via* qRT-PCR. n = 5. **A+B, D,** For statistical analysis, an ordinary one-way ANOVA with Dunnett's multiple comparisons test was performed. ***, $p \leq 0.001$.

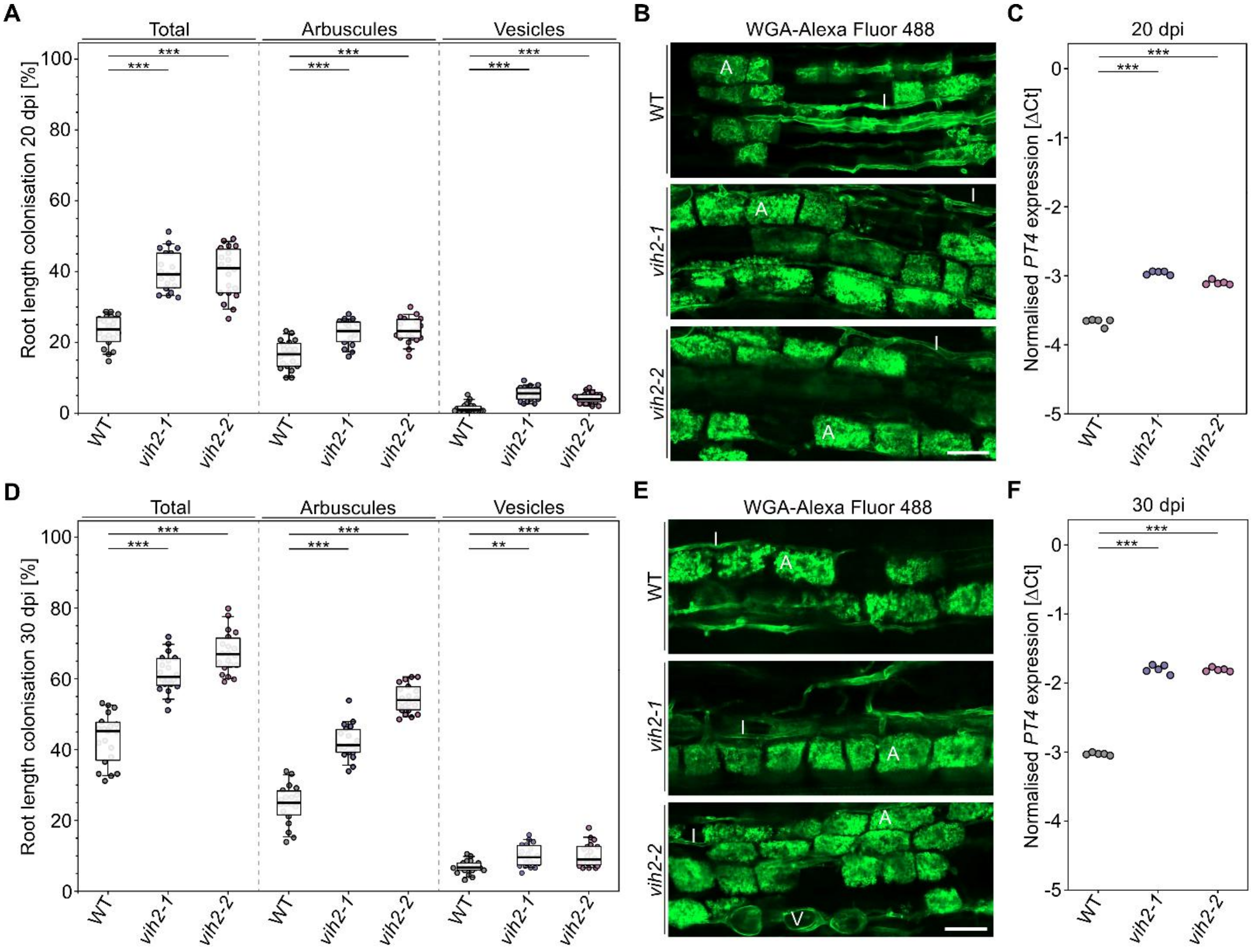

Fig. S17. *Lotus japonicus vih2* mutants are significantly better colonized by AM fungi than wildtype plants. Ten-day-old *L. japonicus* wildtype (WT) and *vih2* mutant seedlings were planted in open pots containing 300 mL washed sand (5 seedlings per pot) and grown in the presence of *R. irregularis* spore inoculum (Symplanta, Germany; 500 spores per plant). Plants were fertilized once a week with liquid Lotus cultivation medium containing 25 µM P_i harvested 20 **A-C,** or 30 dpi **D-F,** after planting. **A+D,** Roots were stained with ink-vinegar and AM colonization was quantified. n = 20. **B+E,** Roots were stained with wheat germ agglutinin (WGA) conjugated to Alexa Fluor 488 and observed with a confocal microscope. Representative pictures are shown. Bar, 50 µm; V, vesicles; A, arbuscule; I, intraradical hypha. **C+F,** The expression level of the AM marker gene *PT4* was analysed in roots *via* qRT-PCR. n = 5. **A, C, D, F,** For statistical analysis, an ordinary one-way ANOVA with Dunnett's multiple comparisons test was performed. **, p ≤ 0.01; ***, p ≤ 0.001.

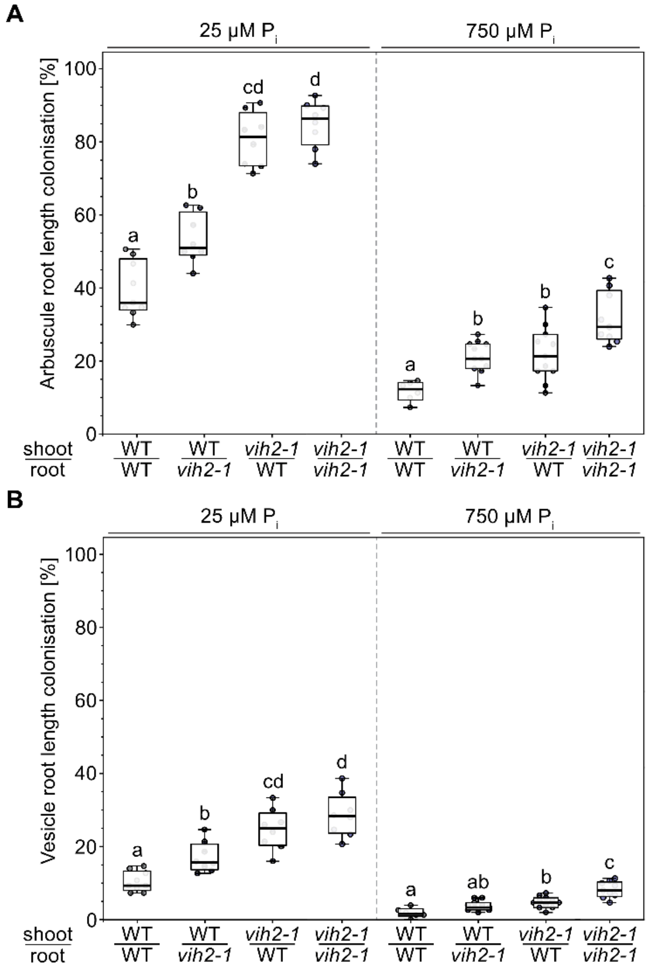

Fig. S18. Colonization of *Lotus japonicus* by AM fungi is controlled systemically and locally by (PP)-InsPs. Six-day-old *L. japonicus* wildtype (WT) and *vih2-1* mutant seedlings were self-grafted or reciprocally grafted and grown for three weeks on plates. Subsequently, successful grafts were planted in open pots containing 300 mL washed sand (5 seedlings per pot) and grown in the presence of *R. irregularis* spore inoculum (Symplanta, Germany; 500 spores per plant). Plants were fertilized once a week with liquid Lotus cultivation medium containing 25 or 750 µM P_i and harvested five weeks after planting. Roots were stained with ink-vinegar and arbuscules **A,** and vesicles **B,** were quantified. n = 6–11. For statistical analysis, an ordinary one-way ANOVA with Tukey's multiple comparisons test was performed. Different letters indicate significant differences ($p \leq 0.05$).

Table S1. Constructs. Detailed description of the vector backbones and constructs used in the present study produced by Gateway cloning. EV, empty vector control.

Name	Purpose	Cloning strategy	Reference
pAG426GPD-*ccdB*	Yeast expression, EV	Gateway	*(98)*
pAG426GPD-LjVIH2^{sKD}	Yeast expression	Gateway	This study
pAG426GPD-LjVIH2^{lKD}	Yeast expression	Gateway	This study
pET28-His_8-MBP	*E. coli* expression, EV	Classical cloning	*(26)*
pDEST566-His_6-MBP-LjVIH2^{sKD}	*E. coli* expression	Gateway	This study

Table S2. Primers. List of primers used in the present study.

Name	Sequence	Purpose	Reference
MR379_LjUbi_F	ATGCAGATCTTCGTCAAGACCTTG	qRT-PCR	*(11)*
MR380_LjUbi_R	ACCTCCCCTCAGACGAAG	qRT-PCR	*(11)*
MR783_LjSPX_F	GCAACAGATTCAGGAGACAC	qRT-PCR	*(114)*
MR784_LjSPX_R	CCTTACCATACTCCAACGAC	qRT-PCR	*(114)*
MR1190_LjPT4_F	GAATAAAGGGGCCAAAATCG	qRT-PCR	*(115)*
MR1191_LjPT4_R	GCTGTATCCTATCCCCATGC	qRT-PCR	*(115)*
MR1194_LjSbtM1_F	CACGTTGTTAGGACCCCAAT	qRT-PCR	*(115)*
MR1194_LjSbtM1_R	TTGAGCAGCACCCTCTCTATC	qRT-PCR	*(115)*
MR1297_HZ146_LjCP3_F	CTATTGACGCAAGTGGCTATG	qRT-PCR	
MR1298_HZ146_LjCP3_R	TTGGTTCCATCATCACTAGCAC	qRT-PCR	
VG721_LjVIH2_ attB1	AAAAAGCAGGCTTCATGACGGCGACGGTGGAG	Cloning	This study
VG722_attB2_ LjVIH2^{KD}	AGAAAGCTGGGTCTTAAATTGCTGAAGAAAGA TGAGGTG	Cloning	This study
VG723_attB2_ LjVIH2^{KD}_V5	CTTACCTCCTCCAGATCCAATTGCTGAAGAAAG ATGAGGTG	Cloning	This study

Table S3. Nutrient composition of field-collected topsoil with moderate nitrogen, phosphorus and potassium availability.

[%]			[mg] per kg of soil					
C	N	S	P	K	B	Mn	Cu	pH
2.36	0.21	0.026	36.2	93.6	0.39	160.13	10.66	5.44

Table S4. Species and databases used for phylogenetic analysis.

Algae

	Species	Version	Database	Database Link	Reference
1	*Rhodosorus marinus*	n/a	Phycocosm *(116)*	https://phycocosm.jgi.doe.gov/phycocosm/home	*(117)*
2	*Galdieria phlegrea*	n/a	Phycocosm *(116)*	https://phycocosm.jgi.doe.gov/phycocosm/home	*(118)*
3	*Porphyridium purpureum*	n/a	Phycocosm *(116)*	https://phycocosm.jgi.doe.gov/phycocosm/home	*(119)*
4	*Cyanophora paradoxa*	n/a	Phycocosm *(116)*	https://phycocosm.jgi.doe.gov/phycocosm/home	*(120)*
5	*Chara braunii*	Cbr_1.0	Ensembl Plants release 61	https://plants.ensembl.org/index.html	*(121)*
6	*Chlamydomonas reinhardtii*	v5.6	Phytozome v13 *(122)*	https://phytozome.jgi.doe.gov/	*(123)*
7	*Volvox carteri*	v2.1	Phytozome v13 *(122)*	https://phytozome.jgi.doe.gov/	*(124)*

Land plants

	Species	Version	Database	Database Link	Reference
1	*Marchantia paleacea*	ASM1418076v2	NCBI	https://www.ncbi.nlm.nih.gov/datasets/genome/GCA_014180765.2/	*(125)*
2	*Marchantia polymorpha*	v3.1	Phytozome v13 *(122)*	https://phytozome.jgi.doe.gov/	*(126)*

3	*Physcomitrium patens*	v3.3	Phytozome v13 *(122)*	https://phytozome.jgi.doe.gov/	*(127)*
4	*Selaginella moellendorffii*	v1.0	Phytozome v13 *(122)*	https://phytozome.jgi.doe.gov/	*(128)*
5	*Ginkgo biloba*	Version-2021	Ginkgo DB	https://ginkgo.zju.edu.cn/genome/ftp/	*(129)*
6	*Amborella trichopoda*	v1.0	Phytozome v13 *(122)*	https://phytozome.jgi.doe.gov/	*(130)*
7	*Aristolochia fimbriata*	ASM1984555v1	ENA/EMBL	https://www.ebi.ac.uk/ena/browser/view/GCA_019845555.1	*(131)*
8	*Phalaenopsis equestris*	ASM126359v1	NCBI	https://www.ncbi.nlm.nih.gov/datasets/genome/GCF_001263595.1/	*(132)*
9	*Musa acuminata*	v2	Ensembl Plants release 61	https://plants.ensembl.org/index.html	*(133)*
10	*Zea mays*	Z.mays_LH145 v1.2	Phytozome v13 *(122)*	https://phytozome.jgi.doe.gov/	*(134)*
11	*Oryza sativa*	IRGSP-1.0	Ensembl Plants release 61	https://plants.ensembl.org/index.html	*(135)*
12	*Hordeum vulgare*	Morex V3	Phytozome v13 *(122)*	https://phytozome.jgi.doe.gov/	*(136)*
13	*Beta vulgaris*	EL10_1.0	Phytozome v13 *(122)*	https://phytozome.jgi.doe.gov/	*(137)*
14	*Parasponia andersonii*	PanWU01x14_asm01	NCBI	https://www.ncbi.nlm.nih.gov/datasets/genome/GCA_002914805.1/	*(138)*
15	*Casuarina glauca*	ASM325504v1	GigaDB	https://gigadb.org/dataset/101051	*(139)*
16	*Datisca glomerata*	ASM325502v1	GigaDB	http://gigadb.org/dataset/view/id/101046	*(139)*
17	*Lupinus albus*	v1	Phytozome v13 *(122)*	https://phytozome.jgi.doe.gov/	*(140)*
18	*Medicago truncatula*	Mt4.0v1	Phytozome v13 *(122)*	https://phytozome.jgi.doe.gov/	*(141)*
19	*Glycine max*	v2.1	Ensembl Plants release 61	https://plants.ensembl.org/index.html	*(142)*

20	*Lotus japonicus*	Gifu v1.2	Lotus Base	https://lotus.au.dk/	*(143)*
21	*Solanum lycopersicum*	ITAG4.0	SolGenomics Network *(144)*	https://solgenomics.sgn.cornell.edu/	*(145)*
22	*Solanum tuberosum*	v6.1	SpudDB	https://spuddb.uga.edu/	*(146)*
23	*Arabidopsis thaliana*	TAIR10	Phytozome v13 *(122)*	https://phytozome.jgi.doe.gov/	*(147)*
24	*Theobroma cacao*	Theobroma_cacao_20110822	Ensembl Plants release 61	https://plants.ensembl.org/index.html	*(148)*
25	*Carica papaya*	Papaya1.0	NCBI	https://www.ncbi.nlm.nih.gov/datasets/genome/GCA_000150535.1/	*(149)*
26	*Citrus clementina*	v1.0	Phytozome v13 *(122)*	https://phytozome.jgi.doe.gov/	*(150)*

REFERENCES

1. J. Paz-Ares, M. I. Puga, M. Rojas-Triana, I. Martinez-Hevia, S. Diaz, C. Poza-Carrión, M. Miñambres, A. Leyva, Plant adaptation to low phosphorus availability: Core signaling, crosstalks, and applied implications. *Mol. Plant* **15**, 104–124 (2022).

2. C. L. Dybas, Dead zones spreading in world oceans. *Bioscience* **55**, 552–557 (2005).

3. V. Rubio, F. Linhares, R. Solano, A. C. Martín, J. Iglesias, A. Leyva, J. Paz-Ares, A conserved MYB transcription factor involved in phosphate starvation signaling both in vascular plants and in unicellular algae. *Genes Dev.* **15**, 2122–2133 (2001).

4. R. Bustos, G. Castrillo, F. Linhares, M. I. Puga, V. Rubio, J. Pérez-Pérez, R. Solano, A. Leyva, J. Paz-Ares, A central regulatory system largely controls transcriptional activation and repression responses to phosphate starvation in *Arabidopsis*. *PLOS Genet.* **6**, e1001102 (2010).

5. L. Sun, L. Song, Y. Zhang, Z. Zheng, D. Liu, Arabidopsis PHL2 and PHR1 act redundantly as the key components of the central regulatory System controlling transcriptional responses to phosphate starvation. *Plant Physiol.* **170**, 499–514 (2016).

6. Z. Wang, Z. Zheng, L. Song, D. Liu, Functional characterization of Arabidopsis PHL4 in plant response to phosphate starvation. *Front. Plant Sci.* **9**, 1432 (2018).

7. F. Ren, Q.-Q. Guo, L.-L. Chang, L. Chen, C.-Z. Zhao, H. Zhong, X.-B. Li, Brassica napus PHR1 gene encoding a MYB-like protein functions in response to phosphate starvation. *PLOS ONE* **7**, e44005 (2012).

8. J. Zhou, F. Jiao, Z. Wu, Y. Li, X. Wang, X. He, W. Zhong, P. Wu, OsPHR2 is involved in phosphate-starvation signaling and excessive phosphate accumulation in shoots of plants. *Plant Physiol.* **146**, 1673–1686 (2008).

9. J. Wang, J. Sun, J. Miao, J. Guo, Z. Shi, M. He, Y. Chen, X. Zhao, B. Li, F. Han, Y. Tong, Z. Li, A phosphate starvation response regulator Ta-PHR1 is involved in phosphate signalling and increases grain yield in wheat. *Ann. Bot.* **111**, 1139–1153 (2013).

10. P. Wang, R. Snijders, W. Kohlen, J. Liu, T. Bisseling, E. Limpens, Medicago SPX1 and SPX3 regulate phosphate homeostasis, mycorrhizal colonization, and arbuscule degradation. *Plant Cell* **33**, 3470–3486 (2021).

11. D. Das, M. Paries, K. Hobecker, M. Gigl, C. Dawid, H.-M. Lam, J. Zhang, M. Chen, C. Gutjahr, PHOSPHATE STARVATION RESPONSE transcription factors enable arbuscular mycorrhiza symbiosis. *Nat. Commun.* **13**, 477 (2022).

12. Q. Lv, Y. Zhong, Y. Wang, Z. Wang, L. Zhang, J. Shi, Z. Wu, Y. Liu, C. Mao, K. Yi, P. Wu, SPX4 negatively regulates phosphate signaling and homeostasis through its interaction with PHR2 in rice. *Plant Cell* **26**, 1586–1597 (2014).

13. M. I. Puga, I. Mateos, R. Charukesi, Z. Wang, J. M. Franco-Zorrilla, L. de Lorenzo, M. L. Irigoyen, S. Masiero, R. Bustos, J. Rodríguez, A. Leyva, V. Rubio, H. Sommer, J. Paz-Ares, SPX1 is a phosphate-dependent inhibitor of PHOSPHATE STARVATION RESPONSE 1 in *Arabidopsis*. *Proc. Natl. Acad. Sci. U.S.A.* **111**, 14947–14952 (2014).

14. Z. Wang, W. Ruan, J. Shi, L. Zhang, D. Xiang, C. Yang, C. Li, Z. Wu, Y. Liu, Y. Yu, H. Shou, X. Mo, C. Mao, P. Wu, Rice SPX1 and SPX2 inhibit phosphate starvation responses through interacting with PHR2 in a phosphate-dependent manner. *Proc. Natl. Acad. Sci. U.S.A.* **111**, 14953–14958 (2014).

15. W. Qi, I. W. Manfield, S. P. Muench, A. Baker, AtSPX1 affects the AtPHR1–DNA-binding equilibrium by binding monomeric AtPHR1 in solution. *Biochem. J.* **474**, 3675–3687 (2017).

16. Y. Zhong, Y. Wang, J. Guo, X. Zhu, J. Shi, Q. He, Y. Liu, Y. Wu, L. Zhang, Q. Lv, C. Mao, Rice SPX6 negatively regulates the phosphate starvation response through suppression of the transcription factor PHR2. *New Phytol.* **219**, 135–148 (2018).

17. M. B. Osorio, S. Ng, O. Berkowitz, I. De Clercq, C. Mao, H. Shou, J. Whelan, R. Jost, SPX4 Acts on PHR1-dependent and -independent regulation of shoot phosphorus status in Arabidopsis. *Plant Physiol.* **181**, 332–352 (2019).

18. R. Wild, R. Gerasimaite, J.-Y. Jung, V. Truffault, I. Pavlovic, A. Schmidt, A. Saiardi, H. J. Jessen, Y. Poirier, M. Hothorn, A. Mayer, Control of eukaryotic phosphate homeostasis by inositol polyphosphate sensor domains. *Science* **352**, 986–990 (2016).

19. M. K. Ried, R. Wild, J. Zhu, J. Pipercevic, K. Sturm, L. Broger, R. K. Harmel, L. A. Abriata, L. A. Hothorn, D. Fiedler, S. Hiller, M. Hothorn, Inositol pyrophosphates promote the interaction of SPX domains with the coiled-coil motif of PHR transcription factors to regulate plant phosphate homeostasis. *Nat. Commun.* **12**, 384 (2021).

20. J. Dong, G. Ma, L. Sui, M. Wei, V. Satheesh, R. Zhang, S. Ge, J. Li, T. E. Zhang, C. Wittwer, H. J. Jessen, H. Zhang, G. Y. An, D. Y. Chao, D. Liu, M. Lei, Inositol pyrophosphate InsP8 acts as an intracellular phosphate signal in *Arabidopsis*. *Mol. Plant* **12**, 1463–1473 (2019).

21. V. Raboy, *Low phytic acid* crops: Observations based on four decades of research. *Plants* **9**, 140 (2020).

22. S. B. Shears, Inositol pyrophosphates: Why so many phosphates? *Adv. Biol. Regul.* **57**, 203–216 (2015).

23. J. Zhu, K. Lau, R. Puschmann, R. K. Harmel, Y. Zhang, V. Pries, P. Gaugler, L. Broger, A. K. Dutta, H. J. Jessen, G. Schaaf, A. R. Fernie, L. A. Hothorn, D. Fiedler, M. Hothorn, Two bifunctional inositol pyrophosphate kinases/phosphatases control plant phosphate homeostasis. *eLife* **8**, e43582 (2019).

24. E. Riemer, D. Qiu, D. Laha, R. K. Harmel, P. Gaugler, V. Gaugler, M. Frei, M.-R. Hajirezaei, N. P. Laha, L. Krusenbaum, R. Schneider, A. Saiardi, D. Fiedler, H. J. Jessen, G. Schaaf, R. F. H. Giehl, ITPK1 is an InsP6/ADP phosphotransferase that controls phosphate signaling in *Arabidopsis*. *Mol. Plant* **14**, 1864–1880 (2021).

25. C. Cridland, A. Russo, B. Craige, J. Donahue, X. M. Zhang, M. Payne, G. Gillaspy, C. Freed, Enhancing inositol pyrophosphate accumulation in plants alters growth, phosphate homeostasis, and insect herbivory. *Plant J.* **123**, e70315 (2025).

26. D. Laha, P. Johnen, C. Azevedo, M. Dynowski, M. Weiß, S. Capolicchio, H. Mao, T. Iven, M. Steenbergen, M. Freyer, P. Gaugler, M. K. F. de Campos, N. Zheng, I. Feussner, H. J. Jessen, S. C. M. Van Wees, A. Saiardi, G. Schaaf, VIH2 regulates the synthesis of inositol pyrophosphate $InsP_8$ and jasmonate-dependent defenses in *Arabidopsis*. *Plant Cell* **27**, 1082–1097 (2015).

27. M. Desai, P. Rangarajan, J. L. Donahue, S. P. Williams, E. S. Land, M. K. Mandal, B. Q. Phillippy, I. Y. Perera, V. Raboy, G. E. Gillaspy, Two inositol hexakisphosphate kinases drive inositol pyrophosphate synthesis in plants. *Plant J.* **80**, 642–653 (2014).

28. H. Wang, V. S. Nair, A. A. Holland, S. Capolicchio, H. J. Jessen, M. K. Johnson, S. B. Shears, Asp1 from Schizosaccharomyces pombe binds a [2Fe-2S](2+) cluster which inhibits inositol pyrophosphate 1-phosphatase activity. *Biochemistry* **54**, 6462–6474 (2015).

29. M. Pascual-Ortiz, A. Saiardi, E. Walla, V. Jakopec, N. A. Künzel, I. Span, A. Vangala, U. Fleig, Asp1 bifunctional activity modulates spindle function via controlling cellular inositol pyrophosphate levels in *Schizosaccharomyces pombe*. *Mol. Cell. Biol.* **38**, e00047-18 (2018).

30. D. E. Dollins, W. Bai, P. C. Fridy, J. C. Otto, J. L. Neubauer, S. G. Gattis, K. P. M. Mehta, J. D. York, Vip1 is a kinase and pyrophosphatase switch that regulates inositol diphosphate signaling. *Proc. Natl. Acad. Sci. U.S.A.* **117**, 9356–9364 (2020).

31. E. Riemer, N. J. Pullagurla, R. Yadav, P. Rana, H. J. Jessen, M. Kamleitner, G. Schaaf, D. Laha, Regulation of plant biotic interactions and abiotic stress responses by inositol polyphosphates. *Front. Plant Sci.* **13**, 944515 (2022).

32. H. Whitfield, G. White, C. Sprigg, A. M. Riley, B. V. L. Potter, A. M. Hemmings, C. A. Brearley, An ATP-responsive metabolic cassette comprised of inositol tris/tetrakisphosphate kinase 1 (ITPK1) and inositol pentakisphosphate 2-kinase (IPK1) buffers diphosphosphoinositol phosphate levels. *Biochem. J.* **477**, 2621–2638 (2020).

33. J. Shi, X. Wang, E. Wang, Mycorrhizal symbiosis in plant growth and stress adaptation: From genes to ecosystems. *Annu. Rev. Plant Biol.* **74**, 569–607 (2023).

34. M. C. Brundrett, L. Tedersoo, Evolutionary history of mycorrhizal symbioses and global host plant diversity. *New Phytol.* **220**, 1108–1115 (2018).

35. W. Remy, T. N. Taylor, H. Hass, H. Kerp, Four hundred-million-year-old vesicular arbuscular mycorrhizae. *Proc. Natl. Acad. Sci. U.S.A.* **91**, 11841–11843 (1994).

36. B. J. W. Mills, S. A. Batterman, K. J. Field, Nutrient acquisition by symbiotic fungi governs Palaeozoic climate transition. *Philos. Trans. R. Soc. Lond. B Biol. Sci.* **373**, 20160503 (2018).

37. A. Genre, M. Chabaud, T. Timmers, P. Bonfante, D. G. Barker, Arbuscular mycorrhizal fungi elicit a novel intracellular apparatus in *Medicago truncatula* root epidermal cells before infection. *Plant Cell* **17**, 3489–3499 (2005).

38. A. Genre, M. Chabaud, A. Faccio, D. G. Barker, P. Bonfante, Prepenetration apparatus assembly precedes and predicts the colonization patterns of arbuscular mycorrhizal fungi within the root cortex of both *Medicago truncatula* and *Daucus carota*. *Plant Cell* **20**, 1407–1420 (2008).

39. M. J. Harrison, Cellular programs for arbuscular mycorrhizal symbiosis. *Curr. Opin. Plant Biol.* **15**, 691–698 (2012).

40. S. Campo, B. San Segundo, Systemic induction of phosphatidylinositol-based signaling in leaves of arbuscular mycorrhizal rice plants. *Sci. Rep.* **10**, 15896 (2020).

41. D. Liao, C. Sun, H. Liang, Y. Wang, X. Bian, C. Dong, X. Niu, M. Yang, G. Xu, A. Chen, S. Wu, SlSPX1-SlPHR complexes mediate the suppression of arbuscular mycorrhizal symbiosis by phosphate repletion in tomato. *Plant Cell* **34**, 4045–4065 (2022).

42. J. Shi, B. Zhao, S. Zheng, X. Zhang, X. Wang, W. Dong, Q. Xie, G. Wang, Y. Xiao, F. Chen, N. Yu, E. Wang, A phosphate starvation response-centered network regulates mycorrhizal symbiosis. *Cell* **184**, 5527–5540.e18 (2021).

43. P. Wang, Y. Zhong, Y. Li, W. Zhu, Y. Zhang, J. Li, Z. Chen, E. Limpens, The phosphate starvation response regulator PHR2 antagonizes arbuscule maintenance in Medicago. *New Phytol.* **244**, 1979–1993 (2024).

44. F. Lota, S. Wegmüller, B. Buer, S. Sato, A. Bräutigam, B. Hanf, M. Bucher, The *cis*-acting CTTC-P1BS module is indicative for gene function of *LjVTI12*, a Qb-SNARE protein gene that is required for arbuscule formation in *Lotus japonicus*. *Plant J.* **74**, 280–293 (2013).

45. A. Chen, M. Gu, S. Sun, L. Zhu, S. Hong, G. Xu, Identification of two conserved cis-acting elements, MYCS and P1BS, involved in the regulation of mycorrhiza-activated phosphate transporters in eudicot species. *New Phytol.* **189**, 1157–1169 (2011).

46. L. H. Luginbuehl, G. N. Menard, S. Kurup, H. Van Erp, G. V. Radhakrishnan, A. Breakspear, G. E. D. Oldroyd, P. J. Eastmond, Fatty acids in arbuscular mycorrhizal fungi are synthesized by the host plant. *Science* **356**, 1175–1178 (2017).

47. Y. Jiang, W. Wang, Q. Xie, N. Liu, L. Liu, D. Wang, X. Zhang, C. Yang, X. Chen, D. Tang, E. Wang, Plants transfer lipids to sustain colonization by mutualistic mycorrhizal and parasitic fungi. *Science* **356**, 1172–1175 (2017).

48. A. Keymer, P. Pimprikar, V. Wewer, C. Huber, M. Brands, S. L. Bucerius, P.-M. Delaux, V. Klingl, E. von Röpenack-Lahaye, T. L. Wang, W. Eisenreich, P. Dörmann, M. Parniske, C. Gutjahr, Lipid transfer from plants to arbuscular mycorrhiza fungi. *eLife* **6**, e29107 (2017).

49. S. E. Smith, F. A. Smith, Roles of arbuscular mycorrhizas in plant nutrition and growth: New paradigms from cellular to ecosystem scales. *Annu. Rev. Plant Biol.* **62**, 227–250 (2011).

50. N. Marro, G. Grilli, F. Soteras, M. Caccia, S. Longo, N. Cofré, V. Borda, M. Burni, M. Janoušková, C. Urcelay, The effects of arbuscular mycorrhizal fungal species and taxonomic groups on stressed and unstressed plants: A global meta-analysis. *New Phytol.* **235**, 320–332 (2022).

51. F. Breuillin, J. Schramm, M. Hajirezaei, A. Ahkami, P. Favre, U. Druege, B. Hause, M. Bucher, T. Kretzschmar, E. Bossolini, C. Kuhlemeier, E. Martinoia, P. Franken, U. Scholz, D. Reinhardt,

Phosphate systemically inhibits development of arbuscular mycorrhiza in Petunia hybrida and represses genes involved in mycorrhizal functioning. *Plant J.* **64**, 1002–1017 (2010).

52. C. Balzergue, V. Puech-Pagès, G. Bécard, S. F. Rochange, The regulation of arbuscular mycorrhizal symbiosis by phosphate in pea involves early and systemic signalling events. *J. Exp. Bot.* **62**, 1049–1060 (2011).

53. T. Mun, A. Bachmann, V. Gupta, J. Stougaard, S. U. Andersen, *Lotus* Base: An integrated information portal for the model legume *Lotus japonicus*. *Sci. Rep.* **6**, 39447 (2016).

54. S. Osada, K. Kageyama, Y. Ohnishi, J.-I. Nishikawa, T. Nishihara, M. Imagawa, Inositol phosphate kinase Vip1p interacts with histone chaperone Asf1p in *Saccharomyces cerevisiae*. *Mol. Biol. Rep.* **39**, 4989–4996 (2012).

55. S. Mulugu, W. Bai, P. C. Fridy, R. J. Bastidas, J. C. Otto, D. E. Dollins, T. A. Haystead, A. A. Ribeiro, J. D. York, A conserved family of enzymes that phosphorylate inositol hexakisphosphate. *Science* **316**, 106–109 (2007).

56. S. M. N. Onnebo, A. Saiardi, Inositol pyrophosphates modulate hydrogen peroxide signalling. *Biochem. J.* **423**, 109–118 (2009).

57. H. Lin, P. C. Fridy, A. A. Ribeiro, J. H. Choi, D. K. Barma, G. Vogel, J. R. Falck, S. B. Shears, J. D. York, G. W. Mayr, Structural analysis and detection of biological inositol pyrophosphates reveal that the family of VIP/diphosphoinositol pentakisphosphate kinases are 1/3-kinases. *J. Biol. Chem.* **284**, 1863–1872 (2009).

58. G. Liu, E. Riemer, R. Schneider, D. Cabuzu, O. Bonny, C. A. Wagner, D. Qiu, A. Saiardi, A. Strauss, T. Lahaye, G. Schaaf, T. Knoll, J. P. Jessen, H. J. Jessen, The phytase RipBL1 enables the assignment of a specific inositol phosphate isomer as a structural component of human kidney stones. *RSC Chem. Biol.* **4**, 300–309 (2023).

59. D. Qiu, C. Gu, G. Liu, K. Ritter, V. B. Eisenbeis, T. Bittner, A. Gruzdev, L. Seidel, B. Bengsch, S. B. Shears, H. J. Jessen, Capillary electrophoresis mass spectrometry identifies new isomers of inositol pyrophosphates in mammalian tissues. *Chem. Sci.* **14**, 658–667 (2023).

60. D. Qiu, V. B. Eisenbeis, A. Saiardi, H. J. Jessen, Absolute quantitation of inositol pyrophosphates by capillary electrophoresis electrospray ionization mass spectrometry. *J. Vis. Exp.*, 10.3791/62847-v (2021).

61. D. Qiu, M. S. Wilson, V. B. Eisenbeis, R. K. Harmel, E. Riemer, T. M. Haas, C. Wittwer, N. Jork, C. Gu, S. B. Shears, G. Schaaf, B. Kammerer, D. Fiedler, A. Saiardi, H. J. Jessen, Analysis of inositol phosphate metabolism by capillary electrophoresis electrospray ionization mass spectrometry. *Nat. Commun.* **11**, 6035 (2020).

62. A. Małolepszy, T. Mun, N. Sandal, V. Gupta, M. Dubin, D. Urbański, N. Shah, A. Bachmann, E. Fukai, H. Hirakawa, S. Tabata, M. Nadzieja, K. Markmann, J. Su, Y. Umehara, T. Soyano, A. Miyahara, S. Sato, M. Hayashi, J. Stougaard, S. U. Andersen, The LORE1 insertion mutant resource. *Plant J.* **88**, 306–317 (2016).

63. P. Gaugler, V. Gaugler, M. Kamleitner, G. Schaaf, Extraction and quantification of soluble, radiolabeled inositol polyphosphates from different plant species using SAX-HPLC. *J. Vis. Exp.*, 10.3791/61495 (2020).

64. D. S. Floss, S. K. Gomez, H.-J. Park, A. M. MacLean, L. M. Müller, K. K. Bhattarai, V. Lévesque-Tremblay, I. E. Maldonado-Mendoza, M. J. Harrison, A transcriptional program for arbuscule degeneration during AM symbiosis is regulated by MYB1. *Curr. Biol.* **27**, 1206–1212 (2017).

65. L. Arata, E. Fabrizi, P. Sckokai, A worldwide analysis of trend in crop yields and yield variability: Evidence from FAO data. *Econ. Model.* **90**, 190–208 (2020).

66. R. J. H. Sawers, C. Gutjahr, U. Paszkowski, Cereal mycorrhiza: An ancient symbiosis in modern agriculture. *Trends Plant Sci.* **13**, 93–97 (2008).

67. E. Verbruggen, M. G. A. van der Heijden, M. C. Rillig, E. T. Kiers, Mycorrhizal fungal establishment in agricultural soils: Factors determining inoculation success. *New Phytol.* **197**, 1104–1109 (2013).

68. M. Moora, J. Davison, M. Öpik, M. Metsis, Ü. Saks, T. Jairus, M. Vasar, M. Zobel, Anthropogenic land use shapes the composition and phylogenetic structure of soil arbuscular mycorrhizal fungal communities. *FEMS Microbiol. Ecol.* **90**, 609–621 (2014).

69. D. Xiang, E. Verbruggen, Y. Hu, S. D. Veresoglou, M. C. Rillig, W. Zhou, T. Xu, H. Li, Z. Hao, Y. Chen, B. Chen, Land use influences arbuscular mycorrhizal fungal communities in the farming-pastoral ecotone of northern China. *New Phytol.* **204**, 968–978 (2014).

70. M. Giovannetti, C. Göschl, C. Dietzen, S. U. Andersen, S. Kopriva, W. Busch, Identification of novel genes involved in phosphate accumulation in *Lotus japonicus* through Genome Wide Association mapping of root system architecture and anion content. *PLOS Genet.* **15**, e1008126 (2019).

71. K. Aung, S.-I. Lin, C.-C. Wu, Y.-T. Huang, C.-L. Su, T.-J. Chiou, pho2, a phosphate overaccumulator, is caused by a nonsense mutation in a microRNA399 target gene. *Plant Physiol.* **141**, 1000–1011 (2006).

72. R. Bari, B. Datt Pant, M. Stitt, W.-R. Scheible, PHO2, microRNA399, and PHR1 define a phosphate-signaling pathway in plants. *Plant Physiol.* **141**, 988–999 (2006).

73. S.-I. Lin, S.-F. Chiang, W.-Y. Lin, J.-W. Chen, C.-Y. Tseng, P.-C. Wu, T.-J. Chiou, Regulatory network of microRNA399 and PHO2 by systemic signaling. *Plant Physiol.* **147**, 732–746 (2008).

74. R. Huertas, I. Torres-Jerez, S. J. Curtin, W. Scheible, M. Udvardi, *Medicago truncatula PHO2* genes have distinct roles in phosphorus homeostasis and symbiotic nitrogen fixation. *Front. Plant Sci.* **14**, 1211107 (2023).

75. H. Vierheilig, J. M. Garcia-Garrido, U. Wyss, Y. Piché, Systemic suppression of mycorrhizal colonization of barley roots already colonized by AM fungi. *Soil Biol. Biochem.* **32**, 589–595 (2000).

76. N. Imin, N. A. Mohd-Radzman, H. A. Ogilvie, M. A. Djordjevic, The peptide-encoding CEP1 gene modulates lateral root and nodule numbers in *Medicago truncatula*. *J. Exp. Bot.* **64**, 5395–5409 (2013).

77. L. Pedinotti, J. Teyssendier de la Serve, T. Roudaire, H. San Clemente, M. Aguilar, W. Kohlen, F. Frugier, N. Frei Dit Frey, The CEP peptide-CRA2 receptor module promotes arbuscular mycorrhizal symbiosis. *Curr. Biol.* **34**, 5366–5373.e4 (2024).

78. N. A. Mohd-Radzman, C. Laffont, A. Ivanovici, N. Patel, D. Reid, J. Stougaard, F. Frugier, N. Imin, M. A. Djordjevic, Different pathways act downstream of the CEP peptide receptor CRA2 to regulate lateral root and nodule development. *Plant Physiol.* **171**, 2536–2548 (2016).

79. M. G. Mitchum, X. Wang, E. L. Davis, Diverse and conserved roles of CLE peptides. *Curr. Opin. Plant Biol.* **11**, 75–81 (2008).

80. R. V. Penmetsa, J. A. Frugoli, L. S. Smith, S. R. Long, D. R. Cook, Dual genetic pathways controlling nodule number in *Medicago truncatula*. *Plant Physiol.* **131**, 998–1008 (2003).

81. E. Schnabel, E.-P. Journet, F. de Carvalho-Niebel, G. Duc, J. Frugoli, The *Medicago truncatula* SUNN gene encodes a CLV1-like leucine-rich repeat receptor kinase that regulates nodule number and root length. *Plant Mol. Biol.* **58**, 809–822 (2005).

82. M. Karlo, C. Boschiero, K. G. Landerslev, G. S. Blanco, J. Wen, K. S. Mysore, X. Dai, P. X. Zhao, T. C. de Bang, The CLE53-SUNN genetic pathway negatively regulates arbuscular mycorrhiza root colonization in *Medicago truncatula*. *J. Exp. Bot.* **71**, 4972–4984 (2020).

83. L. M. Müller, K. Flokova, E. Schnabel, X. Sun, Z. Fei, J. Frugoli, H. J. Bouwmeester, M. J. Harrison, A CLE-SUNN module regulates strigolactone content and fungal colonization in arbuscular mycorrhiza. *Nat. Plants* **5**, 933–939 (2019).

84. R. Müller, A. Bleckmann, R. Simon, The receptor kinase CORYNE of *Arabidopsis* transmits the stem cell-limiting signal CLAVATA3 independently of CLAVATA1. *Plant Cell* **20**, 934–946 (2008).

85. S. Bashyal, H. Everett, S. Matsuura, L. M. Müller, A plant CLE peptide and its fungal mimic promote arbuscular mycorrhizal symbiosis via CRN-mediated ROS suppression. *Proc. Natl. Acad. Sci. U.S.A.* **122**, e2422215122 (2025).

86. M. Le Marquer, G. Bécard, N. Frei Dit Frey, Arbuscular mycorrhizal fungi possess a CLAVATA3/embryo surrounding region-related gene that positively regulates symbiosis. *New Phytol.* **222**, 1030–1042 (2019).

87. S. M. Schmidt, M. Belisle, W. B. Frommer, The evolving landscape around genome editing in agriculture: Many countries have exempted or move to exempt forms of genome editing from GMO regulation of crop plants. *EMBO Rep.* **21**, e50680 (2020).

88. N. P. Laha, R. F. H. Giehl, E. Riemer, D. Qiu, N. J. Pullagurla, R. Schneider, Y. W. Dhir, R. Yadav, Y. E. Mihiret, P. Gaugler, V. Gaugler, H. Mao, N. Zheng, N. von Wirén, A. Saiardi, S. Bhattacharjee, H. J. Jessen, D. Laha, G. Schaaf, INOSITOL (1,3,4) TRIPHOSPHATE 5/6 KINASE1-dependent inositol polyphosphates regulate auxin responses in Arabidopsis. *Plant Physiol.* **190**, 2722–2738 (2022).

89. P. Gaugler, R. Schneider, G. Liu, D. Qiu, J. Weber, J. Schmid, N. Jork, M. Häner, K. Ritter, N. Fernández-Rebollo, R. F. H. Giehl, M. N. Trung, R. Yadav, D. Fiedler, V. Gaugler, H. J. Jessen, G. Schaaf, D. Laha, *Arabidopsis* PFA-DSP-type phosphohydrolases target specific inositol pyrophosphate messengers. *Biochemistry* **61**, 1213–1227 (2022).

90. F. Laurent, S. M. Bartsch, A. Shukla, F. Rico-Resendiz, D. Couto, C. Fuchs, J. Nicolet, S. Loubéry, H. J. Jessen, D. Fiedler, M. Hothorn, Inositol pyrophosphate catabolism by three families of phosphatases regulates plant growth and development. *PLOS Genet.* **20**, e1011468 (2024).

91. K. Chalak, R. Yadav, G. Liu, P. Rana, H. J. Jessen, D. Laha, Functional conservation of the DDP1-type inositol pyrophosphate phosphohydrolases in land plant. *Biochemistry* **63**, 2723–2728 (2024).

92. C. Freed, B. Craige, J. Donahue, C. Cridland, S. P. Williams, C. Pereira, J. Kim, H. Blice, J. Owen Jr, G. Gillaspy, Using native and synthetic genes to disrupt inositol pyrophosphates and phosphate accumulation in plants. *Plant Physiol.* **197**, kiae582 (2024).

93. R. Schneider, K. Lami, I. Prucker, S. C. Stolze, A. Strauß, J. M. Schmidt, S. M. Bartsch, K. Langenbach, E. Lange, K. Ritter, D. Furkert, N. Faiß, S. Kumar, M. Shamim Hasan, A. Makris, L. Krusenbaum, S. Wege, Y. Z. Belay, S. Kriescher, J. The, M. Harings, F. M. W. Grundler, M. K. Ried-Lasi, H. Schoof, P. Gaugler, M. Kamleitner, D. Fiedler, H. Nakagami, R. F. H. Giehl, T. Lahaye, S. Bhattacharjee, H. J. Jessen, V. Gaugler, G. Schaaf, NUDIX hydrolases target specific inositol pyrophosphates and regulate phosphate homeostasis and bacterial pathogen susceptibility in *Arabidopsis*. *J. Integr. Plant Biol.* **67**, 3123–3151 (2025).

94. S. E. Smith, D. J. Read, *Mycorrhizal Symbiosis* (Academic Press, ed. 3, 2010).

95. M. Bigalke, A. Ulrich, A. Rehmus, A. Keller, Accumulation of cadmium and uranium in arable soils in Switzerland. *Environ. Pollut.* **221**, 85–93 (2017).

96. M. Abdalla, M. Bitterlich, J. Jansa, D. Püschel, M. A. Ahmed, The role of arbuscular mycorrhizal symbiosis in improving plant water status under drought. *J. Exp. Bot.* **74**, 4808–4824 (2023).

97. H.-J. Hawkins, R. I. M. Cargill, M. E. Van Nuland, S. C. Hagen, K. J. Field, M. Sheldrake, N. A. Soudzilovskaia, E. T. Kiers, Mycorrhizal mycelium as a global carbon pool. *Curr. Biol.* **33**, R560–R573 (2023).

98. S. Alberti, A. D. Gitler, S. Lindquist, A suite of Gateway cloning vectors for high-throughput genetic analysis in *Saccharomyces cerevisiae*. *Yeast* **24**, 913–919 (2007).

99. H. Vierheilig, A. P. Coughlan, U. Wyss, Y. Piché, Ink and vinegar, a simple staining technique for arbuscular-mycorrhizal fungi. *Appl. Environ. Microbiol.* **64**, 5004–5007 (1998).

100. T. P. McGonigle, M. H. Miller, D. G. Evans, G. L. Fairchild, J. A. Swan, A new method which gives an objective measure of colonization of roots by vesicular-arbuscular mycorrhizal fungi. *New Phytol.* **115**, 495–501 (1990).

101. D. Dreher, H. Yadav, S. Zander, B. Hause, Is there genetic variation in mycorrhization of *Medicago truncatula*? *PeerJ* **5**, e3713 (2017).

102. M. Sexauer, H. Bhasin, M. Schön, E. Roitsch, C. Wall, U. Herzog, K. Markmann, A micro RNA mediates shoot control of root branching. *Nat. Commun.* **14**, 8083 (2023).

103. R. D. Gietz, R. H. Schiestl, A. R. Willems, R. A. Woods, Studies on the transformation of intact yeast cells by the LiAc/SS-DNA/PEG procedure. *Yeast* **11**, 355–360 (1995).

104. B. J. M. Zonneveld, Cheap and simple yeast media. *J. Microbiol. Methods* **4**, 287–291 (1986).

105. C. Azevedo, A. Saiardi, Extraction and analysis of soluble inositol polyphosphates from yeast. *Nat. Protoc.* **1**, 2416–2422 (2006).

106. O. Losito, Z. Szijgyarto, A. C. Resnick, A. Saiardi, Inositol pyrophosphates and their unique metabolic complexity: Analysis by gel electrophoresis. *PLOS ONE* **4**, e5580 (2009).

107. D. Laha, N. Parvin, A. Hofer, R. F. H. Giehl, N. Fernandez-Rebollo, N. von Wirén, A. Saiardi, H. J. Jessen, G. Schaaf, *Arabidopsis* ITPK1 and ITPK2 have an evolutionarily conserved phytic acid kinase activity. *ACS Chem. Biol.* **14**, 2127–2133 (2019).

108. R. K. Harmel, R. Puschmann, M. Nguyen Trung, A. Saiardi, P. Schmieder, D. Fiedler, Harnessing 13C-labeled myo-inositol to interrogate inositol phosphate messengers by NMR. *Chem. Sci.* **10**, 5267–5274 (2019).

109. R. Schneider, K. Lami, I. Prucker, S. C. Stolze, A. Strauß, K. Langenbach, M. Kamleitner, Y. Z. Belay, K. Ritter, D. Furkert, P. Gaugler, E. Lange, N. Faiß, J. M. Schmidt, M. Harings, L. Krusenbaum, S. Wege, S. Kriescher, The Jeremy, H. Schoof, D. Fiedler, H. Nakagami, R. F. H. Giehl, T. Lahaye, H. J. Jessen, V. Gaugler, G. Schaaf, NUDIX hydrolases target specific inositol pyrophosphates and regulate phosphate and iron homeostasis, and the expression of defense genes in Arabidopsis. bioRxiv 619122 [Preprint] (2024). https://doi.org/10.1101/2024.10.18.619122.

110. M. Blum, H.-Y. Chang, S. Chuguransky, T. Grego, S. Kandasaamy, A. Mitchell, G. Nuka, T. Paysan-Lafosse, M. Qureshi, S. Raj, L. Richardson, G. A. Salazar, L. Williams, P. Bork, A. Bridge, J. Gough, D. H. Haft, I. Letunic, A. Marchler-Bauer, H. Mi, D. A. Natale, M. Necci, C. A. Orengo, A. P. Pandurangan, C. Rivoire, C. J. A. Sigrist, I. Sillitoe, N. Thanki, P. D. Thomas,

S. C. E. Tosatto, C. H. Wu, A. Bateman, R. D. Finn, The InterPro protein families and domains database: 20 Years on. *Nucleic Acids Res.* **49**, D344–D354 (2021).

111. R. C. Edgar, MUSCLE: Multiple sequence alignment with high accuracy and high throughput. *Nucleic Acids Res.* **32**, 1792–1797 (2004).

112. L.-T. Nguyen, H. A. Schmidt, A. von Haeseler, B. Q. Minh, IQ-TREE: A fast and effective stochastic algorithm for estimating maximum-likelihood phylogenies. *Mol. Biol. Evol.* **32**, 268–274 (2015).

113. I. Letunic, P. Bork, Interactive Tree of Life (iTOL) v6: Recent updates to the phylogenetic tree display and annotation tool. *Nucleic Acids Res.* **52**, W78–W82 (2024).

114. V. Volpe, M. Giovannetti, X.-G. Sun, V. Fiorilli, P. Bonfante, The phosphate transporters LjPT4 and MtPT4 mediate early root responses to phosphate status in non mycorrhizal roots. *Plant Cell Environ.* **39**, 660–671 (2016).

115. M. Groth, S. Kosuta, C. Gutjahr, K. Haage, S. L. Hardel, M. Schaub, A. Brachmann, S. Sato, S. Tabata, K. Findlay, T. L. Wang, M. Parniske, Two *Lotus japonicus* symbiosis mutants impaired at distinct steps of arbuscule development. *Plant J.* **75**, 117–129 (2013).

116. I. V. Grigoriev, R. D. Hayes, S. Calhoun, B. Kamel, A. Wang, S. Ahrendt, S. Dusheyko, R. Nikitin, S. J. Mondo, A. Salamov, I. Shabalov, A. Kuo, PhycoCosm, a comparative algal genomics resource. *Nucleic Acids Res.* **49**, D1004–D1011 (2021).

117. Y. Lee, C. H. Cho, C. Noh, J. H. Yang, S. I. Park, Y. M. Lee, J. A. West, D. Bhattacharya, K. Jo, H. S. Yoon, Origin of minicircular mitochondrial genomes in red algae. *Nat. Commun.* **14**, 3363 (2023).

118. A. W. Rossoni, D. C. Price, M. Seger, D. Lyska, P. Lammers, D. Bhattacharya, A. P. M. Weber, The genomes of polyextremophilic cyanidiales contain 1% horizontally transferred genes with diverse adaptive functions. *eLife* **8**, e45017 (2019).

119. J. Lee, D. Kim, D. Bhattacharya, H. S. Yoon, Expansion of phycobilisome linker gene families in mesophilic red algae. *Nat. Commun.* **10**, 4823 (2019).

120. D. C. Price, U. W. Goodenough, R. Roth, J.-H. Lee, T. Kariyawasam, M. Mutwil, C. Ferrari, F. Facchinelli, S. G. Ball, U. Cenci, C. X. Chan, N. E. Wagner, H. S. Yoon, A. P. M. Weber, D. Bhattacharya, Analysis of an improved *Cyanophora paradoxa* genome assembly. *DNA Res.* **26**, 287–299 (2019).

121. T. Nishiyama, H. Sakayama, J. de Vries, H. Buschmann, D. Saint-Marcoux, K. K. Ullrich, F. B. Haas, L. Vanderstraeten, D. Becker, D. Lang, S. Vosolsobě, S. Rombauts, P. K. I. Wilhelmsson, P. Janitza, R. Kern, A. Heyl, F. Rümpler, L. I. A. C. Villalobos, J. M. Clay, R. Skokan, A. Toyoda, Y. Suzuki, H. Kagoshima, E. Schijlen, N. Tajeshwar, B. Catarino, A. J. Hetherington, A. Saltykova, C. Bonnot, H. Breuninger, A. Symeonidi, G. V. Radhakrishnan, F. Van Nieuwerburgh, D. Deforce, C. Chang, K. G. Karol, R. Hedrich, P. Ulvskov, G. Glöckner, C. F. Delwiche, J. Petrášek, Y. Van de Peer, J. Friml, M. Beilby, L. Dolan, Y. Kohara, S. Sugano, A. Fujiyama, P.-M. Delaux, M. Quint, G. Theißen, M. Hagemann, J. Harholt, C. Dunand, S. Zachgo, J. Langdale, F. Maumus, D. Van Der Straeten, S. B. Gould, S. A. Rensing, The Chara genome: Secondary complexity and implications for plant terrestrialization. *Cell* **174**, 448–464.e24 (2018).

122. D. M. Goodstein, S. Shu, R. Howson, R. Neupane, R. D. Hayes, J. Fazo, T. Mitros, W. Dirks, U. Hellsten, N. Putnam, D. S. Rokhsar, Phytozome: A comparative platform for green plant genomics. *Nucleic Acids Res.* **40**, D1178–86 (2012).

123. S. S. Merchant, S. E. Prochnik, O. Vallon, E. H. Harris, S. J. Karpowicz, G. B. Witman, A. Terry, A. Salamov, L. K. Fritz-Laylin, L. Maréchal-Drouard, W. F. Marshall, L.-H. Qu, D. R. Nelson, A. A. Sanderfoot, M. H. Spalding, V. V. Kapitonov, Q. Ren, P. Ferris, E. Lindquist, H. Shapiro, S. M. Lucas, J. Grimwood, J. Schmutz, P. Cardol, H. Cerutti, G. Chanfreau, C.-L. Chen, V. Cognat, M. T. Croft, R. Dent, S. Dutcher, E. Fernández, H. Fukuzawa, D. González-Ballester, D. González-Halphen, A. Hallmann, M. Hanikenne, M. Hippler, W. Inwood, K. Jabbari, M. Kalanon, R. Kuras, P. A. Lefebvre, S. D. Lemaire, A. V. Lobanov, M. Lohr, A. Manuell, I. Meier, L. Mets, M. Mittag, T. Mittelmeier, J. V. Moroney, J. Moseley, C. Napoli, A. M.

Nedelcu, K. Niyogi, S. V. Novoselov, I. T. Paulsen, G. Pazour, S. Purton, J.-P. Ral, D. M. Riaño-Pachón, W. Riekhof, L. Rymarquis, M. Schroda, D. Stern, J. Umen, R. Willows, N. Wilson, S. L. Zimmer, J. Allmer, J. Balk, K. Bisova, C.-J. Chen, M. Elias, K. Gendler, C. Hauser, M. R. Lamb, H. Ledford, J. C. Long, J. Minagawa, M. D. Page, J. Pan, W. Pootakham, S. Roje, A. Rose, E. Stahlberg, A. M. Terauchi, P. Yang, S. Ball, C. Bowler, C. L. Dieckmann, V. N. Gladyshev, P. Green, R. Jorgensen, S. Mayfield, B. Mueller-Roeber, S. Rajamani, R. T. Sayre, P. Brokstein, I. Dubchak, D. Goodstein, L. Hornick, Y. W. Huang, J. Jhaveri, Y. Luo, D. Martínez, W. C. A. Ngau, B. Otillar, A. Poliakov, A. Porter, L. Szajkowski, G. Werner, K. Zhou, I. V. Grigoriev, D. S. Rokhsar, A. R. Grossman, The *Chlamydomonas* genome reveals the evolution of key animal and plant functions. *Science* **318**, 245–250 (2007).

124. S. E. Prochnik, J. Umen, A. M. Nedelcu, A. Hallmann, S. M. Miller, I. Nishii, P. Ferris, A. Kuo, T. Mitros, L. K. Fritz-Laylin, U. Hellsten, J. Chapman, O. Simakov, S. A. Rensing, A. Terry, J. Pangilinan, V. Kapitonov, J. Jurka, A. Salamov, H. Shapiro, J. Schmutz, J. Grimwood, E. Lindquist, S. Lucas, I. V. Grigoriev, R. Schmitt, D. Kirk, D. S. Rokhsar, Genomic analysis of organismal complexity in the multicellular green alga *Volvox carteri*. *Science* **329**, 223–226 (2010).

125. G. V. Radhakrishnan, J. Keller, M. K. Rich, T. Vernié, D. L. Mbadinga Mbadinga, N. Vigneron, L. Cottret, H. S. Clemente, C. Libourel, J. Cheema, A.-M. Linde, D. M. Eklund, S. Cheng, G. K. S. Wong, U. Lagercrantz, F.-W. Li, G. E. D. Oldroyd, P.-M. Delaux, An ancestral signalling pathway is conserved in intracellular symbioses-forming plant lineages. *Nat. Plants* **6**, 280–289 (2020).

126. J. L. Bowman, T. Kohchi, K. T. Yamato, J. Jenkins, S. Shu, K. Ishizaki, S. Yamaoka, R. Nishihama, Y. Nakamura, F. Berger, C. Adam, S. S. Aki, F. Althoff, T. Araki, M. A. Arteaga-Vazquez, S. Balasubrmanian, K. Barry, D. Bauer, C. R. Boehm, L. Briginshaw, J. Caballero-Perez, B. Catarino, F. Chen, S. Chiyoda, M. Chovatia, K. M. Davies, M. Delmans, T. Demura, T. Dierschke, L. Dolan, A. E. Dorantes-Acosta, D. M. Eklund, S. N. Florent, E. Flores-Sandoval, A. Fujiyama, H. Fukuzawa, B. Galik, D. Grimanelli, J. Grimwood, U. Grossniklaus, T. Hamada, J. Haseloff, A. J. Hetherington, A. Higo, Y. Hirakawa, H. N. Hundley, Y. Ikeda, K. Inoue, S.-I. Inoue, S. Ishida, Q. Jia, M. Kakita, T. Kanazawa, Y. Kawai, T. Kawashima, M. Kennedy, K.

Kinose, T. Kinoshita, Y. Kohara, E. Koide, K. Komatsu, S. Kopischke, M. Kubo, J. Kyozuka, U. Lagercrantz, S.-S. Lin, E. Lindquist, A. M. Lipzen, C.-W. Lu, E. De Luna, R. A. Martienssen, N. Minamino, M. Mizutani, M. Mizutani, N. Mochizuki, I. Monte, R. Mosher, H. Nagasaki, H. Nakagami, S. Naramoto, K. Nishitani, M. Ohtani, T. Okamoto, M. Okumura, J. Phillips, B. Pollak, A. Reinders, M. Rövekamp, R. Sano, S. Sawa, M. W. Schmid, M. Shirakawa, R. Solano, A. Spunde, N. Suetsugu, S. Sugano, A. Sugiyama, R. Sun, Y. Suzuki, M. Takenaka, D. Takezawa, H. Tomogane, M. Tsuzuki, T. Ueda, M. Umeda, J. M. Ward, Y. Watanabe, K. Yazaki, R. Yokoyama, Y. Yoshitake, I. Yotsui, S. Zachgo, J. Schmutz, Insights into land plant evolution garnered from the Marchantia polymorpha genome. *Cell* **171**, 287–304.e15 (2017).

127. D. Lang, K. K. Ullrich, F. Murat, J. Fuchs, J. Jenkins, F. B. Haas, M. Piednoel, H. Gundlach, M. Van Bel, R. Meyberg, C. Vives, J. Morata, A. Symeonidi, M. Hiss, W. Muchero, Y. Kamisugi, O. Saleh, G. Blanc, E. L. Decker, N. van Gessel, J. Grimwood, R. D. Hayes, S. W. Graham, L. E. Gunter, S. F. McDaniel, S. N. W. Hoernstein, A. Larsson, F.-W. Li, P.-F. Perroud, J. Phillips, P. Ranjan, D. S. Rokshar, C. J. Rothfels, L. Schneider, S. Shu, D. W. Stevenson, F. Thümmler, M. Tillich, J. C. Villarreal Aguilar, T. Widiez, G. K.-S. Wong, A. Wymore, Y. Zhang, A. D. Zimmer, R. S. Quatrano, K. F. X. Mayer, D. Goodstein, J. M. Casacuberta, K. Vandepoele, R. Reski, A. C. Cuming, G. A. Tuskan, F. Maumus, J. Salse, J. Schmutz, S. A. Rensing, The *Physcomitrella patens* chromosome-scale assembly reveals moss genome structure and evolution. *Plant J.* **93**, 515–533 (2018).

128. J. A. Banks, T. Nishiyama, M. Hasebe, J. L. Bowman, M. Gribskov, C. dePamphilis, V. A. Albert, N. Aono, T. Aoyama, B. A. Ambrose, N. W. Ashton, M. J. Axtell, E. Barker, M. S. Barker, J. L. Bennetzen, N. D. Bonawitz, C. Chapple, C. Cheng, L. G. G. Correa, M. Dacre, J. DeBarry, I. Dreyer, M. Elias, E. M. Engstrom, M. Estelle, L. Feng, C. Finet, S. K. Floyd, W. B. Frommer, T. Fujita, L. Gramzow, M. Gutensohn, J. Harholt, M. Hattori, A. Heyl, T. Hirai, Y. Hiwatashi, M. Ishikawa, M. Iwata, K. G. Karol, B. Koehler, U. Kolukisaoglu, M. Kubo, T. Kurata, S. Lalonde, K. Li, Y. Li, A. Litt, E. Lyons, G. Manning, T. Maruyama, T. P. Michael, K. Mikami, S. Miyazaki, S.-I. Morinaga, T. Murata, B. Mueller-Roeber, D. R. Nelson, M. Obara, Y. Oguri, R. G. Olmstead, N. Onodera, B. L. Petersen, B. Pils, M. Prigge, S. A. Rensing, D. M. Riaño-Pachón, A. W. Roberts, Y. Sato, H. V. Scheller, B. Schulz, C. Schulz, E. V. Shakirov, N. Shibagaki, N. Shinohara, D. E. Shippen, I. Sørensen, R. Sotooka, N. Sugimoto, M. Sugita, N.

Sumikawa, M. Tanurdzic, G. Theissen, P. Ulvskov, S. Wakazuki, J.-K. Weng, W. W. G. T. Willats, D. Wipf, P. G. Wolf, L. Yang, A. D. Zimmer, Q. Zhu, T. Mitros, U. Hellsten, D. Loqué, R. Otillar, A. Salamov, J. Schmutz, H. Shapiro, E. Lindquist, S. Lucas, D. Rokhsar, I. V. Grigoriev, The Selaginella genome identifies genetic changes associated with the evolution of vascular plants. *Science* **332**, 960–963 (2011).

129. K.-J. Gu, C.-F. Lin, J.-J. Wu, Y.-P. Zhao, GinkgoDB: An ecological genome database for the living fossil, *Ginkgo biloba*. *Database* **2022**, baac046 (2022).

130. Amborella Genome Project, The *Amborella* genome and the evolution of flowering plants. *Science* **342**, 1241089 (2013).

131. L. Qin, Y. Hu, J. Wang, X. Wang, R. Zhao, H. Shan, K. Li, P. Xu, H. Wu, X. Yan, L. Liu, X. Yi, S. Wanke, J. E. Bowers, J. H. Leebens-Mack, C. W. dePamphilis, P. S. Soltis, D. E. Soltis, H. Kong, Y. Jiao, Insights into angiosperm evolution, floral development and chemical biosynthesis from the *Aristolochia fimbriata* genome. *Nat. Plants* **7**, 1239–1253 (2021).

132. J. Cai, X. Liu, K. Vanneste, S. Proost, W.-C. Tsai, K.-W. Liu, L.-J. Chen, Y. He, Q. Xu, C. Bian, Z. Zheng, F. Sun, W. Liu, Y.-Y. Hsiao, Z.-J. Pan, C.-C. Hsu, Y.-P. Yang, Y.-C. Hsu, Y.-C. Chuang, A. Dievart, J.-F. Dufayard, X. Xu, J.-Y. Wang, J. Wang, X.-J. Xiao, X.-M. Zhao, R. Du, G.-Q. Zhang, M. Wang, Y.-Y. Su, G.-C. Xie, G.-H. Liu, L.-Q. Li, L.-Q. Huang, Y.-B. Luo, H.-H. Chen, Y. Van de Peer, Z.-J. Liu, The genome sequence of the orchid *Phalaenopsis equestris*. *Nat. Genet.* **47**, 65–72 (2015).

133. A. D'Hont, F. Denoeud, J.-M. Aury, F.-C. Baurens, F. Carreel, O. Garsmeur, B. Noel, S. Bocs, G. Droc, M. Rouard, C. Da Silva, K. Jabbari, C. Cardi, J. Poulain, M. Souquet, K. Labadie, C. Jourda, J. Lengellé, M. Rodier-Goud, A. Alberti, M. Bernard, M. Correa, S. Ayyampalayam, M. R. Mckain, J. Leebens-Mack, D. Burgess, M. Freeling, D. Mbéguié-A-Mbéguié, M. Chabannes, T. Wicker, O. Panaud, J. Barbosa, E. Hribova, P. Heslop-Harrison, R. Habas, R. Rivallan, P. Francois, C. Poiron, A. Kilian, D. Burthia, C. Jenny, F. Bakry, S. Brown, V. Guignon, G. Kema, M. Dita, C. Waalwijk, S. Joseph, A. Dievart, O. Jaillon, J. Leclercq, X. Argout, E. Lyons, A. Almeida, M. Jeridi, J. Dolezel, N. Roux, A.-M. Risterucci, J. Weissenbach, M. Ruiz, J.-C.

Glaszmann, F. Quétier, N. Yahiaoui, P. Wincker, The banana (*Musa acuminata*) genome and the evolution of monocotyledonous plants. *Nature* **488**, 213–217 (2012).

134. N. Bornowski, K. J. Michel, J. P. Hamilton, S. Ou, A. S. Seetharam, J. Jenkins, J. Grimwood, C. Plott, S. Shu, J. Talag, M. Kennedy, H. Hundley, V. R. Singan, K. Barry, C. Daum, Y. Yoshinaga, J. Schmutz, C. N. Hirsch, M. B. Hufford, N. de Leon, S. M. Kaeppler, C. R. Buell, Genomic variation within the maize stiff-stalk heterotic germplasm pool. *Plant Genome* **14**, e20114 (2021).

135. H. Sakai, S. S. Lee, T. Tanaka, H. Numa, J. Kim, Y. Kawahara, H. Wakimoto, C.-C. Yang, M. Iwamoto, T. Abe, Y. Yamada, A. Muto, H. Inokuchi, T. Ikemura, T. Matsumoto, T. Sasaki, T. Itoh, Rice Annotation Project Database (RAP-DB): An integrative and interactive database for rice genomics. *Plant Cell Physiol.* **54**, e6 (2013).

136. M. Mascher, T. Wicker, J. Jenkins, C. Plott, T. Lux, C. S. Koh, J. Ens, H. Gundlach, L. B. Boston, Z. Tulpová, S. Holden, I. Hernández-Pinzón, U. Scholz, K. F. X. Mayer, M. Spannagl, C. J. Pozniak, A. G. Sharpe, H. Šimková, M. J. Moscou, J. Grimwood, J. Schmutz, N. Stein, Long-read sequence assembly: A technical evaluation in barley. *Plant Cell* **33**, 1888–1906 (2021).

137. J. M. McGrath, A. Funk, P. Galewski, S. Ou, B. Townsend, K. Davenport, H. Daligault, S. Johnson, J. Lee, A. Hastie, A. Darracq, G. Willems, S. Barnes, I. Liachko, S. Sullivan, S. Koren, A. Phillippy, J. Wang, T. Liu, J. Pulman, K. Childs, S. Shu, A. Yocum, D. Fermin, E. Mutasa-Göttgens, P. Stevanato, K. Taguchi, R. Naegele, K. M. Dorn, A contiguous *de novo* genome assembly of sugar beet EL10 (*Beta vulgaris* L.). *DNA Res.* **30**, dsac033 (2023).

138. R. van Velzen, R. Holmer, F. Bu, L. Rutten, A. van Zeijl, W. Liu, L. Santuari, Q. Cao, T. Sharma, D. Shen, Y. Roswanjaya, T. A. K. Wardhani, M. S. Kalhor, J. Jansen, J. van den Hoogen, B. Güngör, M. Hartog, J. Hontelez, J. Verver, W.-C. Yang, E. Schijlen, R. Repin, M. Schilthuizen, M. E. Schranz, R. Heidstra, K. Miyata, E. Fedorova, W. Kohlen, T. Bisseling, S. Smit, R. Geurts, Comparative genomics of the nonlegume Parasponia reveals insights into evolution of nitrogen-fixing rhizobium symbioses. *Proc. Natl. Acad. Sci. U.S.A.* **115**, E4700–E4709 (2018).

139. M. Griesmann, Y. Chang, X. Liu, Y. Song, G. Haberer, M. B. Crook, B. Billault-Penneteau, D. Lauressergues, J. Keller, L. Imanishi, Y. P. Roswanjaya, W. Kohlen, P. Pujic, K. Battenberg, N. Alloisio, Y. Liang, H. Hilhorst, M. G. Salgado, V. Hocher, H. Gherbi, S. Svistoonoff, J. J. Doyle, S. He, Y. Xu, S. Xu, J. Qu, Q. Gao, X. Fang, Y. Fu, P. Normand, A. M. Berry, L. G. Wall, J.-M. Ané, K. Pawlowski, X. Xu, H. Yang, M. Spannagl, K. F. X. Mayer, G. K.-S. Wong, M. Parniske, P.-M. Delaux, S. Cheng, Phylogenomics reveals multiple losses of nitrogen-fixing root nodule symbiosis. *Science* **361**, eaat1743 (2018).

140. B. Hufnagel, A. Marques, A. Soriano, L. Marquès, F. Divol, P. Doumas, E. Sallet, D. Mancinotti, S. Carrere, W. Marande, S. Arribat, J. Keller, C. Huneau, T. Blein, D. Aimé, M. Laguerre, J. Taylor, V. Schubert, M. Nelson, F. Geu-Flores, M. Crespi, K. Gallardo, P.-M. Delaux, J. Salse, H. Bergès, R. Guyot, J. Gouzy, B. Péret, High-quality genome sequence of white lupin provides insight into soil exploration and seed quality. *Nat. Commun.* **11**, 492 (2020).

141. N. D. Young, F. Debellé, G. E. D. Oldroyd, R. Geurts, S. B. Cannon, M. K. Udvardi, V. A. Benedito, K. F. X. Mayer, J. Gouzy, H. Schoof, Y. Van de Peer, S. Proost, D. R. Cook, B. C. Meyers, M. Spannagl, F. Cheung, S. De Mita, V. Krishnakumar, H. Gundlach, S. Zhou, J. Mudge, A. K. Bharti, J. D. Murray, M. A. Naoumkina, B. Rosen, K. A. T. Silverstein, H. Tang, S. Rombauts, P. X. Zhao, P. Zhou, V. Barbe, P. Bardou, M. Bechner, A. Bellec, A. Berger, H. Bergès, S. Bidwell, T. Bisseling, N. Choisne, A. Couloux, R. Denny, S. Deshpande, X. Dai, J. J. Doyle, A.-M. Dudez, A. D. Farmer, S. Fouteau, C. Franken, C. Gibelin, J. Gish, S. Goldstein, A. J. González, P. J. Green, A. Hallab, M. Hartog, A. Hua, S. J. Humphray, D.-H. Jeong, Y. Jing, A. Jöcker, S. M. Kenton, D.-J. Kim, K. Klee, H. Lai, C. Lang, S. Lin, S. L. Macmil, G. Magdelenat, L. Matthews, J. McCorrison, E. L. Monaghan, J.-H. Mun, F. Z. Najar, C. Nicholson, C. Noirot, M. O'Bleness, C. R. Paule, J. Poulain, F. Prion, B. Qin, C. Qu, E. F. Retzel, C. Riddle, E. Sallet, S. Samain, N. Samson, I. Sanders, O. Saurat, C. Scarpelli, T. Schiex, B. Segurens, A. J. Severin, D. J. Sherrier, R. Shi, S. Sims, S. R. Singer, S. Sinharoy, L. Sterck, A. Viollet, B.-B. Wang, K. Wang, M. Wang, X. Wang, J. Warfsmann, J. Weissenbach, D. D. White, J. D. White, G. B. Wiley, P. Wincker, Y. Xing, L. Yang, Z. Yao, F. Ying, J. Zhai, L. Zhou, A. Zuber, J. Dénarié, R. A. Dixon, G. D. May, D. C. Schwartz, J. Rogers, F. Quétier, C. D. Town, B. A. Roe, The *Medicago* genome provides insight into the evolution of rhizobial symbioses. *Nature* **480**, 520–524 (2011).

142. J. Schmutz, S. B. Cannon, J. Schlueter, J. Ma, T. Mitros, W. Nelson, D. L. Hyten, Q. Song, J. J. Thelen, J. Cheng, D. Xu, U. Hellsten, G. D. May, Y. Yu, T. Sakurai, T. Umezawa, M. K. Bhattacharyya, D. Sandhu, B. Valliyodan, E. Lindquist, M. Peto, D. Grant, S. Shu, D. Goodstein, K. Barry, M. Futrell-Griggs, B. Abernathy, J. Du, Z. Tian, L. Zhu, N. Gill, T. Joshi, M. Libault, A. Sethuraman, X.-C. Zhang, K. Shinozaki, H. T. Nguyen, R. A. Wing, P. Cregan, J. Specht, J. Grimwood, D. Rokhsar, G. Stacey, R. C. Shoemaker, S. A. Jackson, Genome sequence of the palaeopolyploid soybean. *Nature* **463**, 178–183 (2010).

143. N. Kamal, T. Mun, D. Reid, J.-S. Lin, T. Y. Akyol, N. Sandal, T. Asp, H. Hirakawa, J. Stougaard, K. F. X. Mayer, S. Sato, S. U. Andersen, Insights into the evolution of symbiosis gene copy number and distribution from a chromosome-scale *Lotus japonicus* Gifu genome sequence. *DNA Res.* **27** (2020), 10.1093/dnares/dsaa015.

144. N. Fernandez-Pozo, N. Menda, J. D. Edwards, S. Saha, I. Y. Tecle, S. R. Strickler, A. Bombarely, T. Fisher-York, A. Pujar, H. Foerster, A. Yan, L. A. Mueller, The Sol Genomics Network (SGN)—From genotype to phenotype to breeding. *Nucleic Acids Res.* **43**, D1036–D1041 (2015).

145. Tomato Genome Consortium, The tomato genome sequence provides insights into fleshy fruit evolution. *Nature* **485**, 635–641 (2012).

146. G. M. Pham, J. P. Hamilton, J. C. Wood, J. T. Burke, H. Zhao, B. Vaillancourt, S. Ou, J. Jiang, C. R. Buell, Construction of a chromosome-scale long-read reference genome assembly for potato. *Gigascience* **9**, giaa100 (2020).

147. P. Lamesch, T. Z. Berardini, D. Li, D. Swarbreck, C. Wilks, R. Sasidharan, R. Muller, K. Dreher, D. L. Alexander, M. Garcia-Hernandez, A. S. Karthikeyan, C. H. Lee, W. D. Nelson, L. Ploetz, S. Singh, A. Wensel, E. Huala, The Arabidopsis Information Resource (TAIR): Improved gene annotation and new tools. *Nucleic Acids Res.* **40**, D1202–10 (2012).

148. X. Argout, J. Salse, J.-M. Aury, M. J. Guiltinan, G. Droc, J. Gouzy, M. Allegre, C. Chaparro, T. Legavre, S. N. Maximova, M. Abrouk, F. Murat, O. Fouet, J. Poulain, M. Ruiz, Y. Roguet, M. Rodier-Goud, J. F. Barbosa-Neto, F. Sabot, D. Kudrna, J. S. S. Ammiraju, S. C. Schuster, J. E.

Carlson, E. Sallet, T. Schiex, A. Dievart, M. Kramer, L. Gelley, Z. Shi, A. Bérard, C. Viot, M. Boccara, A. M. Risterucci, V. Guignon, X. Sabau, M. J. Axtell, Z. Ma, Y. Zhang, S. Brown, M. Bourge, W. Golser, X. Song, D. Clement, R. Rivallan, M. Tahi, J. M. Akaza, B. Pitollat, K. Gramacho, A. D'Hont, D. Brunel, D. Infante, I. Kebe, P. Costet, R. Wing, W. R. McCombie, E. Guiderdoni, F. Quetier, O. Panaud, P. Wincker, S. Bocs, C. Lanaud, The genome of *Theobroma cacao*. *Nat. Genet.* **43**, 101–108 (2011).

149. R. Ming, S. Hou, Y. Feng, Q. Yu, A. Dionne-Laporte, J. H. Saw, P. Senin, W. Wang, B. V. Ly, K. L. T. Lewis, S. L. Salzberg, L. Feng, M. R. Jones, R. L. Skelton, J. E. Murray, C. Chen, W. Qian, J. Shen, P. Du, M. Eustice, E. Tong, H. Tang, E. Lyons, R. E. Paull, T. P. Michael, K. Wall, D. W. Rice, H. Albert, M.-L. Wang, Y. J. Zhu, M. Schatz, N. Nagarajan, R. A. Acob, P. Guan, A. Blas, C. M. Wai, C. M. Ackerman, Y. Ren, C. Liu, J. Wang, J. Wang, J.-K. Na, E. V. Shakirov, B. Haas, J. Thimmapuram, D. Nelson, X. Wang, J. E. Bowers, A. R. Gschwend, A. L. Delcher, R. Singh, J. Y. Suzuki, S. Tripathi, K. Neupane, H. Wei, B. Irikura, M. Paidi, N. Jiang, W. Zhang, G. Presting, A. Windsor, R. Navajas-Pérez, M. J. Torres, F. A. Feltus, B. Porter, Y. Li, A. M. Burroughs, M.-C. Luo, L. Liu, D. A. Christopher, S. M. Mount, P. H. Moore, T. Sugimura, J. Jiang, M. A. Schuler, V. Friedman, T. Mitchell-Olds, D. E. Shippen, C. W. dePamphilis, J. D. Palmer, M. Freeling, A. H. Paterson, D. Gonsalves, L. Wang, M. Alam, The draft genome of the transgenic tropical fruit tree papaya (*Carica papaya* Linnaeus). *Nature* **452**, 991–996 (2008).

150. G. A. Wu, S. Prochnik, J. Jenkins, J. Salse, U. Hellsten, F. Murat, X. Perrier, M. Ruiz, S. Scalabrin, J. Terol, M. A. Takita, K. Labadie, J. Poulain, A. Couloux, K. Jabbari, F. Cattonaro, C. Del Fabbro, S. Pinosio, A. Zuccolo, J. Chapman, J. Grimwood, F. R. Tadeo, L. H. Estornell, J. V. Muñoz-Sanz, V. Ibanez, A. Herrero-Ortega, P. Aleza, J. Pérez-Pérez, D. Ramón, D. Brunel, F. Luro, C. Chen, W. G. Farmerie, B. Desany, C. Kodira, M. Mohiuddin, T. Harkins, K. Fredrikson, P. Burns, A. Lomsadze, M. Borodovsky, G. Reforgiato, J. Freitas-Astúa, F. Quetier, L. Navarro, M. Roose, P. Wincker, J. Schmutz, M. Morgante, M. A. Machado, M. Talon, O. Jaillon, P. Ollitrault, F. Gmitter, D. Rokhsar, Sequencing of diverse mandarin, pummelo and orange genomes reveals complex history of admixture during citrus domestication. *Nat. Biotechnol.* **32**, 656–662 (2014).
